# Fluorescence Visualization of Helix Inversion in Biomimic Polymeric Foldamer

**DOI:** 10.1002/anie.202512834

**Published:** 2025-10-29

**Authors:** Yuan Qiu, Zonghang Liu, Chenchen Sun, Xilong Wei, Ryan T. K. Kwok, Yonggui Liao, Jianwei Sun, Jacky W. Y. Lam, Zijie Qiu, Xiaolin Xie, Ben Zhong Tang

**Affiliations:** ^1^ Department of Chemistry the Hong Kong Branch of Chinese National Engineering Research Center for Tissue Restoration and Reconstruction The Hong Kong University of Science and Technology Kowloon Hong Kong P.R. China; ^2^ Guangdong Basic Research Center of Excellence for Aggregate Science School of Science and Engineering The Chinese University of Hong Kong (Shenzhen) Longgang, Shenzhen, Guangdong 518172 P.R. China; ^3^ Key Laboratory of Material Chemistry for Energy Conversion and Storage Ministry of Education Hubei Key Laboratory of Material Chemistry and Service Failure Hubei Engineering Research Center for Biomaterials and Medical Protective Materials School of Chemistry and Chemical Engineering Huazhong University of Science and Technology Wuhan 430074 P.R. China

**Keywords:** Artificial foldamer, Fluorescence visualization, Helix inversion, Intramolecular charge transfer

## Abstract

Biomacromolecules such as DNA, proteins, and polysaccharides possess unique helical structures that are closely related to their biological functions involving recognition, catalysis, replication, and genetic information storage. From a biomimetic perspective, artificial foldamers are the ideal systems to model and study the structure–property relationship of biomacromolecules. Herein, we report a facile, rapid, and cost‐effective method to directly visualize and monitor the solvent‐driven helix inversion in a water‐soluble poly(*m*‐phenylene ethynylene)‐based foldamer with the aid of a tetraphenylethene‐functionalized hemicyanine dye. By adjusting the solvent environment, the foldamer transitions from an *M*‐helix to a *P*‐helix, accompanied by a change in the dye's binding mode from groove to surface. This transition alters the degree of restriction and microenvironment polarity, resulting in a visible emission color change from yellow to red, signaling the helix inversion. The present investigation offers a powerful tool for understanding conformational transitions in biomacromolecules and offers insights into the dynamic behavior of helical structures.

## Graphical abstract

A facile, rapid, and economical method is provided to directly visualize the solvent‐driven helix inversion in a water‐soluble foldamer using a tetraphenylethene‐functionalized hemicyanine dye based on the emission color change from yellow to red. The dyes are first restricted in the low‐polarity groove but then docked on the foldamer surface with the less restricted molecular motions and the enhanced intramolecular charge transfer.

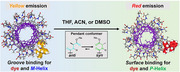

## Introduction

**Figure 1:**
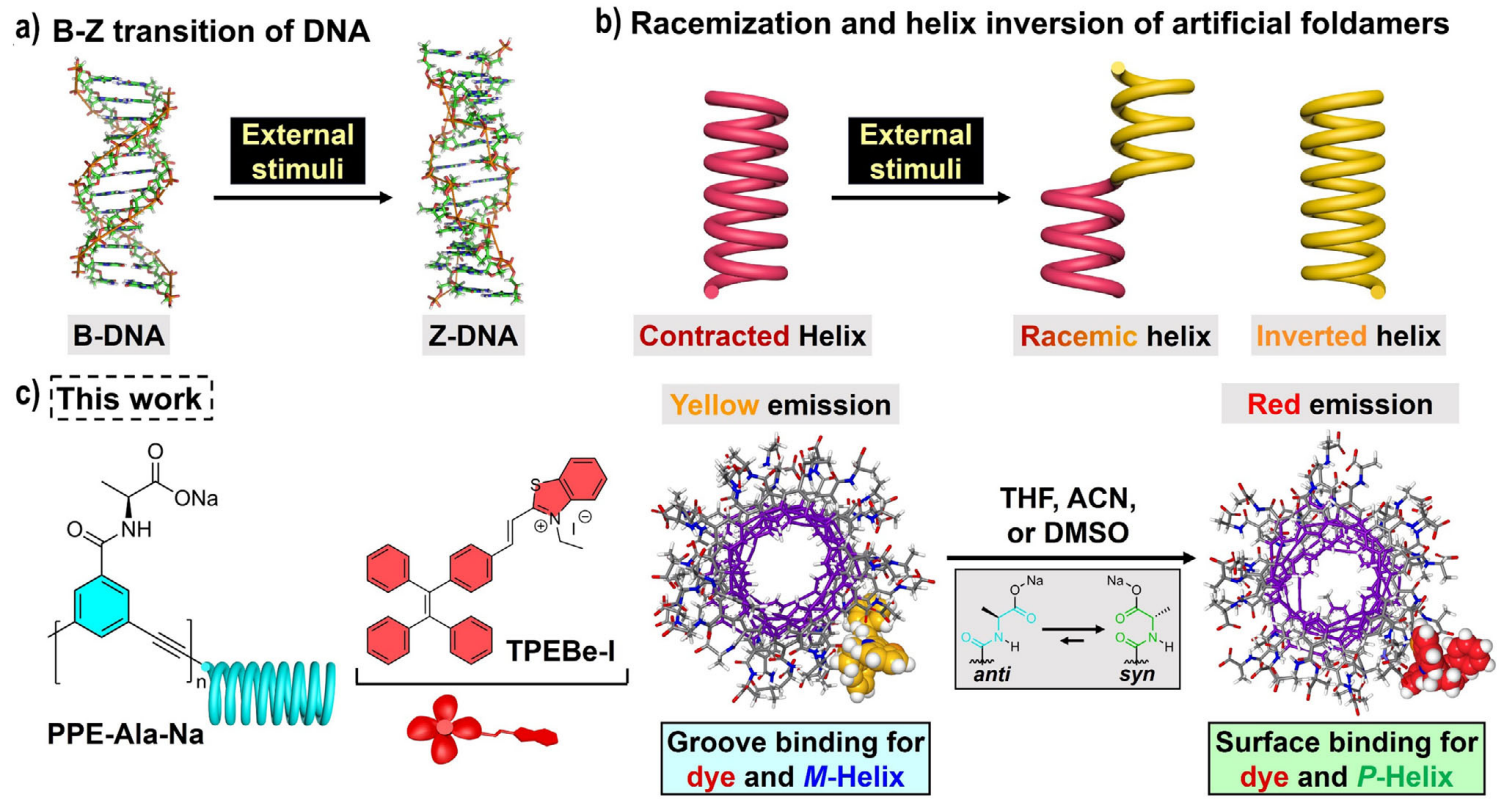
a) Illustration of B–Z transition of DNA triggered by external stimuli. b) Illustration of racemization and helix inversion of artificial foldamers triggered by external stimuli. c) Illustration of color‐switchable fluorescence visualization of solvent‐triggered helix inversion of **PPE‐Ala‐Na** assisted by **TPEBe‐I**.

Biomacromolecules, such as DNA, proteins, and polysaccharides, feature unique helical structures. These structures are intimately linked to various biological activities, including recognition, catalysis, replication, genetic information storage, etc.^[^ ^1^, ^2^, ^3^ ^]^ In particular, the screw sense of these biomacromolecules strongly influences all kinds of biological functions.^[^ ^4^ ^]^ For example, a right‐handed DNA double helix (B form) inverts to a left‐handed one (Z form) upon interacting with biological amines/metal cations, increasing salt concentration, or elevating temperature (Figure 1a),^[^ ^5^, ^6^ ^]^ which causes genetic instability and hence leads to human diseases.^[^ ^7^, ^8^ ^]^ From the perspective of biomimetics, artificial foldamers have garnered great interest because they can model the folding behavior that occurs in the secondary structure of biomacromolecules and their helix screw sense can be easily modulated.^[^ ^9^ ^]^ Circular dichroism (CD) spectroscopy is widely used to determine this conformational transition on the basis of the reversal of the Cotton effect sign.^[^ ^10^, ^11^, ^12^, ^13^ ^]^ Although the detection via such a spectroscopy method is important, a direct observation of single foldamer chains or a color change of foldamer solutions is more intuitive.^[^ ^14^ ^]^ The direct observation of the helical structures, including the helical pitch and handedness, can be achieved by high‐resolution atomic force microscopy (AFM).^[^ ^15^, ^16^ ^]^ However, the AFM instrument is generally expensive, and its sample preparations are time‐consuming and troublesome. Therefore, it is of great value to develop a facile, rapid, and economical method to directly visualize and monitor helix inversion behaviors.

Usually, artificial foldamers can undergo racemization and even helix inversion owing to various external stimuli, including temperature,^[^ ^17^ ^]^ light irradiation,^[^ ^18^ ^]^ solvent,^[^ ^19^ ^]^ ions^[^ ^20^, ^21^, ^22^, ^23^ ^]^ and chiral additives (Figure 1b).^[^ ^24^, ^25^ ^]^ For example, a poly(*m*‐ethynylpyridine) derivative shows a positive CD signal, which decreases and turns negative as the temperature is increased from 25 °C to 70 °C, along with a hypochromism and a blue‐shift in the main chain absorption due to the promotion of the π–π stacking interactions.^[^ ^17^ ^]^ A small amount of a single enantiomeric diamine can induce a contracted right‐handed helix in a poly(*ortho*‐phenylene ethynylene)‐based foldamer bearing carboxylic pendants via the 1:2 binding mode, while an extended left‐handed helical conformation is obtained because the binding mode changes into a 1:1 type upon the further addition of the diamine.^[^ ^24^ ^]^ Besides the helix inversion, these artificial foldamers also regulate the folded helical conformations by changing the pitch distances in response to external stimuli, leading to a helix extension or contraction.^[^ ^26^ ^]^ Subsequently, these conformational transitions of artificial foldamers presumably can cause a change in the local environments of the groove and surface of the foldamers.

Aggregation‐induced emission luminogens (AIEgens) are ideal fluorescent probes to visualize the subtle changes in the local environment.^[^ ^27^ ^]^ Upon incorporating electron‐donating (D) and ‐accepting (A) units into AIEgens, they are endowed with high sensitivity and multicolor response to the local environment polarity change owing to the intramolecular charge transfer (ICT) effect.^[^ ^28^ ^]^ Therefore, AIEgens have been used as the visualizing agents to monitor various dynamic processes, including hygroscopicity and desorption,^[^ ^29^, ^30^, ^31^, ^32^ ^]^ microphase separation,^[^ ^33^ ^]^ crystallization,^[^ ^34^ ^]^ and physiological temperature variation.^[^ ^35^ ^]^ Inspiringly, the helix‐coil transition and helix extension of a water‐soluble poly(*m*‐phenylene diethynylene)‐based foldamer have been successfully visualized using a tetraphenylethylene (TPE)‐functionalized hemicyanine dye.^[^ ^36^ ^]^ Therefore, we are prompted to achieve the direct visualization of helix inversion in water‐soluble artificial foldamers and offer insights into the dynamic behavior of biomacromolecules.

In this work, the visualization of solvent‐driven helix inversion of a water‐soluble helical poly(*m*‐phenylene ethynylene)‐based foldamer (**PPE‐Ala‐Na**) is reported (Figure 1c). When the TPE‐functionalized hemicyanine dye (**TPEBe‐I**) is added to the aqueous solution of (*M*)‐handed **PPE‐Ala‐Na**, **TPEBe‐I** is inserted into the foldamer groove via electrostatic interaction and hydrophobic effect. The low polarity of the groove induces the bright yellow emission of **TPEBe‐I**. In the presence of tetrahydrofuran (THF), acetonitrile (ACN), and dimethyl sulfoxide (DMSO), the foldamer forms an extended (*P*)‐handed helix because of the *anti* to *syn* conformer change of chiral pendants. The dyes are isolated and subsequently docked on the foldamer surface, leading to less restricted intramolecular motions and enhanced charge transfer. Therefore, an emission color change from yellow to red is observed, realizing a color‐switchable fluorescence visualization in the polymeric foldamer.

## Results and Discussion

### The Luminescent Behavior of TPEBe‐I

**Figure 2:**
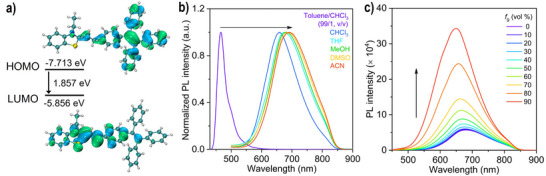
a) Molecular orbitals of **TPEBe‐I** calculated by DFT calculations at the B3LYP/6‐31G(d,p) level using the Gaussian 09 program. b) Fluorescence spectra of **TPEBe‐I** in solvents with different polarities, including toluene/CHCl_3_ (99/1, v/v), CHCl_3_, THF, MeOH, DMSO, and ACN, at room temperature. c) Fluorescence spectra of **TPEBe‐I** in MeOH/glycerol mixtures with different volume fractions of glycerol (*f*_g_) at room temperature. [**TPEBe‐I**] = 10 µM; excitation wavelength (*λ*_ex_): 420 nm.

A previously reported TPE‐functionalized hemicyanine dye, **TPEBe‐I**, is selected to visualize the solvent‐triggered helix inversion behavior of polymeric foldamer (Figure 1c).^[^ ^37^ ^]^ The dye is soluble in dichloromethane, chloroform (CHCl_3_), THF, methanol, ACN, and DMSO, but insoluble in toluene and water. The electron‐donating TPE and the electron‐withdrawing benzothiazolium salt units are connected through the ethylene core, which endows the dye with a typical D−π−A structure. In Figure 2a, the highest occupied molecular orbital (HOMO) is mainly located on the TPE unit, while the lowest unoccupied molecular orbital (LUMO) is mainly concentrated on the benzothiazolium salt unit. Such an electron separation reveals a π−π* transition with a charge transfer character. In Figure 2b, **TPEBe‐I** features an emission peak at 465 nm in toluene/chloroform at a volume ratio of 99/1, which is assigned to the locally excited (LE) state.^[^ ^38^ ^]^ With the increase of solvent polarity, the emission color shifts from blue to red in chloroform, THF, methanol, DMSO, and ACN (*λ*_em_ = 658–690 nm). Clearly, this polarity‐dependent behavior demonstrates the ICT characteristic of **TPEBe‐I**. The emission spectra of **TPEBe‐I** in toluene/chloroform mixtures show that the emission peak at 658 nm gradually blueshifts and then disappears, but the peak at 465 nm appears as the nonpolar toluene fraction increases (Figure S1), further confirming the ICT effect of **TPEBe‐I**. Subsequently, the emission spectra of the dye in methanol/glycerol mixtures are measured (Figure 2c). With the increasing solvent viscosity, the emission is sharply enhanced, revealing that the dye is sensitive to viscosity changes due to the restriction of intramolecular motion (RIM) mechanism.^[^ ^39^ ^]^ Therefore, the fluorescent characteristics endow **TPEBe‐I** to be a promising fluorescent probe in response to the changes in polymer microenvironments.

### Synthesis of Foldamer PPE‐Ala‐Na

**Figure 3:**
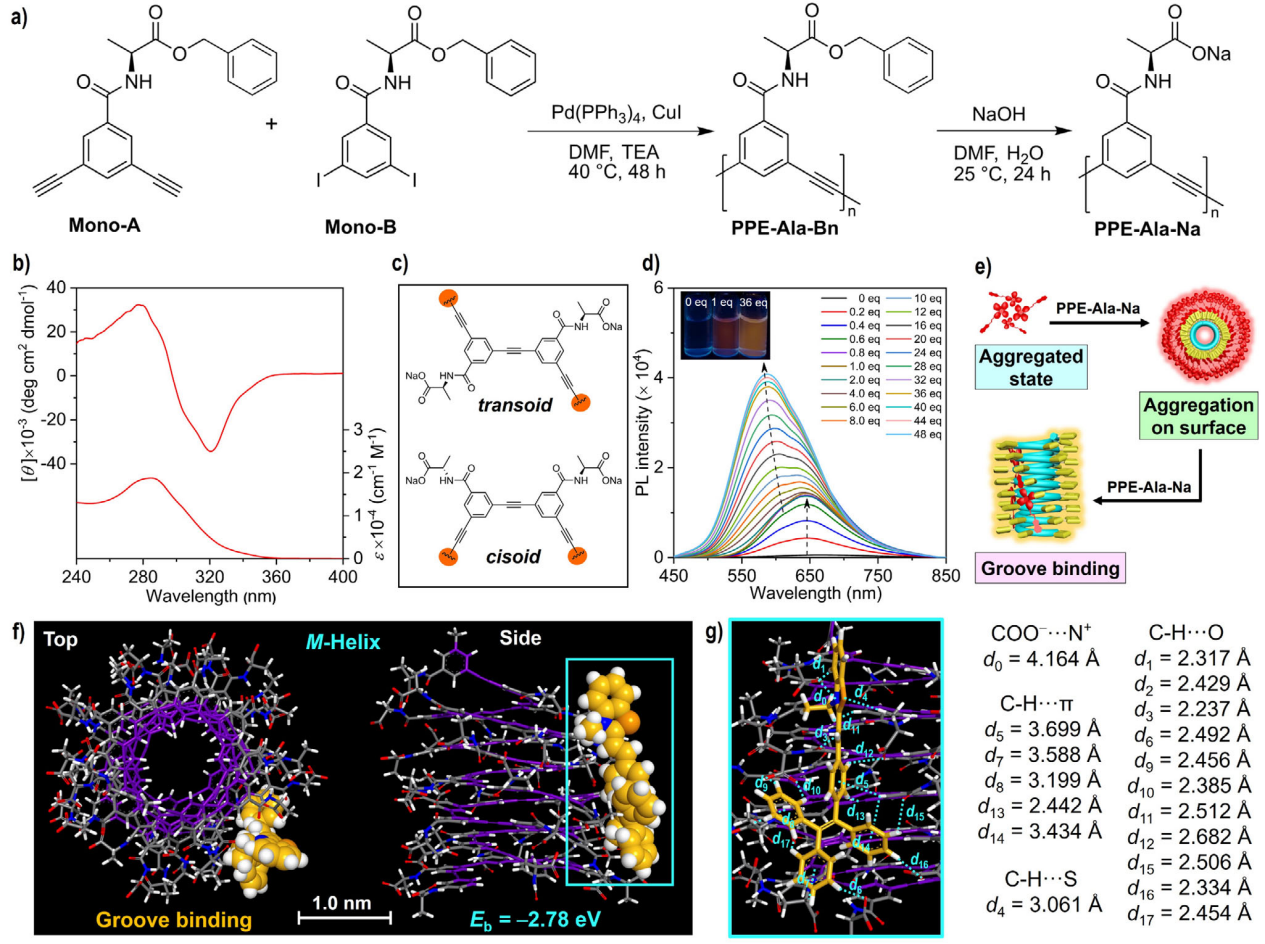
a) Synthetic route for foldamer **PPE‐Ala‐Na**. b) CD (top) and UV−vis absorption (bottom) spectra of **PPE‐Ala‐Na** in water at room temperature. [**PPE‐Ala‐Na**] = 100 µM. c) *Transoid* and *cisoid* conformers of phenylene ethynylene chromophores in **PPE‐Ala‐Na**. d) Fluorescence spectra of **TPEBe‐I** in the presence of **PPE‐Ala‐Na** in water/THF (99/1, v/v) at room temperature. [**TPEBe‐I**] = 1.0 µM; excitation wavelength (*λ*_ex_): 420 nm. Inset: Fluorescent photographs of **TPEBe‐I** with 0, 1, and 36 equiv **PPE‐Ala‐Na** under 365 nm UV irradiation. e) Illustration of two‐stage binding behavior of **TPEBe‐I** with **PPE‐Ala‐Na**. f) Top and side views of a possible model of groove binding of **TPEBe‐I** with the (*M*)‐handed helical **PPE‐Ala‐Na** in water/THF (99/1, v/v) optimized by DFT calculations. The structures are represented by capped‐stick models except for the **TPEBe‐I** molecules, which are highlighted as space‐filling ones. The carbon atoms of the helical main chain are highlighted in purple. g) Enlarged model corresponding to the areas indicated by the blue square in (f). Noncovalent interactions are indicated by blue dashed lines in (g). The **TPEBe‐I** molecule is represented by the capped‐stick model.

The foldamer **PPE‐Ala‐Na** is synthesized according to the route illustrated in Figure 3a. First, two chiral monomers, **Mono‐A** and **Mono‐B**, are synthesized via amidation reaction (Scheme S1). Then, they are polymerized into the precursor **PPE‐Ala‐Bn** through Sonagashira coupling reaction using Pd(PPh_3_)_4_ and CuI as co‐catalysts under an argon atmosphere. Its number‐average molecular weight (*M*_n_) and molecular weight dispersity (*M*_w_/*M*_n_) by gel permeation chromatography (GPC) are determined to be 4.4 × 10^4^ and 1.43, respectively. Its number‐average degree of polymerization (*N*) is calculated to be ∼145. Finally, the hydrolysis of **PPE‐Ala‐Bn** affords the target foldamer **PPE‐Ala‐Na** in alkaline medium. The *N* for **PPE‐Ala‐Na** is considered to be the same as its precursor because the hydrolysis reaction conditions are mild to the backbone structure.^[^ ^40^ ^]^ The foldamer is insoluble in dichloromethane, chloroform, THF, and ACN but partially soluble in DMSO, while exhibiting good solubility in water. The detailed synthesis and characterization are provided in Figures S2–S13.

### Binding Behavior Between TPEBe‐I and PPE‐Ala‐Na

Theoretically, **PPE‐Ala‐Na** can undergo helical folding in water by the hydrophobic effect due to its amphiphilic nature.^[^ ^41^ ^]^ Meanwhile, the screw‐sense bias is directed by L‐alaninate sodium pendants.^[^ ^16^ ^]^ The resulting folded helix can be closely related to the variable binding modes owing to its multiple binding sites, including negatively charged pendants, helical grooves, and cavities. From the CD and UV−vis spectra, the foldamer exhibits a significant absorption peak at 285 nm (Figure 3b), corresponding to the π‐conjugated phenylene ethynylene chromophores with a *cisoid* conformer (Figure 3c).^[^ ^15^ ^]^ However, the absorption peak arising from the *transoid* conformer, which often appears at wavelengths greater than 300 nm,^[^ ^42^ ^]^ cannot be observed. In other words, the foldamer exists as a fully folded state in water.^[^ ^43^ ^]^ In addition, a strong bisignated Cotton effect due to the foldamer main chain is found with negative and positive maxima at 320 and 278 nm, respectively (Figures 3b and S14). In combination with wide‐angle X‐ray scattering (WAXS), molecular dynamics (MD) simulation, and calculated CD spectrum, it is concluded that this foldamer features a (*M*)‐handed helix with a helical pitch of ~3.42 Å, an outer diameter of ~27.1 Å, and an inner diameter of ~7.36 Å (Figures S15–S18). Interestingly, this pitch value is the same as that of the previously reported poly(*m*‐phenylene diethynylene)‐based foldamer.^[^ ^36^ ^]^

The positively charged hemicyanine dye **TPEBe‐I** is employed as a visualizing agent to bind with the negatively charged polymeric foldamer (Figure S19). Given that the dye is poorly soluble in pure water, the water/THF mixture at a volume ratio of 99/1 is chosen as the solvent for the subsequent investigations (Figure S20). The foldamer still possesses a fully folded (*M*)‐handed helix at this ratio (Figure S32). As the foldamer concentration increases from 0 to 1 equiv, the emission intensity of the dye at 648 nm sharply increases (Figures 3d, S21, and S22). Accordingly, at the **TPEBe‐I**/**PPE‐Ala‐Na** ratio of 1/1, each positively charged dye can bind to one L‐alanine sodium pendant by electrostatic interactions, forming the aggregation complex on the foldamer surface (Figures 3e and S23). As a result, red emission arises because the restricted intramolecular motions of the dyes prohibit the nonradiative relaxation channel.^[^ ^27^ ^]^

When the foldamer concentration increases from 2 equiv to 48 equiv, a new emission peak appears at 588 nm, whose intensity gradually increases and reaches a plateau value at a **TPEBe‐I**/**PPE‐Ala‐Na** ratio of 1/36. **PPE‐Ala‐Na** possesses 24 turns based on the number‐average degree of polymerization *N* = 145 and ∼6 monomer units per turn (Figure S16). Since the binding ratio of **PPE‐Ala‐Na** to **TPEBe‐I** is 36/1 at the equilibrium state (Figure S22), each foldamer helix can entrap four dyes (Figures 3e and S23). The presence of an emission peak at 588 nm and the decrease of the relative proportion of an emission peak at 648 nm imply that the dyes continuously enter a local environment with a lower polarity relative to THF and water,^[^ ^44^ ^]^ as supported by the emission spectral changes of the dye in toluene/chloroform mixtures (Figure S1). Therefore, it's proposed that the newly added foldamer helices provide a large number of anion binding sites to attract the dyes, which tend to insert into the hydrophobic groove or cavity with a low‐polarity environment via electrostatic interaction and hydrophobic effect.^[^ ^37^ ^]^

MD simulations are performed to determine the binding mode between **TPEBe‐I** and **PPE‐Ala‐Na** (Movie S1 and Section 7 in Supporting Information). It is found that the (*M*)‐handed helical main chain is always maintained during the simulation, and the dye is located in the foldamer groove after equilibration (Figure S24). However, the dye cannot be further inserted into the foldamer cavity because the TPE unit with a diagonal length of 11.5 Å^[^ ^45^ ^]^ in **TPEBe‐I** does not match the small cavity size of ~7.36 Å (Figure S17). To obtain the detailed information about the **TPEBe‐I**/**PPE‐Ala‐Na** complex in water/THF (99/1, v/v), its geometry optimization is further conducted by DFT calculation (Figure 3f), where their intermolecular noncovalent interactions are visualized and quantitatively evaluated using the Hirshfeld partition of molecular density (IGMH)^[^ ^46^ ^]^ and the electron density topology analysis (QTAIM)^[^ ^47^ ^]^ analyses, respectively (Figure S25 and Table S1). Various noncovalent forces, including COO^−^⋯N^+^, C─H⋯π, C─H⋯S, and C─H⋯O interactions, are present in the **TPEBe‐I**/**PPE‐Ala‐Na** complex (Figure 3g). Specifically, a hydrogen atom and two benzene rings in the TPE unit can form C─H⋯π interactions (*d*_13_, *d*_5_, and *d*_14_) at close distances (2.442, 3.699, and 3.434 Å) with the alkynyl group and the phenyl hydrogen atom in the foldamer main chain, respectively. Such a deep insertion into the groove can create a low‐polarity environment despite the aqueous solvent. With these diverse interactions, the intramolecular motions and the intramolecular charge transfer of the dye are effectively restricted, endowing enhanced emission and the appearance of the yellow emission peak at 587 nm (Figure 3d). Therefore, during the binding process between **TPEBe‐I** and **PPE‐Ala‐Na**, the emission color changes from colorless to red and then to yellow under 365 nm UV irradiation (Figure 3d, inset). The red emission for the dye is derived from the surface binding at the first stage, while its yellow emission is ascribed to the groove binding at the second stage. As a result, the varied binding behavior between **PPE‐Ala‐Na** and **TPEBe‐I** is directly distinguished by the emission color changes of **TPEBe‐I**.

### Visualization of Helix Inversion in PPE‐Ala‐Na in Water/THF Mixtures

**Figure 4:**
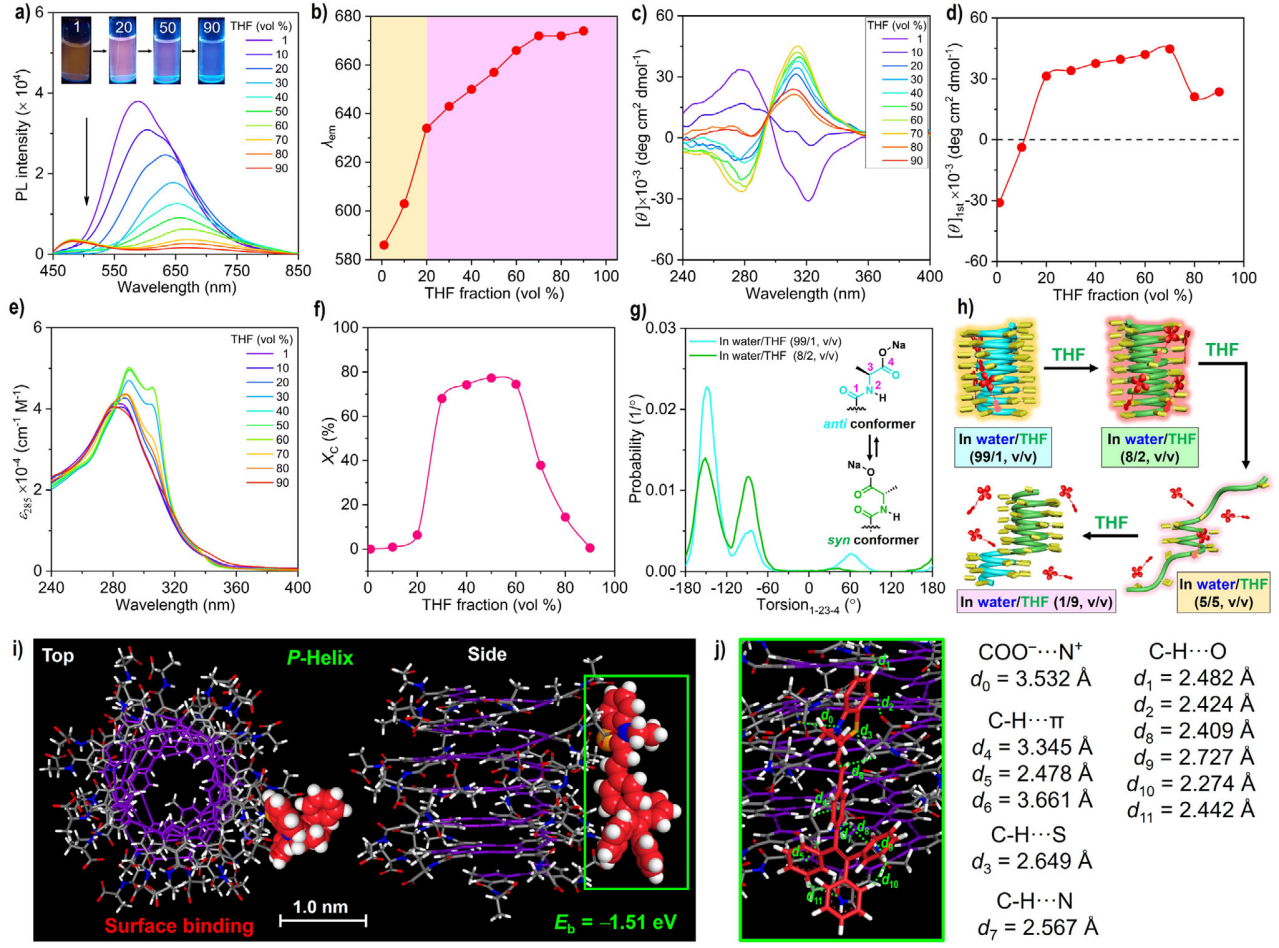
a) Fluorescence spectra of **TPEBe‐I** with **PPE‐Ala‐Na** in water/THF mixtures with different organic solvent fractions. [**TPEBe‐I**] = 1.0 µM; [**PPE‐Ala‐Na**] = 36 µM. Inset: Fluorescent photographs of **TPEBe‐I** in water/THF mixtures with 1, 20, 50, and 90 vol% THF under 365 nm UV irradiation. b) Plot of the emission wavelength (*λ*_em_) of **TPEBe‐I** with **PPE‐Ala‐Na** versus THF fraction. c) and e) CD (c) and UV−vis absorption (e) spectra of **PPE‐Ala‐Na** in the presence of **TPEBe‐I** in water/THF mixtures. d) and f) Plots of the first Cotton effect intensity (d) and the content of random‐coil conformation (f) versus the volume fraction of THF. g) Probability distribution functions of the OC─N─C─CO (1–23–4) dihedral angle of L‐alanine sodium pendants in the model structures of the (*M*)‐handed and (*P*)‐handed helical **PPE‐Ala‐Na** complexed with **TPEBe‐I** in water/THF (99/1, v/v) and water/THF (80/20, v/v), respectively. Inset: Representative *anti* and *syn* orientations between two carbonyl groups of l‐alanine sodium pendants. h) Illustrative conformational transitions of **PPE‐Ala‐Na** in the presence of **TPEBe‐I** in water/THF mixtures. i) Top and side views of a possible model of surface binding of **TPEBe‐I** with the (*P*)‐handed helical **PPE‐Ala‐Na** in water/THF (80/20, v/v) optimized by DFT calculations. The structures are represented by capped‐stick models except for the **TPEBe‐I** molecules, which are highlighted as space‐filling ones. The carbon atoms of the helical main chain are highlighted in purple. j) Enlarged model corresponding to the areas indicated by the green square in (i). Noncovalent interactions are indicated by green dashed lines in (j). The **TPEBe‐I** molecule is represented by the capped‐stick model.

Interestingly, for the **TPEBe‐I**/**PPE‐Ala‐Na** complex at the ratio of 1/36, the obvious emission color changes can be observed upon increasing the amount of THF (Figure 4a,b). As the THF fraction increases from 1 vol% to 20 vol%, the emission intensity of the dye at 588 nm significantly decreases. Meanwhile, a new emission peak at 634 nm appears, changing the emission color from yellow to red. The fluorescence changes are the same as those produced by direct titration of 20 vol% THF into the initial solution of **TPEBe‐I**/**PPE‐Ala‐Na** complex (Figure S26). In other words, the fluorescence of static solutions at different solvent volume ratios can be used to monitor the solvent‐triggered conformational transitions. When the THF fraction increases to 50 vol%, the emission wavelength takes a further red shift from 634 nm to 657 nm, and a pale red emission is observed. By the further addition of 90 vol% THF, the solution is almost nonemissive with a weak peak at 674 nm. Notably, the pure foldamer **PPE‐Ala‐Na** is always nonemissive in water/THF mixtures at a low concentration of 36 µM (Figure S27). Therefore, the emission color and emission spectral changes of the dye are ascribed to the local environmental variations such as solvent polarity and/or conformational transitions in the foldamer.

The CD and UV−vis spectra of **PPE‐Ala‐Na** complexed with **TPEBe‐I** in water/THF mixtures demonstrate that the foldamer shows obvious conformational changes (Figures 4c–e and S28). By increasing the THF fraction to 20 vol%, the first Cotton effect sign changes from negative to positive, accompanied by a hyperchromic effect at 285 nm due to the weakened π–π stacking in the main chain^[^ ^48^ ^]^ and the appearance of a weak shoulder absorption at 306 nm corresponding to the *transoid* conformer of *m*‐phenylene ethynylene units. The random‐coil content (*X*_C_) is estimated to be only 6.3% (Figure 4f) (for the calculation method, see Section 4 in the Supporting Information). In other words, a helix inversion occurs when increasing the THF fraction: the original (*M*)‐handed foldamer now mainly adopts a slightly extended right (*P*)‐handed helical conformation. Interestingly, the THF‐triggered helix inversion is a reversible behavior (Figure S29). Such a helix inversion is attributed to the solvent‐triggered orientation change of some pendant carbonyl groups from antiperiplanar (*anti*) to synperiplanar (*syn*), without the aid of the dye (Figures S30–S35).

Furthermore, the MD simulation of the **TPEBe‐I**/**PPE‐Ala‐Na** complex in water/THF (80/20, v/v) reveals that the dye is docked on the surface of the (*P*)‐handed foldamer helix after equilibration (Movie S2 and Figure S36). On the one hand, the presence of THF can markedly increase the solvation of the dye. Accordingly, the hydrophobic effect, a driving force for the groove binding, is destroyed, as evidenced by the fact that the dye is surrounded by several THF molecules (Figure S36). On the other hand, unlike the *anti*‐oriented pendants, the *syn*‐oriented ones with a higher steric hindrance^[^ ^49^ ^]^ are unfavorable for the entrapment of the dye into the groove (Figure 4g). The DFT calculations demonstrate that there exist the COO^−^⋯N^+^, C─H⋯π, C─H⋯S, C─H⋯N, and C─H⋯O interactions in the complex (Figure S37 and Table S2). Compared to the **TPEBe‐I**/**PPE‐Ala‐Na** complex in water/THF (99/1, v/v) (Figure 3f,g), the intramolecular motions of the dye in the complex in water/THF (80/20, v/v) are less inhibited, as evidenced by its fewer number of noncovalent interactions and lower binding energy (Figure 4i,j). In these two complexes, the conformational difference of the dye does not have much influence on the HOMO−LUMO energy gap (from 2.481 eV to 2.544 eV in Figure S38). However, the dye on the foldamer surface is exposed to the solvent molecules, significantly promoting ICT.^[^ ^50^ ^]^ As a result, the dye shows a decreased and redshifted emission.

As the THF fraction increases to 50 vol%, the absorption at 306 nm exhibits a significant hyperchromic effect, indicative of a partial helix‐coil transition (*X*_C_ = 77%). In other words, the initial foldamer helix with 24 turns now possesses ~6 turns, so only one dye can be located on the foldamer surface, whereas the other three dyes are in an isolated state (Figure 4h). Thus, a further emission wavelength redshift occurs and a pale red emission is observed (Figure 4a,b). By the further addition of 90 vol% THF, the disappearance of the absorption at 306 nm and the decreased Cotton effect intensity indicate that the foldamer refolds into a (*P*)‐handed helix with a helical sense bias (Figure 4h). However, the four dyes are completely isolated because the driving force for the complexation of the dyes with the foldamer disappears due to the presence of a large amount of THF. Finally, a longer emission wavelength occurs at 674 nm, along with the appearance of the emission peak of the LE state at 475 nm.^[^ ^38^ ^]^ This longer wavelength is in good agreement with that of 676 nm for the pure dye in pure THF (Figure 2b), and a nonemissive solution is obtained.

### Visualization of Helix Inversion in PPE‐Ala‐Na in Water/ACN and Water/DMSO Mixtures

Besides THF, other solvents such as ACN and DMSO with higher polarities can also trigger **PPE‐Ala‐Na** to undergo an extended helix inversion (Figures S39–S44). As expected, the helix inversion in the foldamer can be visually observed with the aid of the dye in water/ACN and water/DMSO mixtures (Figures 5 and S45–S51).

**Figure 5:**
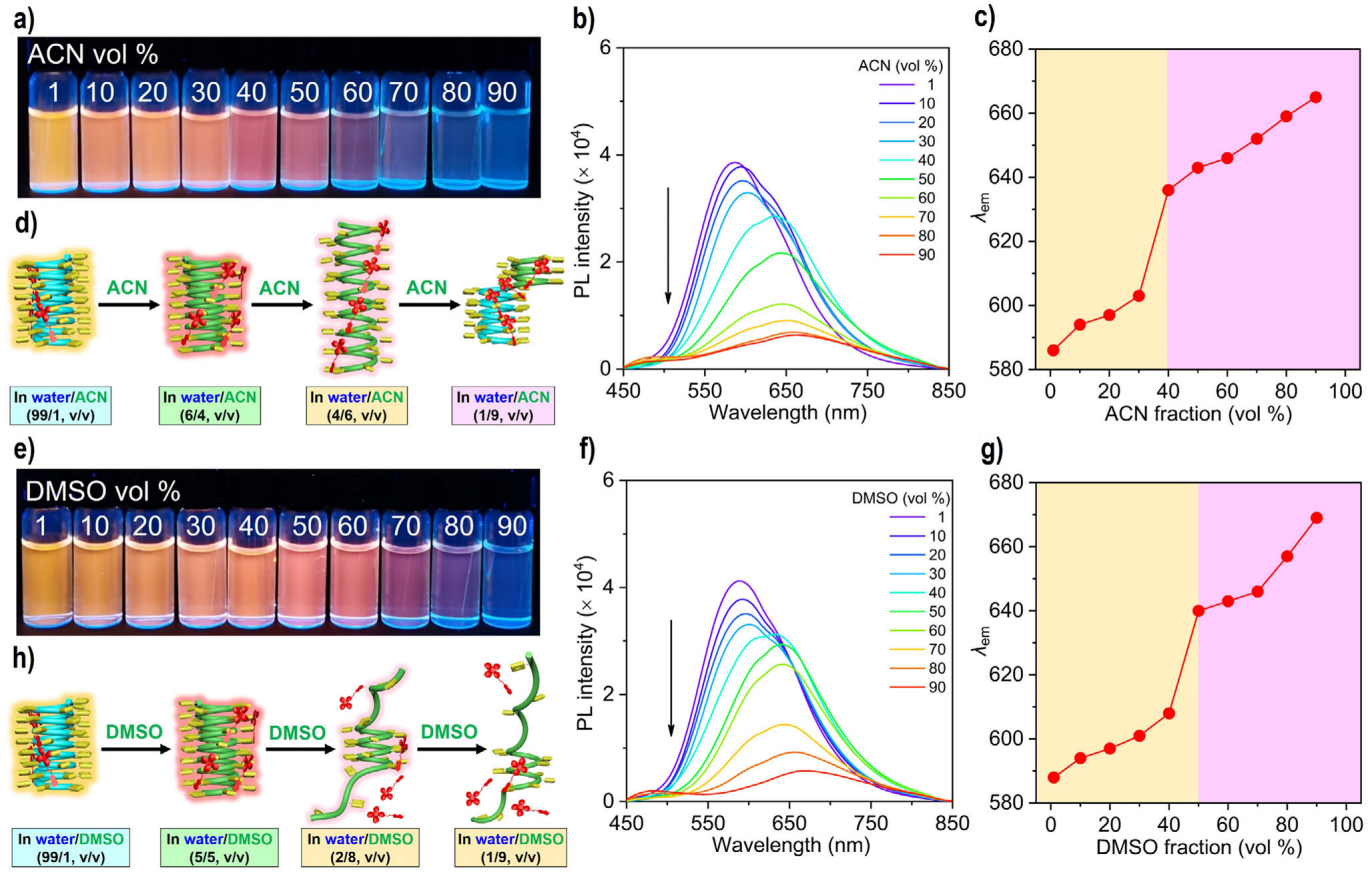
Fluorescence photographs of **TPEBe‐I** complexed with **PPE‐Ala‐Na** in a) water/ACN and e) water/DMSO mixtures under 365 nm UV irradiation. b) and f) Fluorescence spectra of **TPEBe‐I** with **PPE‐Ala‐Na** in (b) water/ACN and (f) water/DMSO mixtures with different organic solvent fractions. [**TPEBe‐I**] = 1 µM; [**PPE‐Ala‐Na**] = 36 µM; excitation wavelength (*λ*_ex_): 420 nm. c) and g) Plots of the emission wavelength (*λ*_em_) of **TPEBe‐I** with **PPE‐Ala‐Na** versus (c) ACN and (g) DMSO fraction. d) and h) Illustrative conformational transitions of **PPE‐Ala‐Na** in the presence of **TPEBe‐I** in (d) water/ACN and (h) water/DMSO mixtures.

In water/ACN mixtures, the emission color changes from yellow to red at 40 vol% ACN (Figure 5a). The emission peak at 586 nm corresponding to the groove binding disappears, but the emission peak at 636 nm appears owing to the surface binding (Figure 5b,c). Meanwhile, the initial (*M*)‐handed helix is transitioned into a slightly extended (*P*)‐handed one (Figures 5d and S45). Obviously, this transition can be visualized directly by the naked eye through the emission color change. At 60 vol% ACN, a pale red emission is observed because the dyes are weakly restricted on the surface of the foldamer with a more extended (*P*)‐handed helix. In the presence of 90 vol% ACN, a nonemissive solution is obtained. The emission wavelength is 665 nm, shorter than that of the pure dye at 690 nm in pure ACN (Figures 2b and 5b,c). Although the dyes are still located on the foldamer helix, the RIM is further decreased for the dyes at such a high ACN fraction.

The same emission color change from yellow to red is observed when the foldamer undergoes a slightly extended helix inversion at 50 vol% DMSO (Figure 5e–h). As the DMSO fraction is increased to 80 vol%, a pale red emission is observed because the foldamer helix with 8 turns (Figure S51) can only bind with one dye on the surface due to a partial helix‐coil transition. At a high DMSO fraction of 90 vol%, the solution is nonemissive in the strong polar circumstance, although the dye is still on the foldamer surface.

## Conclusion

In summary, we report a simple, rapid, and robust method to detect the solvent‐triggered helix inversion in a water‐soluble poly(*m*‐phenylene ethynylene)‐based foldamer (**PPE‐Ala‐Na**) by **TPEBe‐I** as a visualizing agent. The binding mechanisms of **PPE‐Ala‐Na** and **TPEBe‐I** are investigated in detail. In the aqueous solution, the dyes are inserted into the foldamer groove via electrostatic interaction and hydrophobic effect, which induces a yellow emission. The helix inversion of the foldamer can be easily identified by the emission color change from yellow to red because the binding position of the dyes shifts to the foldamer surface. Compared to the traditional detection techniques, this visualization method does not require troublesome and time‐consuming sample preparation procedures, or complicated chemical treatment. The present investigation is crucial in deepening our understanding of the conformational transition processes in biomacromolecules and promoting the development of fluorescent probes for sensing applications. Besides, artificial helical foldamers and polymers usually show significant variations in helical conformation,^[^ ^51^, ^52^ ^]^ photophysical properties,^[^ ^53^, ^54^ ^]^ chiral functions,^[^ ^55^, ^56^ ^]^ etc., by slightly altering the structures of the main chain and/or side groups.^[^ ^57^ ^]^ Different from the previously reported poly(*m*‐phenylene diethynylene)‐based foldamer^[^ ^36^ ^]^ that undergoes solvent‐driven helix‐coil transition/helix extension, the helix inversion characteristic in **PPE‐Ala‐Na** enables the foldamer to be a more potent chiral material in switchable chiral separation^[^ ^58^, ^59^ ^]^ as well as enantioselective catalysis.^[^ ^60^ ^]^ In this regard, the dye as a fluorescence probe can be integrated into a foldamer‐containing reaction vessel, allowing for continuous, real‐time reading of helicity changes to obtain the desired enantiomers, without the use of high‐performance liquid chromatography (HPLC).

## Conflict of Interests

The authors declare no conflict of interest.

## Supporting information



Supporting Information

Supporting Information

Supporting Information

## Data Availability

The data that support the findings of this study are available in the Supporting Information of this article.

## References

[1] Y. He , J. Zhang , C. Ma , J. Liu , J. Guo , T. Han , R. Hu , B. S. Li , B. Z. Tang , Aggregate 2024, 5, e642.

[2] Y. Li , R. Tian , H. Shi , J. Xu , T. Wang , J. Liu , Aggregate 2023, 4, e317.

[3] E. Yashima , K. Maeda , H. Iida , Y. Furusho , K. Nagai , Chem. Rev. 2009, 109, 6102–6211, 10.1021/cr900162q. 19905011

[4] Y. Qiu , X. Wei , J. W. Y. Lam , Z. Qiu , B. Z. Tang , ACS Nano 2025, 19, 229–280, 10.1021/acsnano.4c14797. 39754598

[5] H. Miyake , H. Tsukube , Chem. Soc. Rev. 2012, 41, 6977, 10.1039/c2cs35192g. 22850749

[6] S. Wang , Y. Long , J. Wang , Y. Ge , P. Guo , Y. Liu , T. Tian , X. Zhou , J. Am. Chem. Soc. 2014, 136, 56–59, 10.1021/ja4107012. 24364741

[7] S. C. Ha , K. Lowenhaupt , A. Rich , Y.‐G. Kim , K. K. Kim , Nature 2005, 437, 1183–1186, 10.1038/nature04088. 16237447

[8] J. Zhao , A. Bacolla , G. Wang , K. M. Vasquez , Cell. Mol. Life Sci. 2010, 67, 43–62, 10.1007/s00018-009-0131-2. 19727556 PMC3017512

[9] E. Yashima , N. Ousaka , D. Taura , K. Shimomura , T. Ikai , K. Maeda , Chem. Rev. 2016, 116, 13752–13990, 10.1021/acs.chemrev.6b00354. 27754649

[10] L. Xu , C. Wang , Y.‐X. Li , X.‐H. Xu , L. Zhou , N. Liu , Z.‐Q. Wu , Angew. Chem. Int. Ed. 2020, 59, 2–10.

[11] L. Zhou , X.‐H. Xu , Z.‐Q. Jiang , L. Xu , B.‐F. Chu , N. Liu , Z.‐Q. Wu , Angew. Chem. Int. Ed. 2021, 60, 806–812, 10.1002/anie.202011661. 33006185

[12] X. Guan , S. Wang , G. Shi , J. Zhang , X. Wan , Macromolecules 2021, 54, 4592–4600, 10.1021/acs.macromol.1c00538.

[13] S. Wang , J. Chen , X. Feng , G. Shi , J. Zhang , X. Wan , Macromolecules 2017, 50, 4610–4615, 10.1021/acs.macromol.7b01028.

[14] L. Zhang , Q. Jin , M. Liu , Chem. Asian J. 2016, 11, 2642–2649, 10.1002/asia.201600441. 27258582

[15] M. Banno , T. Yamaguchi , K. Nagai , C. Kaiser , S. Hecht , E. Yashima , J. Am. Chem. Soc. 2012, 134, 8718–8728, 10.1021/ja303204m. 22540863

[16] K. Maeda , L. Hong , T. Nishihara , Y. Nakanishi , Y. Miyauchi , R. Kitaura , N. Ousaka , E. Yashima , H. Ito , K. Itami , J. Am. Chem. Soc. 2016, 138, 11001–11008, 10.1021/jacs.6b05582. 27486790

[17] H. Abe , K. Okada , H. Makida , M. Inouye , Org. Biomol. Chem. 2012, 10, 6930, 10.1039/c2ob25816a. 22825466

[18] S. Pramanik , B. Kauffmann , S. Hecht , Y. Ferrand , I. Huc , Chem. Commun. 2021, 57, 93–96, 10.1039/D0CC06484J. 33332504

[19] P. Zhang , L. Zhang , Z.‐K. Wang , Y.‐C. Zhang , R. Guo , H. Wang , D.‐W. Zhang , Z.‐T. Li , Chem. Asian J. 2016, 11, 1725–1730, 10.1002/asia.201600289. 27027979

[20] H. Makida , H. Abe , M. Inouye , Org. Biomol. Chem. 2015, 13, 1700–1707, 10.1039/C4OB02129K. 25473810

[21] R. M. Meudtner , S. Hecht , Angew. Chem. Int. Ed. 2008, 47, 4926–4930, 10.1002/anie.200800796. 18496819

[22] J.‐m. Suk , V. R. Naidu , X. Liu , M. S. Lah , K.‐S. Jeong , J. Am. Chem. Soc. 2011, 133, 13938–13941, 10.1021/ja206546b. 21848269

[23] S. Takashima , H. Abe , M. Inouye , Chem. Commun. 2011, 47, 7455, 10.1039/c1cc12358k. 21629899

[24] L. Liu , N. Ousaka , M. Horie , F. Mamiya , E. Yashima , Chem. Commun. 2016, 52, 11752–11755, 10.1039/C6CC05753E. 27709199

[25] S. Cao , C. Sun , J. Wang , Q. Jiang , Y. Qiu , H. Wang , Y. Liao , X. Xie , Macromol. Rapid Commun. 2022, 43, e2200238, 10.1002/marc.202200238. 35510985

[26] S. Leiras , F. Freire , J. M. Seco , E. Quiñoá , R. Riguera , Chem. Sci. 2013, 4, 2735, 10.1039/c3sc50835h. PMC5433055 28553473

[27] Y. Hong , J. W. Y. Lam , B. Z. Tang , Chem. Commun. 2009, 4332, 10.1039/b904665h. 19597589

[28] W.‐J. Wang , Z.‐Y. Xin , X. Su , L. Hao , Z. Qiu , K. Li , Y. Luo , X.‐M. Cai , J. Zhang , P. Alam , J. Feng , S. Wang , Z. Zhao , B. Z. Tang , ACS Nano 2025, 19, 281–306, 10.1021/acsnano.4c14887. 39745533

[29] Y. Cheng , J. Wang , Z. Qiu , X. Zheng , N. L. C. Leung , J. W. Y. Lam , B. Z. Tang , Adv. Mater. 2017, 29, 1703900, 10.1002/adma.201703900. 29044736

[30] Z. Hu , H. Zhang , H. Liu , J. Li , X. Ji , B. Z. Tang , SmartMat 2023, 5, e1184.

[31] Y. Jiang , Y. Cheng , S. Liu , H. Zhang , X. Zheng , M. Chen , M. Khorloo , H. Xiang , B. Z. Tang , M. Zhu , Natl. Sci. Rev. 2021, 8, nwaa135, 10.1093/nsr/nwaa135. 34691610 PMC8288334

[32] Y. Yang , X. Zhou , X. Ji , W. Liu , Q. Li , C. Zhu , X. Li , S. Liu , X. Lu , J. Qu , Adv. Funct. Mater. 2025, 35, 2416776, 10.1002/adfm.202416776.

[33] Y. Hu , L. Barbier , Z. Li , X. Ji , H. L. Blay , D. Hourdet , N. Sanson , J. W. Y. Lam , A. Marcellan , B. Z. Tang , Adv. Mater. 2021, 33, 2101500, 10.1002/adma.202101500. 34350646

[34] L. Zhou , L. Zheng , X. Yu , M. Gao , C. Xu , Y. Ge , T. Bai , J. Wen , Y. Cheng , M. Zhu , Aggregate 2023, 4, e338.

[35] K. Xue , C. Wang , J. Wang , S. Lv , B. Hao , C. Zhu , B. Z. Tang , J. Am. Chem. Soc. 2021, 143, 14147–14157, 10.1021/jacs.1c04597. 34288685

[36] C. Sun , Q. Jiang , H. Ruan , T. Wang , Y. Qiu , H. Wang , Y. Liao , X. Xie , Macromolecules 2023, 56, 2809–2817, 10.1021/acs.macromol.2c02566.

[37] N. Zhao , J. W. Y. Lam , H. H. Y. Sung , H. M. Su , I. D. Williams , K. S. Wong , B. Z. Tang , Chem. Eur. J. 2014, 20, 133–138, 10.1002/chem.201303251. 24375854

[38] R. Hu , E. Large , A. Aguilar‐Aguilar , J. Liu , J. W. Y. Lam , H. H. Y. Sung , I. D. Williams , Y. Zhong , K. S. Wong , E. Peña‐Cabrera , B. Z. Tang , J. Phys. Chem. C 2009, 113, 15845–15853, 10.1021/jp902962h.

[39] J. Chen , C. C. W. Law , J. W. Y. Lam , Y. Dong , S. M. F. Lo , I. D. Williams , D. Zhu , B. Z. Tang , Chem. Mater. 2003, 15, 1535–1546, 10.1021/cm021715z.

[40] Y. Qiu , H. Hu , D. Zhao , J. Wang , H. Wang , Q. Wang , H. Peng , Y. Liao , X. Xie , Polymer 2019, 170, 7–15, 10.1016/j.polymer.2019.02.063.

[41] C. Tan , M. R. Pinto , M. E. Kose , I. Ghiviriga , K. S. Schanze , Adv. Mater. 2004, 16, 1208–1212, 10.1002/adma.200306711.

[42] J. C. Nelson , J. G. Saven , J. S. Moore , P. G. Wolynes , Science 1997, 277, 1793–1796, 10.1126/science.277.5333.1793. 9295264

[43] R. B. Prince , J. G. Saven , P. G. Wolynes , J. S. Moore , J. Am. Chem. Soc. 1999, 121, 3114–3121, 10.1021/ja983995i.

[44] W.‐Z. Sun , W.‐J. Zhang , J.‐T. Xu , D.‐Q. Liu , B.‐Q. Ou , L. Chen , J.‐W. Ye , X.‐M. Chen , Aggregate 2025, 6, e721, 10.1002/agt2.721.

[45] Y. Hong , M. Häuβler , J. W. Y. Lam , Z. Li , K. K. Sin , Y. Dong , H. Tong , J. Liu , A. Qin , R. Renneberg , B. Z. Tang , Chem. Eur. J. 2008, 14, 6428–6437, 10.1002/chem.200701723. 18512826

[46] T. Lu , Q. Chen , J. Comput. Chem. 2022, 43, 539–555, 10.1002/jcc.26812. 35108407

[47] P. L. A. Popelier , Chemical Bond: Fundamental Aspects of Chemical Bonding 2014, Wiley‐VCH Verlag GmbH & Co. KGaA, Weinheim, Germany, pp. 271–308. 10.1002/9783527664696.

[48] M. Waki , H. Abe , M. Inouye , Angew. Chem. Int. Ed. 2007, 46, 3059–3061, 10.1002/anie.200604176. 17352449

[49] I. Louzao , J. M. Seco , E. Quiñoá , R. Riguera , Angew. Chem. Int. Ed. 2010, 49, 1430–1433; 10.1002/anie.200905222 20084648

[50] Y. Zhang , J. Wang , P. Jia , X. Yu , H. Liu , X. Liu , N. Zhao , B. Huang , Org. Biomol. Chem. 2010, 8, 4582, 10.1039/c0ob00030b. 20714660

[51] S.‐i. Sakurai , K. Okoshi , J. Kumaki , E. Yashima , Angew. Chem. Int. Ed. 2006, 45, 1245–1248, 10.1002/anie.200503136. 16411264

[52] S.‐i. Sakurai , S. Ohsawa , K. Nagai , K. Okoshi , J. Kumaki , E. Yashima , Angew. Chem. Int. Ed. 2007, 46, 7605–7608, 10.1002/anie.200701546. 17763511

[53] H. Sogawa , Y. Miyagi , M. Shiotsuki , F. Sanda , Macromolecules 2013, 46, 8896–8904, 10.1021/ma401730e.

[54] Y.‐Q. Huang , Y.‐Y. Zhong , R. Zhang , Y.‐K. Zhao , X.‐F. Liu , G.‐W. Zhang , Q.‐L. Fan , L.‐H. Wang , W. Huang , Polymer 2016, 102, 143–152, 10.1016/j.polymer.2016.08.044.

[55] W. Zheng , T. Ikai , E. Yashima , Angew. Chem. Int. Ed. 2021, 60, 11294–11299, 10.1002/anie.202102885. 33709523

[56] W. Zheng , K. Oki , R. Saha , Y. Hijikata , E. Yashima , T. Ikai , Angew. Chem. Int. Ed. 2023, 62, e202218297. 10.1002/anie.202218297 36680515

[57] E. Yashima , K. Maeda , Bull. Chem. Soc. Jpn. 2021, 94, 2637–2661, 10.1246/bcsj.20210282.

[58] K. Shimomura , T. Ikai , S. Kanoh , E. Yashima , K. Maeda , Org. Nat. Chem. 2014, 6, 429–434, 10.1038/nchem.1916. 24755595

[59] D. Hirose , A. Isobe , E. Quiñoá , F. Freire , K. Maeda , J. Am. Chem. Soc. 2019, 141, 8592–8598, 10.1021/jacs.9b03177. 31062976

[60] T. Ikai , M. Ando , M. Ito , R. Ishidate , N. Suzuki , K. Maeda , E. Yashima , J. Am. Chem. Soc. 2021, 143, 12725–12735, 10.1021/jacs.1c05620. 34347469

