# Supplementary material for: Fluorescence Visualization of Helix Inversion in Biomimic Polymeric Foldamer
Source: Angew Chem Int Ed Engl. 2025 Oct 29;64(52):e12834. doi: 10.1002/anie.202512834 (PMC12723458; doi:10.1002/anie.202512834)
Supplement: Supplementary file 1 — Supporting Information [file ANIE-64-e12834-s002.docx]

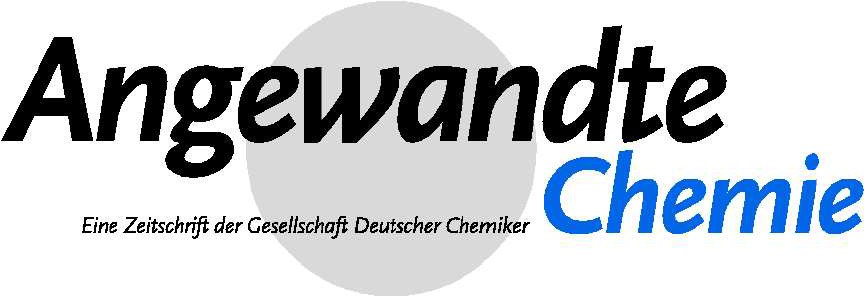


**Supporting Information**

**Fluorescence Visualization of Helix Inversion in Biomimic Polymeric Foldamer**

*Yuan Qiu, Zonghang Liu, Chenchen Sun, Xilong Wei, Ryan T. K. Kwok, Yonggui Liao,* Jianwei Sun, Jacky W. Y. Lam,* Zijie Qiu,* Xiaolin Xie, Ben Zhong Tang**

**Table of Contents**

1. Materials and Characterization
2. Synthetic Procedures
3. CD, UV−vis Absorption, and Fluorescence Measurements
4. Determination of the Content of Random-coil Conformation (*X*_C_)
5. Molecular Dynamics Simulations of **PPE-Ala-Na**
6. Calculated CD and UV−vis Spectra of (*M*)-Handed Helical **PPE-Ala-Na**
7. Molecular Modeling and Calculations of **PPE-Ala-Na**/**TPEBe-I** Complexes
8. Luminescent Behavior of **TPEBe-I** in CHCl_3_/Toluene Mixtures
9. Structure Characterization of Monomers and Polymers
10. Conformation Analysis of **PPE-Ala-Na** in Aqueous Solution
11. Binding Behavior of **TPEBe-I** with **PPE-Ala-Na** in Water/THF (99/1, v/v)
12. Molecular Modeling and Calculations of **PPE-Ala-Na**/**TPEBe-I** Complex in Water/THF (99/1, v/v)
13. Conformational Transitions of **PPE-Ala-Na** with and without **TPEBe-I** in Water/THF Mixtures
14. Molecular Modeling and Calculations of **PPE-Ala-Na**/**TPEBe-I** Complex in Water/THF (80/20, v/v)
15. Conformational Transitions of **PPE-Ala-Na** in Water/ACN and Water/DMSO Mixtures
16. Binding Behavior of **TPEBe-I** with **PPE-Ala-Na** in Water/ACN (99/1, v/v) and Water/DMSO (99/1, v/v)
17. Conformational Transitions of **PPE-Ala-Na** with **TPEBe-I** in Water/THF and Water/DMSO Mixtures
18. Captions for Supporting Movies
19. Supporting References

**1.** **Materials and Characterization**

**Materials.** Tetrakis(triphenylphosphine)palladium (Pd(PPh_3_)_4_), copper(I) iodide (CuI), 4-(4,6-dimethoxy-1,3,5-triazin-2-yl)-4-methylmorpholinium chloride (DMT-MM) and sodium hydroxide were obtained from Aladdin Reagent Co., Ltd. 3,5-Diiodobenzoic acid was available from Adamas. L-Alanine benzyl ester hydrochloride was purchased from Alfachem Reagent Co., Ltd. Extra-dry tetrahydrofuran (THF) and *N*,*N*-dimethylformamide (DMF) were available from Energy Chemical. Triethylamine (TEA) was dried over calcium hydride and distilled under argon. All solvents used for spectroscopic experiments were of spectroscopic grade. 3,5-Diethynyl-benzoic acid^[1]^ and tetraphenylethene-functionalized benzothiazolium iodide (**TPEBe-I**)^[2]^ were synthesized according to our previous reports.

**Characterization.** ^1^H and ^13^C nuclear magnetic resonance (NMR) spectra were recorded on a Bruker AVANCE-Ⅲ-400 at room temperature. Fourier transform-infrared (FT-IR) spectra were obtained with a Bruker Equinox 55 spectrometer. High-resolution mass spectra were measured on a Bruker Solarix 7.0T FT-ICR mass spectrometer. The number-average molecular weight (*M*_n_) and the polydispersity (*M*_w_/*M*_n_) were determined using a size exclusion chromatography (SEC) apparatus equipped with a JASCO PU-980 Intelligent pump and a JASCO RI-930 Intelligent RI detector at 40 °C. DMF containing lithium chloride (0.01 M) was used as the eluent at a flow rate of 0.5 mL min^−1^. The molecular weight calibration curve was obtained with polystyrene standards. Circular dichroism (CD) spectra were measured on a JASCO J-810 spectropolarimeter. UV−vis absorption spectra were recorded using a Shimadzu UV-2550 spectrophotometer. Fluorescence spectra were obtained on a Shimadzu RF-6000 spectrofluorometer. Transmission electron microscopy (TEM) images were obtained using a FEI Talos F200X with an acceleration voltage of 200 kV. The samples for TEM observations were prepared by dropping the solution onto a carbon-coated copper grid, which was then dried under vacuum at room temperature for 6 h. Dynamic light scattering (DLS) measurements were conducted on a Nano-ZS 90 Zetasizer of Malvern instrument. Wide-angle X-ray scattering (WAXS) measurements were conducted on a CuKα sourced (1.54 Å and 8.04 keV) Xenocs Xeuss 2.0 instrument.

**2.** **Synthetic Procedures**

The synthesis of **Mono-A** and **Mono-B** was carried out according to the synthetic route illustrated in **Scheme S1**.


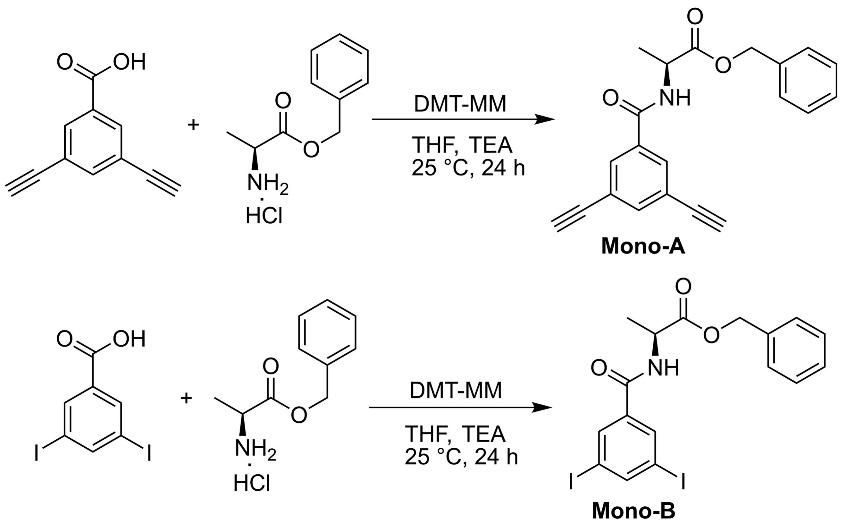


**Scheme S1.** Synthetic route of **Mono-A** and **Mono-B**.

*Synthesis of* ***Mono-A***. To a mixture of 3,5-diethynyl-benzoic acid (0.50 g, 2.94 mmol) and L-alanine benzyl ester hydrochloride (1.29 g, 5.98 mmol) in anhydrous THF (25 mL) were added DMT-MM (1.66 g, 5.98 mmol) and dry TEA (5.5 mL, 39.5 mmol). The mixture was stirred at room temperature for 24 h and the solvent was concentrated by evaporation. The residue was dissolved in ethyl acetate, and washed with 0.5 M HCl, a saturated solution of sodium bicarbonate, and brine and dried over anhydrous sodium sulfate. After filtration, the solvent was removed by evaporation, and the residue was then purified by column chromatography (SiO_2_, petroleum ether/ethyl acetate = 5/1, v/v) to afford Mono-A as a white solid (0.86 g, 86%). ^1^H NMR (400 MHz, DMSO-*d*_6_, TMS, ppm): *δ* = 9.05 (d, -N***H***-, *J* = 6.9 Hz, 1H), 7.99 (d, Ar-***H***, *J* = 1.6 Hz, 2H), 7.75 (t, Ar-***H***, *J* = 1.6 Hz, 1H), 7.40-7.30 (m, Ar-***H***, 5H), 5.15 (d, -O-C***H***_2_-, *J* = 1.9 Hz, 2H), 4.55-4.51 (m, -NH-C***H***-, 1H), 4.42 (s, -C≡C***H***, 2H), 1.43 (d, -CH-C***H***_3_, *J* = 7.3 Hz, 3H). ^13^C NMR (100 MHz, DMSO-*d*_6_, TMS, ppm): *δ* = 172.8 (-***C***O-(ester)), 164.8 (-***C***O-(amide)), 137.4 (aromatic), 136.5 (aromatic), 135.0 (aromatic), 131.4 (aromatic), 128.8 (aromatic), 128.4 (aromatic), 128.1 (aromatic), 123.0 (aromatic), 82.9 (-***C***≡CH), 82.2 (-C≡***C***H), 66.3 (-O-***C***H_2_-), 49.1 (-***C***H-NH-), 16.8 (-CH-***C***H_3_). IR (KBr, cm^-1^): 3281 (≡C-H), 2106 (C≡C), 1738 (C=O), 1633 (C=O). HRMS (ESI-MS): *m/z* calcd for [M(C_21_H_17_NO_3_) + H]^+^, 332.12812; found 332.12798.

The synthesis of **Mono-B** was conducted in the same way as that of **Mono-A** (**Scheme S1**).

Spectroscopic data of **Mono-B**. Yield: 64%. ^1^H NMR (400 MHz, DMSO-*d*_6_, TMS, ppm): *δ* = 9.02 (d, -N***H***-, *J* = 6.9 Hz, 1H), 8.28 (d, Ar-***H***, *J* = 1.6 Hz, 1H), 8.21 (d, Ar-***H***, *J* = 1.6 Hz, 2H), 7.45-7.29 (m, Ar-***H***, 5H), 5.15 (s, -O-C***H***_2_-, 2H), 4.55-4.48 (m, -NH-C***H***-, 1H), 1.42 (d, -CH-C***H***_3_, *J* = 7.3 Hz, 3H). ^13^C NMR (100 MHz, DMSO-*d*_6_, TMS, ppm): *δ* = 172.5 (-***C***O-(ester)), 164.1 (-***C***O-(amide)), 147.3 (aromatic), 137.4 (aromatic), 136.5 (aromatic), 135.8 (aromatic), 128.9 (aromatic), 128.5 (aromatic), 128.1 (aromatic), 96.4 (aromatic), 66.3 (-O-***C***H_2_-), 49.0 (-***C***H-NH-), 16.9 (-CH-***C***H_3_). IR (KBr, cm^-1^): 1733 (C=O), 1630 (C=O), 578 (C-I). HRMS (ESI-MS): *m/z* calcd for [M(C_17_H_15_I_2_NO_3_) + H]^+^, 535.92128; found 535.92141.

*Synthesis of* ***PPE-Ala-Bn***. Under an argon atmosphere, to a mixture of Pd(PPh_3_)_4_ (34 mg, 29.4 μmol) and CuI (8.6 mg, 45.2 μmol) were added a solution of **Mono-A** (0.50 g, 1.51 mmol) in DMF (12 mL), a solution of **Mono-B** (0.81 g, 1.51 mmol) in DMF (12 mL) and TEA (6 mL). The mixture was stirred at 40 °C for 48 h. Then, the resultant solution was precipitated into a large amount of methanol. The precipitate was collected by centrifugation, washed with methanol and dried in vacuum to afford **PPE-Ala-Bn** as a yellow solid (0.80 g, 88%). *M*_n_ = 4.4 × 10^4^, *M*_w_/*M*_n_ = 1.43. ^1^H NMR (400 MHz, DMSO-*d*_6_, TMS, ppm): *δ* = 9.11 (d, -N***H***-, *J* = 6.7 Hz, 1H), 8.14 (d, Ar-***H***, *J* = 8.9 Hz, 2H), 8.03 (s, Ar-***H***, 1H), 7.39-7.29 (m, Ar-***H***, 5H), 5.16 (d, -O-C***H***_2_-, *J* = 17.5 Hz, 2H), 4.61-4.54 (m, -NH-C***H***-, 1H), 1.51-1.35 (m, -CH-C***H***_3_, 3H). IR (KBr, cm^−1^): 3417 (N-H), 1741 (C=O), 1641 (C=O).

*Hydrolysis of* ***PPE-Ala-Bn****.* To a solution of **PPE-Ala-Bn** (0.70 g, 2.29 mmol) in DMF (40 mL) was added aqueous sodium hydroxide (11.5 mmol, 40 mL). The resultant mixture was stirred at room temperature for 2 days and dialyzed against deionized water with 5.0 kDa cutoff. After 48 h, deionized water was concentrated by evaporation, and the residue was precipitated into a large amount of acetone. The precipitate was collected by centrifugation, washed with acetone, and dried in vacuum to afford **PPE-Ala-Na** as a light yellow solid (0.40 g, 70%). ^1^H NMR (400 MHz, D_2_O, TMS, ppm): *δ* = 8.72-7.84 (brs, Ar-***H***, 3H), 4.21 (brs, -C***H***-CH_3_, 1H), 1.32 (brs, -CH-C***H***_3_, 3H). IR (KBr, cm^−1^): 3401 (N-H), 1646 (C=O), 1593 (C=O).

**3.** **CD, UV−vis Absorption, and Fluorescence Measurements**

CD, UV−vis absorption and fluorescence spectra were measured in a 10 mm quartz cell at room temperature. The concentration of **PPE-Ala-Na** was calculated based on its monomeric unit.

**4.** **Determination of the Content of Random-coil Conformation (*X*_C_)**

To quantitatively elucidate the conformational transitions of **PPE-Ala-Na**, we calculated the random-coil content (*X*_C_) based on (*R*_H_ − *R*)/(*R*_H_ − *R*_C_),^[3]^ where *R*_H_ was the molar absorption ratio of *ε*_306_/*ε*_285_ for **PPE-Ala-Na** with a folded helical conformation, *R*_C_ was the ratio for **PPE-Ala-Na** with a random-coil conformation, and *R* is the *ε*_306_/*ε*_285_ ratio for **PPE-Ala-Na** with different volume fractions of organic solvent. Specifically, when poly(*m*-phenylene ethynylene)-based foldamers featured a random-coil conformation, an molar absorption ratio was usually determined to be ~ 1.^[4-6]^

**5. Molecular Dynamics Simulations of PPE-Ala-Na**

The molecular dynamics (MD) simulations were carried out using the Forcite module of the BIOVIA Materials Studio 2020 (Dassault Systèmes BIOVIA, San Diego, CA, USA). Based on the helical structure with ~ 6 monomer units per turn and the absolute handedness of two poly(*m*-phenylene ethynylene)s, i.e., right (*P*)-handed helical (*S*)-**PPEa** and left (*M*)-handed helical (*R*)-**PPEa**, determined by X-ray diffraction (XRD) and high-resolution atomic force microscopy (AFM), respectively,^[7]^ the polymer models composed of 36 repeating monomer units for (*M*)- and (*P*)-handed helical **PPE-Ala-Na** were constructed using a Polymer Builder module in the Materials Studio Modeling software. The helical senses of **PPE-Ala-Na** with opposite CD signals in water and water/THF (80/20, v/v) (**Figure 4a**) are assigned on the basis of the Cotton effect signs of (*R*)-**PPEa** and (*S*)-**PPEa**, respectively.^[7]^ The MD box was built by means of the usual procedure of the Amorphous Cell module. The box length and angle were *a* = *b* = *c* = 60.0 Å and *α* = *β* = *γ* = 90°, respectively. For the MD simulation, one polymer chain was put in the center of a cubic box filled with solvent molecules. The simulation boxes of (*M*)- and (*P*)-handed helical **PPE-Ala-Na** consisted of 7200 water molecules and 5760 water and 320 THF molecules, respectively. Before the MD simulations, these two systems were quasi-statically relaxed to a local minimum-energy configuration. The relaxations were performed for 10,000 ps under the NVT ensemble (constant number of atoms, volume, and temperature) at 300 K, with a 1.0 fs time step. The simulations in the NPT ensemble (constant number of atoms, pressure, and temperature) were conducted for 10,000 ps at the pressure of 0.1 GPa and at 300 K, with a 1.0 fs time step. The Nosé thermostat and Berendsen barostat were used to control the temperature and pressure, respectively. The COMPASS II (ver. 1.2) forcefield was employed to describe the atomic interactions. The Lennard-Jones interactions between unlike particles are truncated at a cut-off distance of 1.25 nm. The particle-particle particle-mesh (PPPM)^[8]^ method is employed to deal with the long-range electrostatic interactions.

**6.** **Calculated CD and UV−vis** **Spectra of (*M*)-Handed Helical** **PPE-Ala-Na**

The initial structure for the computational study was obtained by intercepting 10 repeating monomer units within the minimized model structure of (*M*)-handed helical **PPE-Ala-Na** based on the MD simulations (Section 5). The theoretical calculations of the resulting oligomer model were performed at the density function theory (DFT) level using the Perdew-Burke-Ernzerhof (PBE) exchange–correlation functional^[9]^ together with Grimme’s D3 correction,^[10]^ as implemented in the CP2K package.^[11]^ Unrestricted Kohn–Sham DFT was used as the electronic structure method in the framework of the Gaussian and plane waves method.^[12]^ The Goedecker–Teter–Hutter (GTH) pseudopotentials^[13]^ and DZVP-MOLOPT-GTH basis sets^[14]^ were utilized to describe the molecules. A plane-wave energy cutoff of 400 Ry was employed. The geometry was optimized using the Broyden–Fletcher–Goldfarb–Shanno (BFGS) algorithm, and the convergence criterion for the forces was set to 10^−5^ bohr/Hartree with relaxed hydrogen and sodium atoms.

The calculations of the optimized oligomer model were further conducted at the DFT level using Gaussian 16 C.01 software.^[15]^ All the hydrogen atoms were relaxed, and other atoms were constrained in geometrical optimization, which was performed using the ωB97X-D functional.^[16]^ The 6-31G* basis sets were applied for all elements. The theoretical CD and UV−vis spectra were calculated by the time-dependent density functional theory (TD-DFT) at the ωB97X-D/6-31G* level of theory, and 100 excited states were computed.

The calculated CD and UV−vis spectra were used to determine the helical sense of **PPE-Ala-Na** in water, through comparison with its experimental CD and UV−vis spectra (**Figure 3c**). The calculated spectral patterns of the (*M*)-handed helical **PPE-Ala-Na** showed a very good fit to its experimental patterns (**Figure S17**).

**7. Molecular Modeling and Calculations of PPE-Ala-Na/TPEBe-I Complexes**

The MD simulations were performed to reveal the binding modes of **TPEBe-I** with the (*M*)-handed and (*P*)-handed helical **PPE-Ala-Na** in water/THF (99/1, v/v) and water/THF (80/20, v/v), respectively, using GROMACS version 2021.3.^[17-20]^ The minimized model structures of the (*M*)-handed and (*P*)-handed helical **PPE-Ala-Na** (36-mer) obtained after structural equilibration (Section 5) were used as the initial polymer models. The initial structures of the **PPE-Ala-Na/TPEBe-I** complexes in which **TPEBe-I** interacted with the negatively charged pendants of the (*M*)-handed and (*P*)-handed helical **PPE-Ala-Na** were constructed by Molclus^[21]^ with the computational back-end of GFN2-xTB.^[22]^ The constructed structures were then optimized by ORCA under the function of B97-3C with mTZVP basis set.^[23]^ The MD box with the length of *a* = *b* = *c* = 60.0 Å and the angle of *α* = *β* = *γ* = 90° was constructed using Packmol.^[24]^ For the MD simulation, the optimized structure of the **PPE-Ala-Na/TPEBe-I** complex was put in the center of a cubic box filled with solvent molecules. The simulation boxes of (*M*)-handed helical **PPE-Ala-Na/TPEBe-I** and (*P*)-handed helical **PPE-Ala-Na/TPEBe-I** consisted of 7128 water and 16 THF molecules and 5760 water and 320 THF molecules, respectively. The atomic interactions were parameterized by the general AMBER force field (GAFF),^[25]^ and the restrained electrostatic potential (RESP2) charge^[26]^ obtained from Multiwfn was applied in the calculations. After the energy minimization, these two systems were pre-balanced in NPT ensemble with Berendsen method for 1 ns. The MD simulations were then carried out for 20,000 ps in the NPT ensemble at pressure of 0.1 GPa and at 300 K with a time step of 1 fs. The temperature and pressure were controlled by the V-rescale thermostat (*τ*_T_ =1 ps) and Parrinello-Rahman method (*τ*_P_ = 2 ps), respectively. The resulting **PPE-Ala-Na/TPEBe-I** complexes were further optimized in ORCA as mentioned above. Taking account for the different solvent environments, the conductor-like polarized continuum model (CPCM)^[27]^ was used for the simulation of solvent effects of H_2_O/THF (99/1, v/v) and H_2_O/THF (80/20, v/v), where the dielectric constants were set to 77 and 64, respectively. The independent gradient model based on Hirshfeld partition (IGMH[6]) analysis was performed to visualize the interactions between **PPE-Ala-Na** and **TPEBe-I** using the Multiwfn 3.8 software.^[28]^ Quantum theory of atoms in molecules (QTAIM[7]) was used to evaluate the intermolecular interactions formed in the **PPE-Ala-Na/TPEBe-I** complexes.

**8. Luminescent Behavior of TPEBe-I in CHCl_3_/Toluene Mixtures**


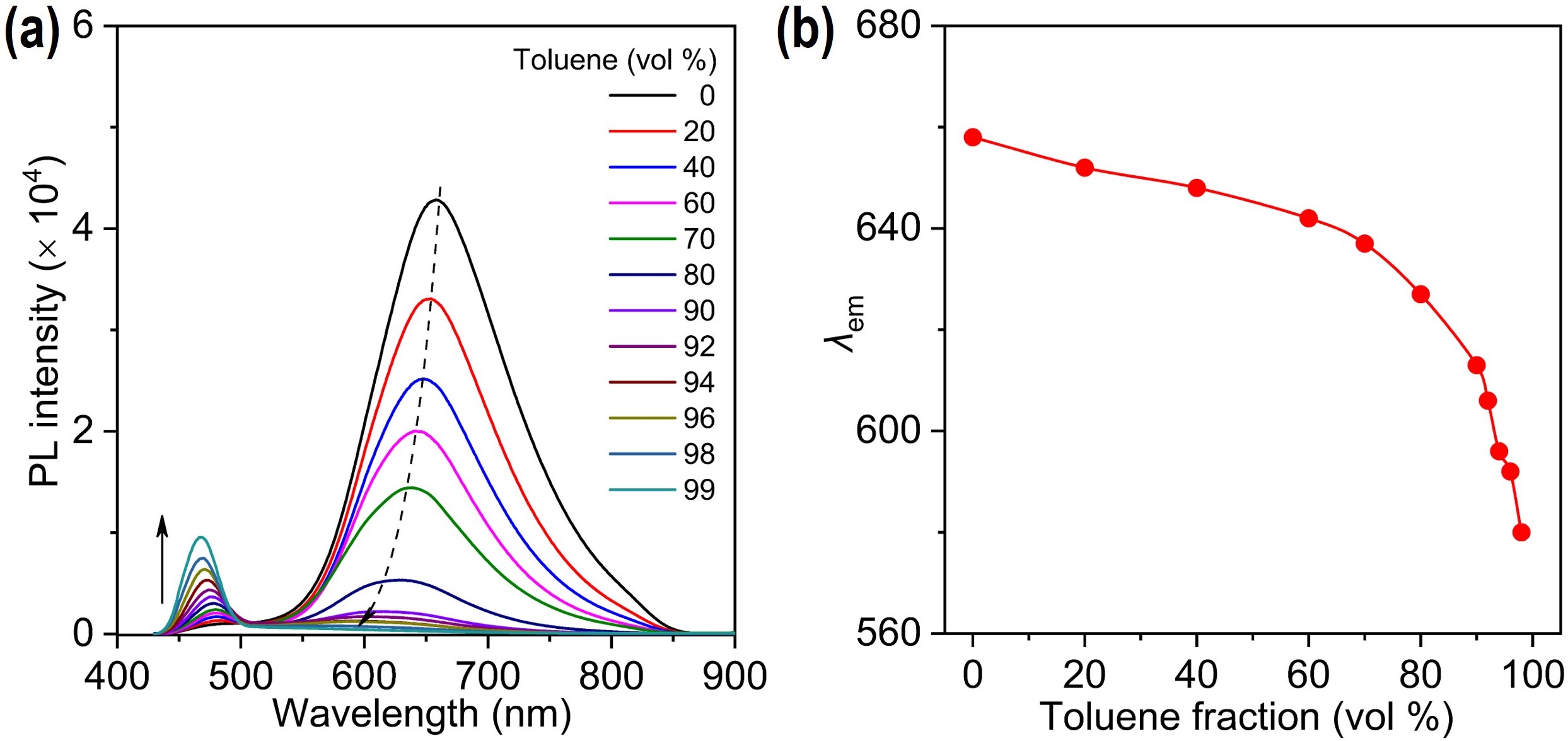


**Figure S1.** Fluorescence spectra of **TPEBe-I** in CHCl_3_/toluene mixtures with different volume fractions of toluene at room temperature. [**TPEBe-I**] = 10 μM; excitation wavelength (*λ*_ex_): 420 nm. (b) Plot of the maximum emission wavelength (*λ*_em_) of **TPEBe-I** versus toluene fraction.

**9. Structure Characterization of Monomers and Polymers**


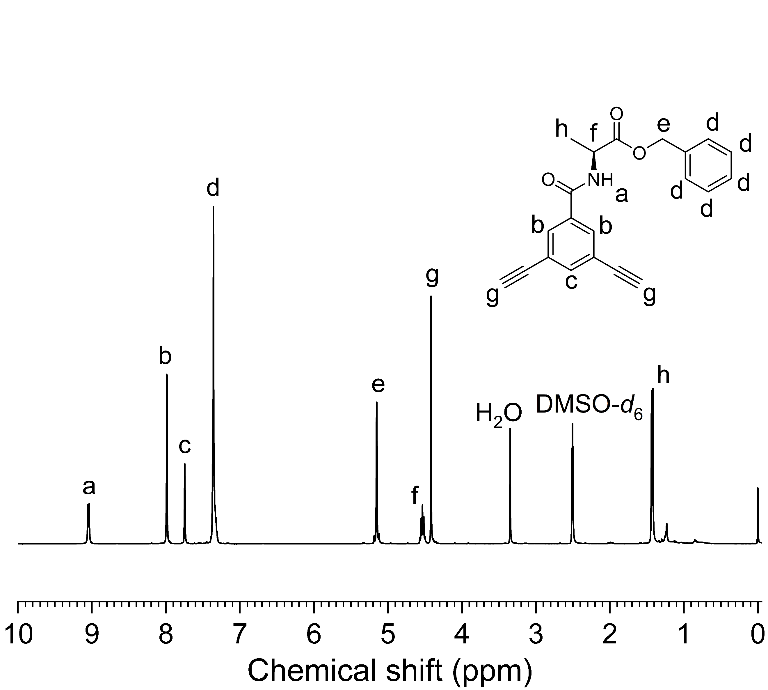


**Figure S2*.*** ^1^H NMR spectrum of **Mono-A** in DMSO-*d*_6_ at room temperature.


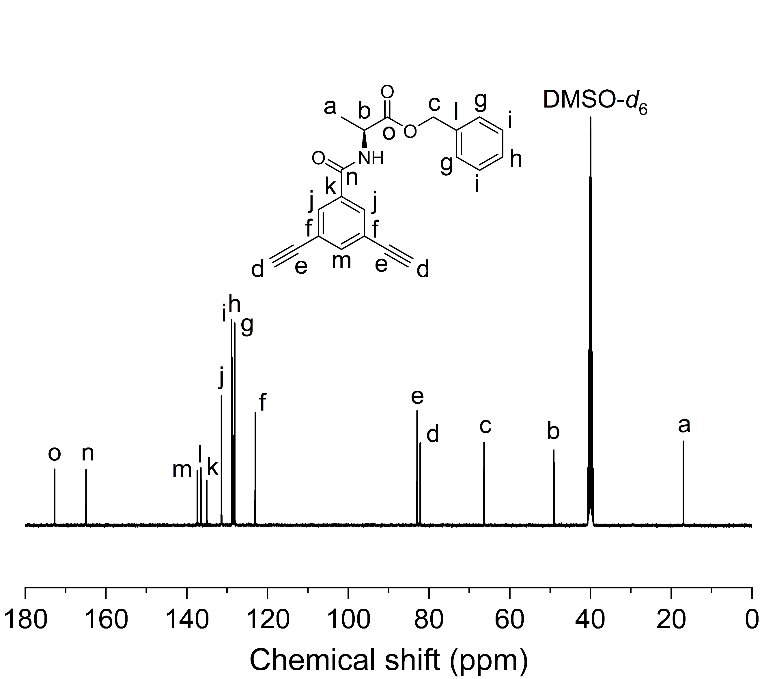


**Figure S3*.*** ^13^C NMR spectrum of **Mono-A** in DMSO-*d*_6_ at room temperature.


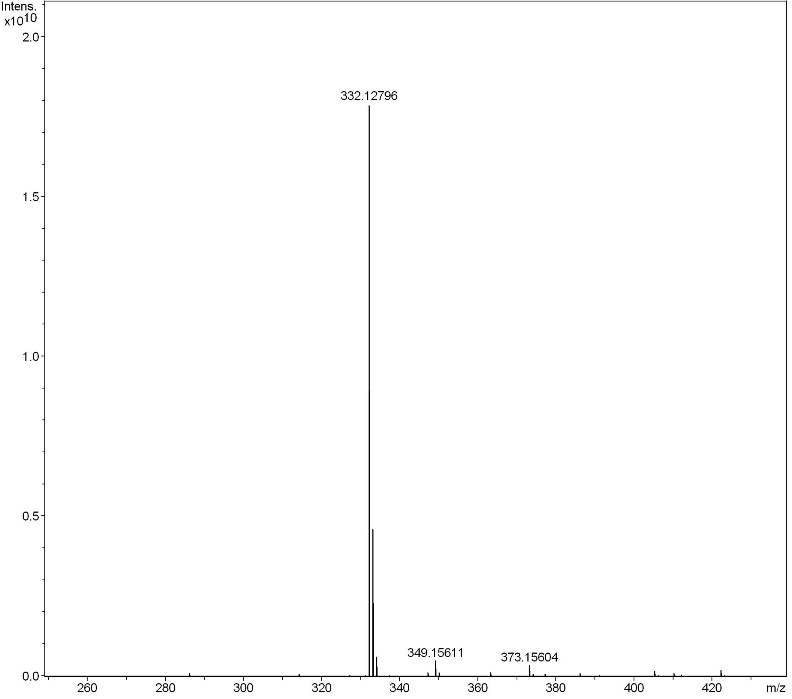


**Figure S4*.*** HRMS spectrum of **Mono-A**.


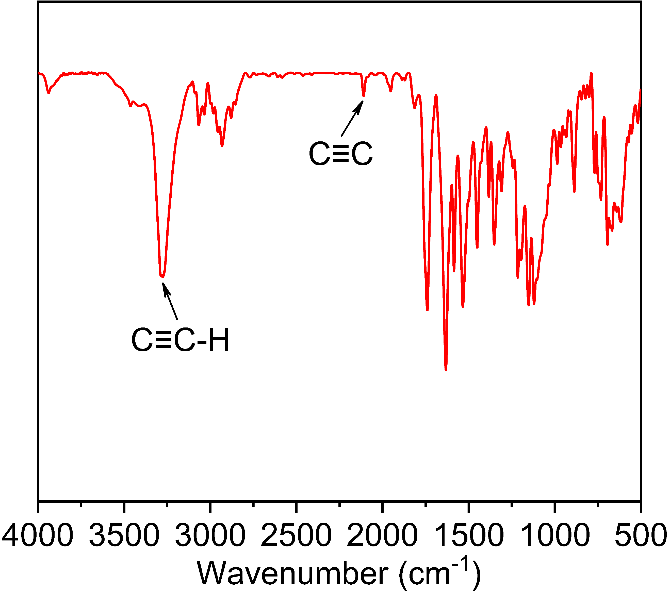


**Figure S5*.*** FTIR spectrum of **Mono-A** measured at room temperature (KBr tablet).


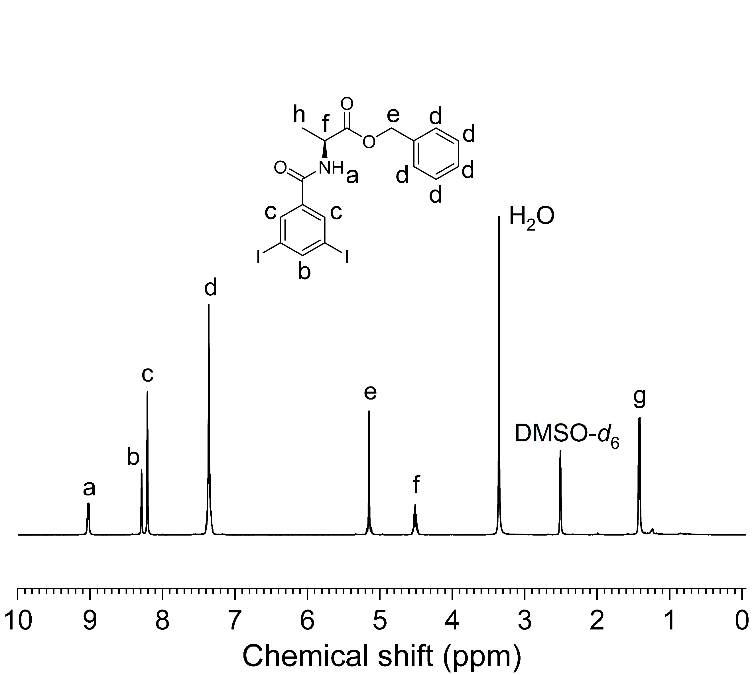


**Figure S6*.*** ^1^H NMR spectrum of **Mono-B** in DMSO-*d*_6_ at room temperature.


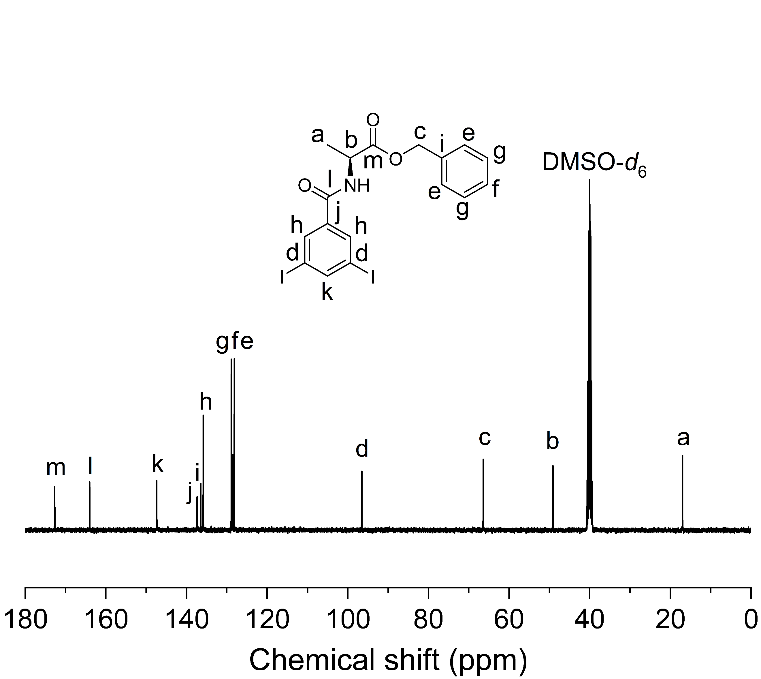


**Figure S7*.*** ^13^C NMR spectrum of **Mono-B** in DMSO-*d*_6_ at room temperature.


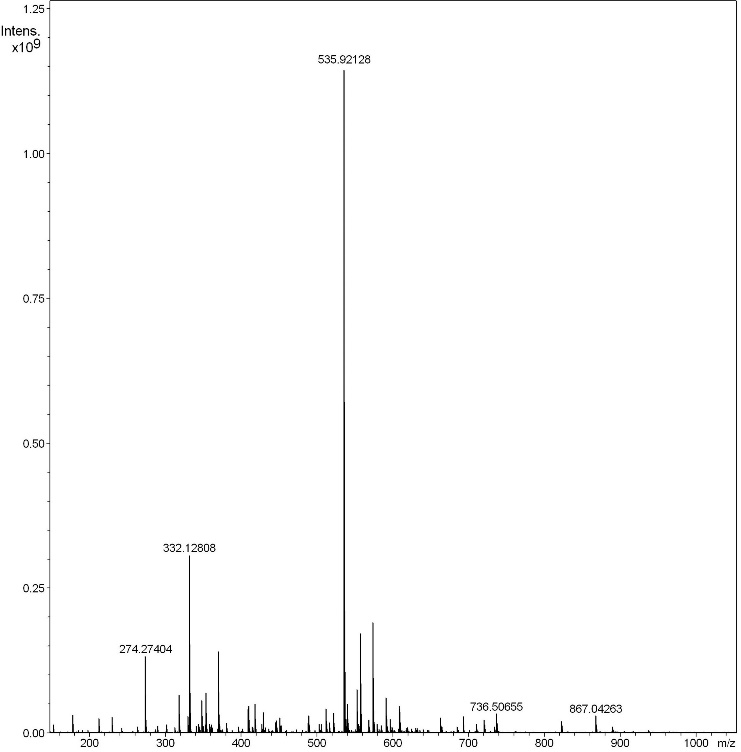


**Figure S8*.*** HRMS spectrum of **Mono-B**.


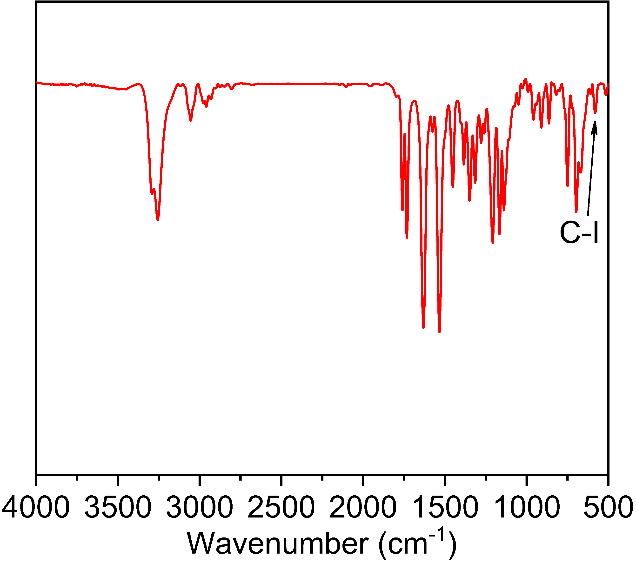


**Figure S9*.*** FTIR spectrum of **Mono-B** measured at room temperature (KBr tablet).


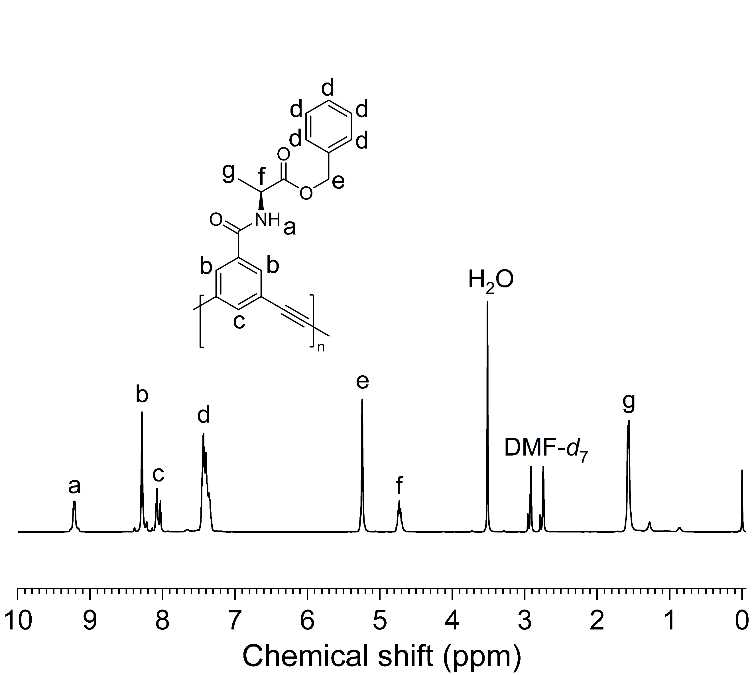


**Figure S10*.*** ^1^H NMR spectrum of **PPE-Ala-Bn** in DMSO-*d*_6_ at room temperature.


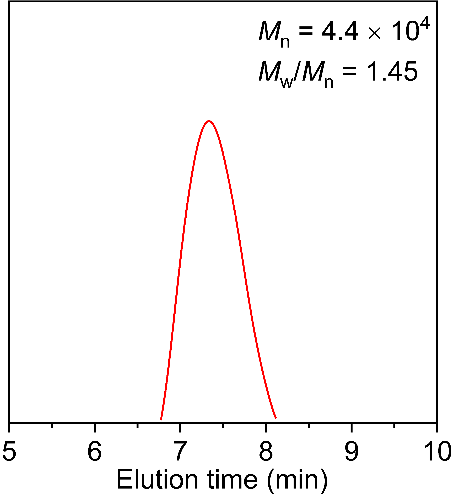


**Figure S11*.*** GPC trace obtained for **PPE-Ala-Bn** using DMF as eluent at 40 ^o^C.


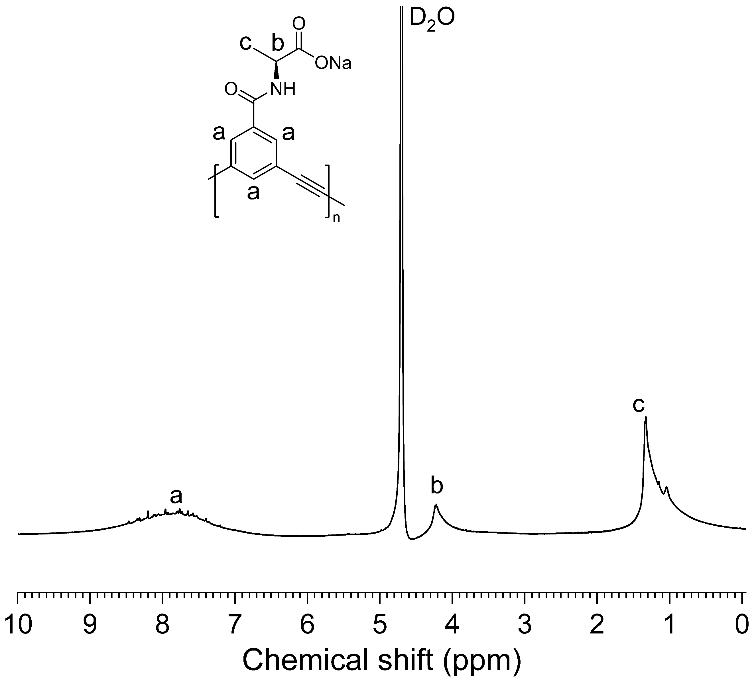


**Figure S12*.*** ^1^H NMR spectrum of **PPE-Ala-Na** in D_2_O at room temperature.


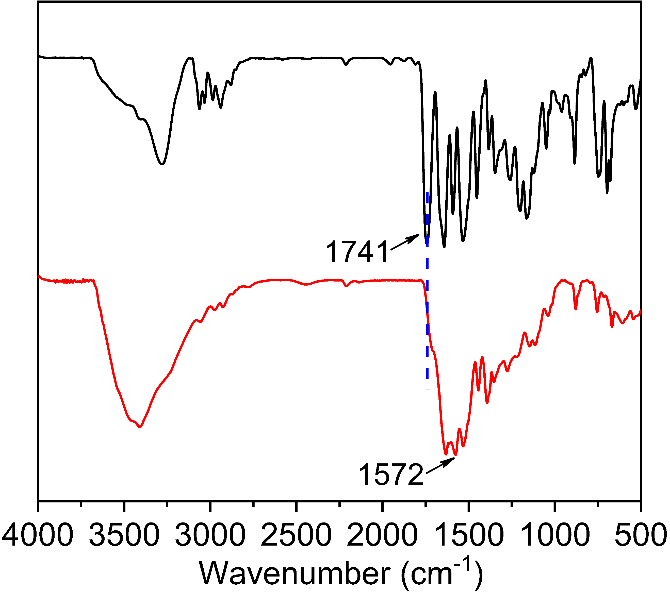


**Figure S13*.*** FTIR spectra of **PPE-Ala-Bn** (top) and **PPE-Ala-Na** (bottom) measured at room temperature (KBr tablet).

As shown in **Figures S2** and **S10**, the ethynyl proton of **Mono-A** at 4.42 ppm disappears after polymerization. Moreover, both the ≡C-H stretching vibration peak of **Mono-A** at 3281 cm^−1^ and the C-I stretching vibration peak of **Mono-B** at 578 cm^−1^ also disappear (**Figures** **S5**, **S9** and **S13**). These results imply that the polymerization is completely finished. After the hydrolysis of the resultant **PPE-Ala-Bn**, the signal of methylene protons in the benzyl moiety at 5.16 ppm disappears, indicative of the reaction completion (**Figures** **S10** and **S12**). This can be further confirmed by the absence of the C=O stretching vibration peak of benzyl ester at 1741 cm^−1^ and the presence of the C=O stretching vibration peak of carboxylate sodium at 1593 cm^−1^ (**Figure** **S13**).^[29]^

**10.** **Conformation Analysis of PPE-Ala-Na in Aqueous Solution**


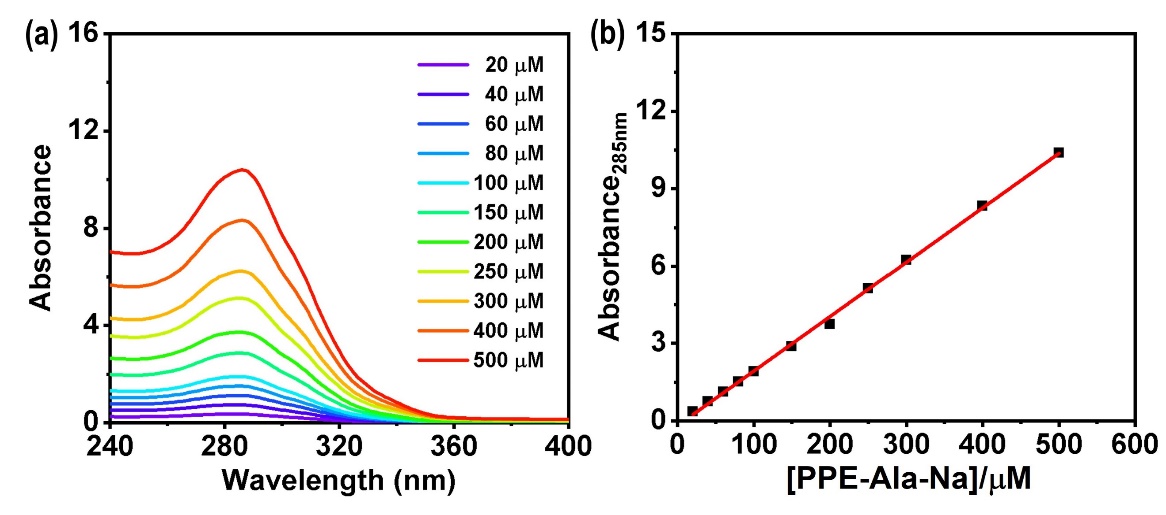


**Figure S14*.*** (a) Concentration-dependent UV−vis absorption spectra of **PPE-Ala-Na** in water at room temperature. (b) Plot of the absorption intensity at 285 nm of **PPE-Ala-Na** in water versus the concentration of **PPE-Ala-Na**.

In **Figure S14a**, **PPE-Ala-Na** shows a significant hyperchromic effect in the main chain absorption in water, as its concentration increases from 20 to 500 μM. Moreover, the absorption at 285 nm exhibits a linear relationship with concentration (**Figure S14b**), which obeys Beer’s law. This result suggests that the foldamer is in a single-molecular state.^[30]^ In other words, the observed Cotton effects for **PPE-Ala-Na** are not ascribed to the intermolecular assembly, but to the foldamer main chain.^[31]^


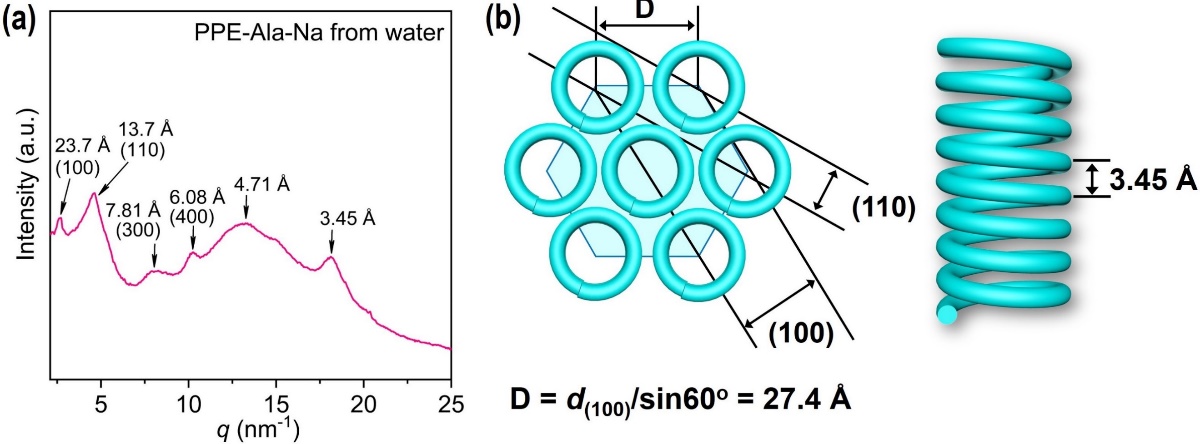


**Figure S15*.*** (a) WAXS pattern and (b) illustration of hexagonal columnar crystal of **PPE-Ala-Na** obtained by precipitating its water solution into acetone.

The powder wide-angle X-ray scattering (WAXS) pattern of **PPE-Ala-Na** is utilized to determine its crystal and geometrical structures at room temperature. In **Figure S15a**, four peaks at *d* = 23.7, 13.7, 7.81, and 6.08 Å correspond to the (100), (110), (300), and (400) reflections, respectively.^[32]^ The *q* ratio of 1:√3:3:4 suggests that the foldamer features a hexagonal columnar crystal with a lattice constant of 27.4 Å (**Figure S15b**). An amorphous halo at 4.71 Å is assigned to the average layer distance between L-alaninate sodium pendants.^[33]^ Also, a significant reflection peak at 3.45 Å is observed, corresponding to the helical pitch distance between the neighboring π-stacked phenylene ethynylene units.^[32]^


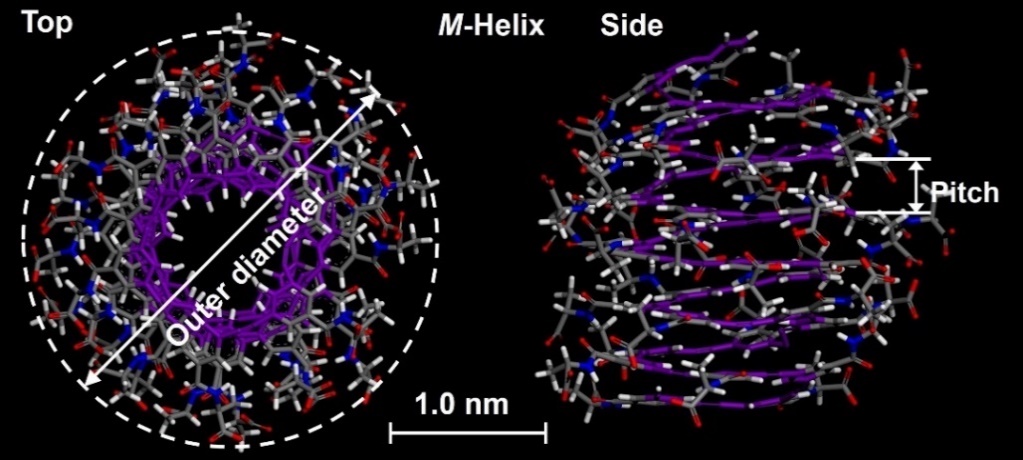


**Figure S16*.*** Top and side views of a model structure of the left (*M*)-handed helical **PPE-Ala-Na** with 36 monomeric units in water optimized by MD simulations. The structures are represented by capped-stick models. The carbon atoms of the helical main chain are highlighted in purple, and the water molecules are omitted for clarity.

Given the Cotton effect sign of **PPE-Ala-Na** is the same as the left (*M*)-handed helical poly(*m*-phenylene ethynylene) ((*R*)-**PPEa**) with ~ 6 monomer units per turn prepared by Yashima’s group,^[7]^ its helical structure in water is investigated using MD simulation of the (*M*)-handed helical foldamer model (36-mer). An energy−minimized structure of **PPE-Ala-Na** reveals that its helical pitch and outer diameter are calculated to be ⁓ 3.42 and ⁓ 27.1 Å, respectively (**Figures S16−S17**), in good agreement with the WAXS results. Furthermore, the theoretical CD and UV−vis spectra of **PPE-Ala-Na** are calculated by the TD−DFT. The negative and positive Cotton effect signs can be found at 313 and 271 nm, respectively (**Figure S18**). The absorption peak appears at 280 nm with a good fit to the experimental patterns (**Figure 3b**). Thus, it is concluded that **PPE-Ala-Na** adopts a tightly folded (*M*)-handed helix in water.


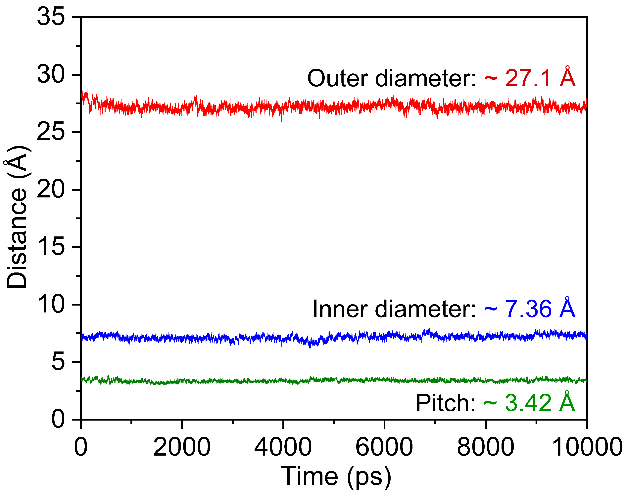


**Figure S17*.*** Plots of the outer diameter, inner diameter, and helical pitch of the (*M*)-handed helical **PPE-Ala-Na** versus the calculation time.


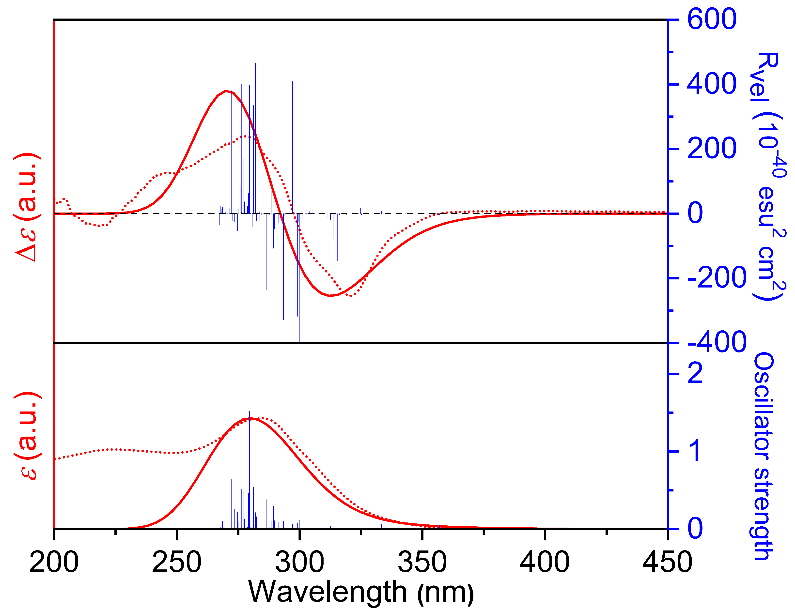


**Figure S18*.*** Solid curves: CD (top) and UV−vis absorption (bottom) spectra of a 10-mer model of **PPE-Ala-Na** simulated by the TD−DFT [ωB97X-D/6-31G*, *n*_states_ = 100] method. Vertical blue dotted lines represent *R*_vel_ and oscillator strength. Dotted curves: CD (top) and UV−vis absorption (bottom) spectra of **PPE-Ala-Na** in water at room temperature (data from **Figure 3b**).

**11. Binding Behavior of TPEBe-I with PPE-Ala-Na in Water/THF (99/1, v/v)**


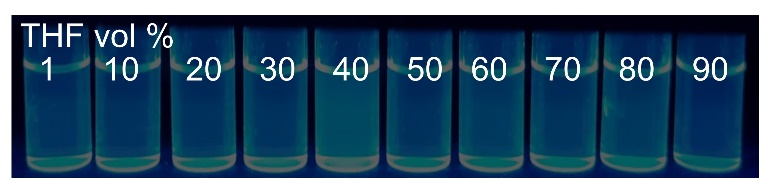


**Figure S19*.*** Fluorescence photographs of pure **TPEBe-I** in water/THF mixtures under 365 nm UV irradiation. [**TPEBe-I**] = 1 μM.





**Figure S20.** Fluorescence spectra of **TPEBe-I** in THF/water mixtures with different volume fractions of water (*f*_w_) at room temperature. [**TPEBe-I**] = 1 μM; excitation wavelength (*λ*_ex_): 420 nm.


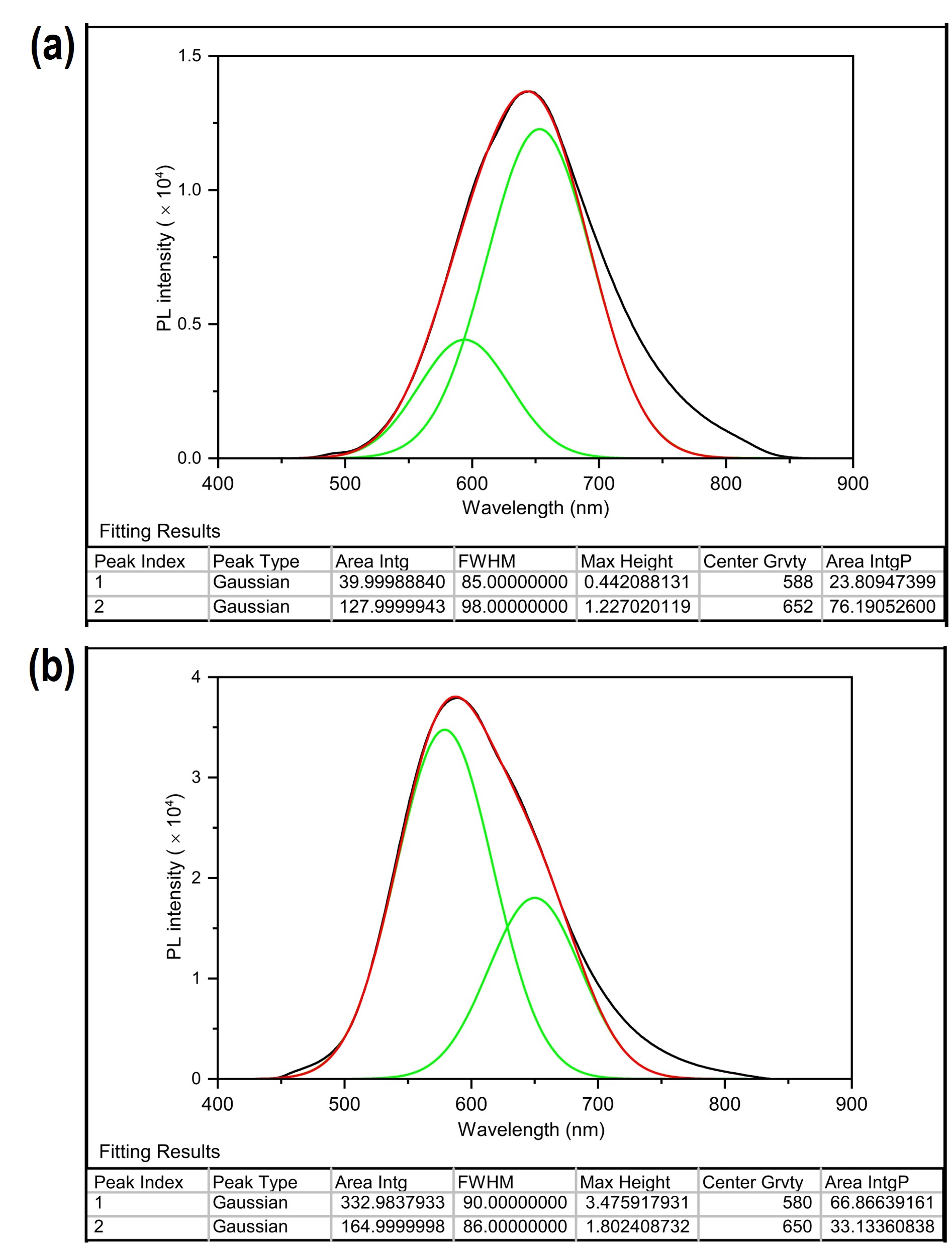


**Figure S21.** Peak-differentiation-imitating analyses of fluorescence spectra of **TPEBe-I** in the presence of (a) 2.0 and (b) 36 equiv **PPE-Ala-Na**.


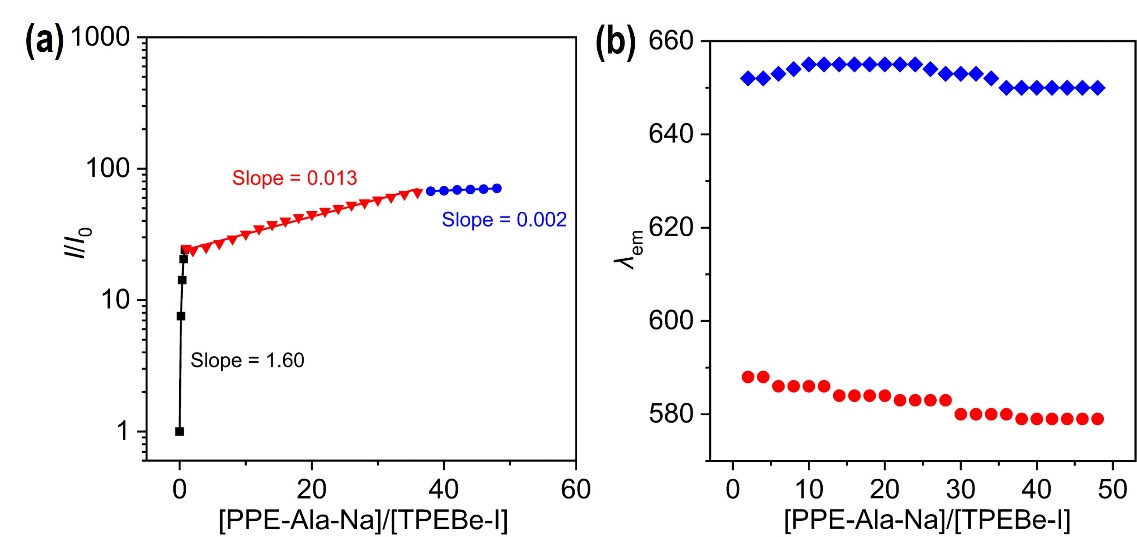


**Figure S22*.*** Plots of (a) relative fluorescence intensity (*I*/*I*_0_) and (b) emission wavelength (*λ*_em_) of **TPEBe-I** versus the concentration ratio of **PPE-Ala-Na** to **TPEBe-I**, where *I*_0_ and *I* represent the fluorescence intensity of pure **TPEBe-I** and the fluorescence maximum of **TPEBe-I** in the presence of **PPE-Ala-Na** in water/THF (99/1, v/v) at room temperature, respectively. The emission wavelength values were estimated through peak-differentiation-imitating analyses.

Upon the addition of **PPE-Ala-Na** ranging from 0 to 48 equiv, the emission intensity of **TPEBe-I** significantly increases in water/THF (99/1, v/v) (**Figure 3d**), which can be clarified as three regimes by a change of slope in the logarithmic plot of relative emission intensity (*I*/*I*_0_) versus the [**PPE-Ala-Na**]/[**TPEBe-I**] ratio (**Figure S22a**). As the foldamer concentration increases from 0 to 1.0 equiv, the slope of the curve is 1.60 and the emission intensity is enhanced by 25-fold. By increasing the foldamer concentration to 36 equiv, the slope is 0.013, indicative of a slow emission enhancement, reaching a plateau value with a 66-fold enhancement. The further addition of the foldamer to 48 equiv gives rise to the negligible emission change, resulting in a quite small slope value of 0.002. In particular, the emission peak of **TPEBe-I** displays a blue-shift from 648 to 644 nm and then to 587 nm (**Figure S22b**). Obviously, these results demonstrate that the emission behaviors of **TPEBe-I** in the presence of **PPE-Ala-Na** include two stages, probably attributed to their variable binding modes. Similar two-stage emission behavior can also be found in water/ACN (99/1, v/v) and water/DMSO (99/1, v/v) in **Figures S45**-**S46**.


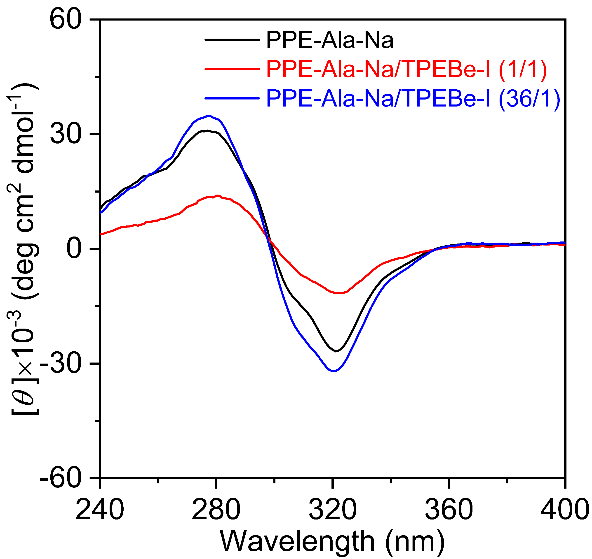


**Figure S23*.*** CD spectra of pure **PPE-Ala-Na** and the mixtures between **TPEBe-I** and **PPE-Ala-Na** at a [**TPEBe-I**]/[**PPE-Ala-Na**] ratio of 1/1 and 1/36 in water/THF (99/1, v/v) at room temperature. The concentrations of pure **PPE-Ala-Na** and pure **TPEBe-I** were 36 and 1.0 μM, respectively.

**12. Molecular Modeling and Calculations of PPE-Ala-Na/TPEBe-I Complex in Water/THF (99/1, v/v)**


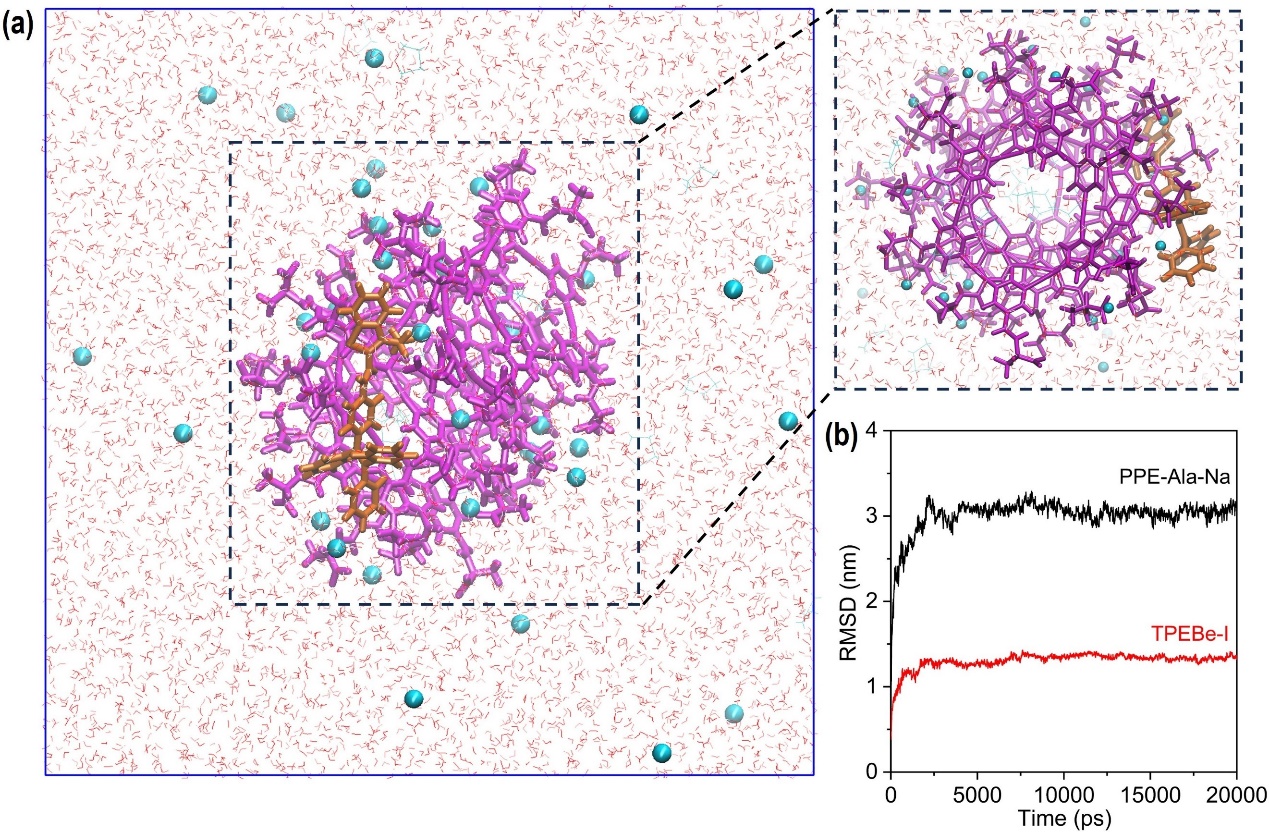


**Figure S24*.*** (a) The molecular model of the **PPE-Ala-Na**/**TPEBe-I** complex in water/THF (99/1, v/v) at 20,000 ps by MD simulations represented by stick model. The structures of **PPE-Ala-Na** and **TPEBe-I** are highlighted in purple and orange, respectively. The water and THF solvent molecules are represented by line models, which are highlighted in red and cyan, respectively. (b) The root-mean-square deviation (RMSD) plots of **PPE-Ala-Na** and **TPEBe-I** in water/THF (99/1, v/v) versus the calculation time.


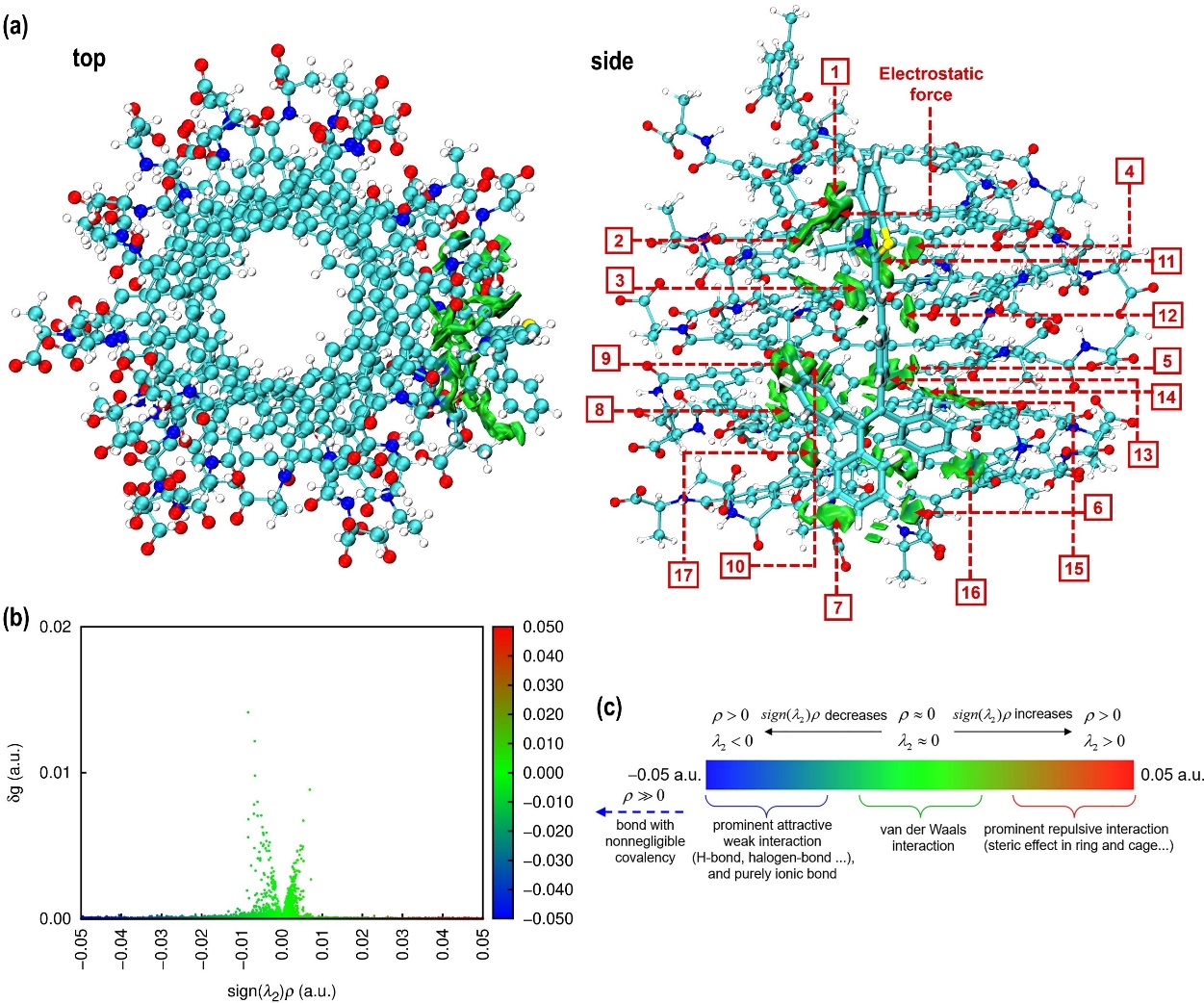


**Figure S25*.*** (a) IGMH analysis and (b) scatter plot of the non-covalent interactions between the (*M*)-handed helical **PPE-Ala-Na** and **TPEBe-I** in water/THF (99/1, v/v). (c) Common interpretation of the standard coloring method of mapped function *sign*(*λ*_2_)*ρ*.

**Table S1**. Non-covalent interactions, the corresponding distances between donor and acceptor atoms (*d*_1_ *~ d*_18_, in Å), QTAIM parameters (in a.u.) including electron density (*ρ*_BCP_), Laplacian of electron density (∇^2^*ρ*_BCP_), kinetic energy density (*G*_BPC_), potential energy density (*V*_BPC_), and local energy density (*H*_BPC_) at the intermolecular bond critical points (BCPs), and interaction energy values (*E*, in kcal/mol) for the **PPE-Ala-Na**/**TPEBe-I** complex in **Figure S22**.

| entry | Noncovalent interaction | *d* | *ρ*_BCP_ | ∇^2^*ρ*_BCP_ | *G*_BPC_ | *V*_BPC_ | *H*_BPC_ | *E*^a^ |
| --- | --- | --- | --- | --- | --- | --- | --- | --- |
| 1 | C−H···O | 2.317 | 0.0083 | 0.0537 | 0.0080 | −0.0065 | 0.0035 | −2.0394 |
| 2 | C−H···O | 2.429 | 0.0067 | 0.0447 | 0.0080 | −0.0048 | 0.0032 | −1.5060 |
| 3 | C−H···O | 2.237 | 0.0100 | 0.0636 | 0.0121 | −0.0084 | 0.0038 | −2.6355 |
| 4 | C−H···S | 3.061 | 0.0031 | 0.0176 | 0.0030 | −0.0016 | 0.0014 | −0.5020 |
| 5 | C−H···π | 3.699 | 0.0041 | 0.0209 | 0.0036 | −0.0019 | 0.0017 | −0.5961 |
| 6 | C−H···O | 2.492 | 0.0061 | 0.0425 | 0.0074 | −0.0043 | 0.0032 | −1.3491 |
| 7 | C−H···π | 3.588 | 0.0034 | 0.0186 | 0.0032 | −0.0017 | 0.0015 | −0.5334 |
| 8 | C−H···π | 3.199 | 0.0050 | 0.0281 | 0.0049 | −0.0028 | 0.0021 | −0.8775 |
| 9 | C−H···O | 2.456 | 0.0065 | 0.0420 | 0.0074 | −0.0044 | 0.0031 | −1.3805 |
| 10 | C−H···O | 2.385 | 0.0071 | 0.0478 | 0.0086 | −0.0052 | 0.0034 | −1.6315 |
| 11 | C−H···O | 2.512 | 0.0053 | 0.0267 | 0.0047 | −0.0027 | 0.0020 | −0.8471 |
| 12 | C−H···O | 2.682 | 0.0038 | 0.0396 | 0.0029 | −0.0083 | 0.0054 | −2.6042 |
| 13 | C−H···π | 2.442 | 0.0085 | 0.0433 | 0.0079 | −0.0050 | 0.0029 | −1.5688 |
| 14 | C−H···π | 3.434 | 0.0020 | 0.0112 | 0.0019 | −0.0009 | 0.0009 | −0.2824 |
| 15 | C−H···O | 2.506 | 0.0059 | 0.0320 | 0.0056 | −0.0032 | 0.0024 | −1.0040 |
| 16 | C−H···O | 2.334 | 0.0085 | 0.0536 | 0.0100 | −0.0065 | 0.0034 | −2.0394 |
| 17 | C−H···O | 2.454 | 0.0062 | 0.0419 | 0.0074 | −0.0043 | 0.0031 | −1.3491 |

^a^ Calculated on the basis of the equation *E* = *V*_BPC_/2.^[34]^

**13. Conformational Transitions of PPE-Ala-Na with and without TPEBe-I in Water/THF Mixtures**


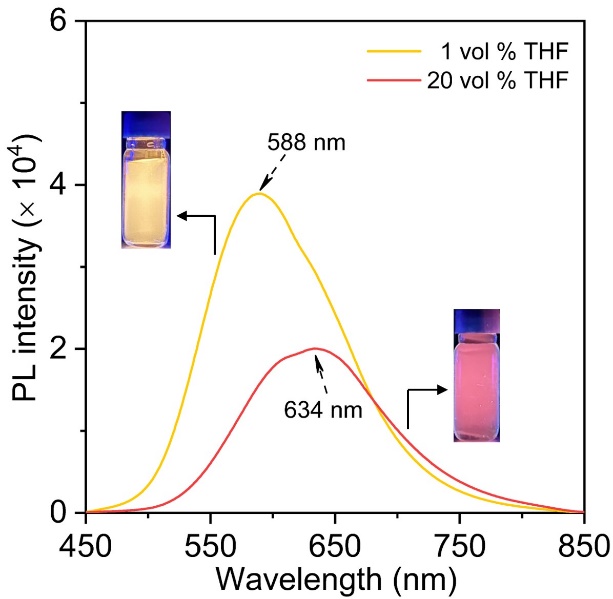


**Figure S26.** Fluorescence spectra of **TPEBe-I** complexed with **PPE-Ala-Na** in water/THF mixtures with 1 and 20 vol% THF, respectively, obtained by direct titration of THF to the initial foldamer aqueous solution. The initial concentration of **PPE-Ala-Na** in water was 36 μM. [**TPEBe-I**]/[**PPE-Ala-Na**] = 1/36; excitation wavelength (*λ*_ex_): 420 nm. Inset: Fluorescent photographs of **TPEBe-I** complexed with **PPE-Ala-Na** in water/THF mixtures with 1 and 20 vol% THF under 365 nm UV irradiation, respectively.


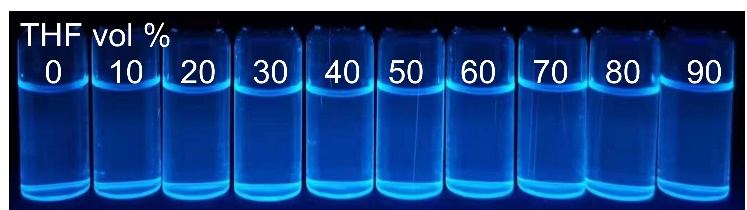


**Figure S27*.*** Fluorescence photographs of pure **PPE-Ala-Na** in water/THF mixtures under 365 nm UV irradiation. [**PPE-Ala-Na**] = 36 μM.


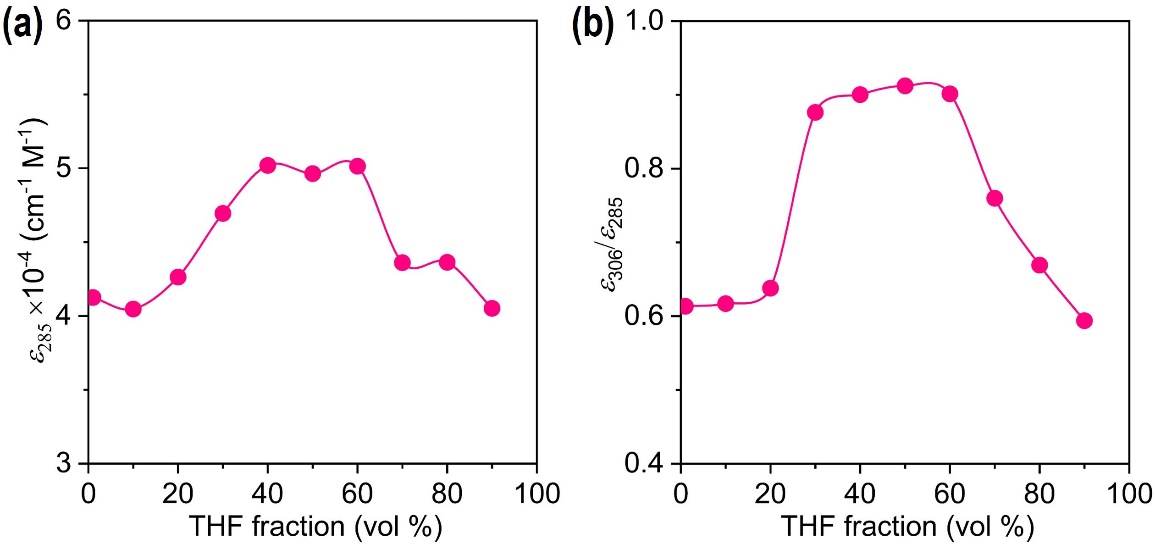


**Figure S28*.*** Plots of (a) absorption intensity at 285 nm and (b) absorption intensity ratio at 306 and 285 nm of **PPE-Ala-Na** in the presence of **TPEBe-I** versus the volume fraction of THF.

As a typical kind of poly(*m*-phenylene ethynylene)-based foldamer, the ratio of the absorption intensity at 306 and 285 nm for **PPE-Ala-Na** can reveal its main chain conformation.^[35]^ A high value of *ε*_306_/*ε*_285_ denotes a random mixture of *cisoid* and *transoid* conformers, while a low value corresponds to a predominantly *cisoid* or folded conformation.^[36]^ In **Figure S28b**, the *ε*_306_/*ε*_285_ value exhibits a negligible increase when the THF fraction is less than 20 vol%, because **PPE-Ala-Na** still possesses a folded helical conformation. However, this value sharply increases at the THF fraction range from 30 to 60 vol%, which is as high as 0.91. Obviously, the resultant curve shows a sigmoidal shape, indicative of a cooperative conformational transition.^[37]^ Moreover, the further addition of THF to 90 vol% leads to a significant decrease of the *ε*_306_/*ε*_285_ value, attributing to the refolding behavior of **PPE-Ala-Na**. It is noted that the quantitative relationship between the random-coil content (*X*_C_) and *ε*_306_/*ε*_285_ value with the THF fraction can be built up (see Section 4 in the Supporting Information).





**Figure S29.** CD (top) and UV−vis absorption (bottom) spectra of **PPE-Ala-Na** complexed with **TPEBe-I** in water/THF mixtures with 1 and 20 vol% THF at room temperature, through direct titration of THF to the initial foldamer aqueous solution, followed by further addition of water. The initial concentration of **PPE-Ala-Na** in water was 360 μM. [**TPEBe-I**]/[**PPE-Ala-Na**] = 1/36.


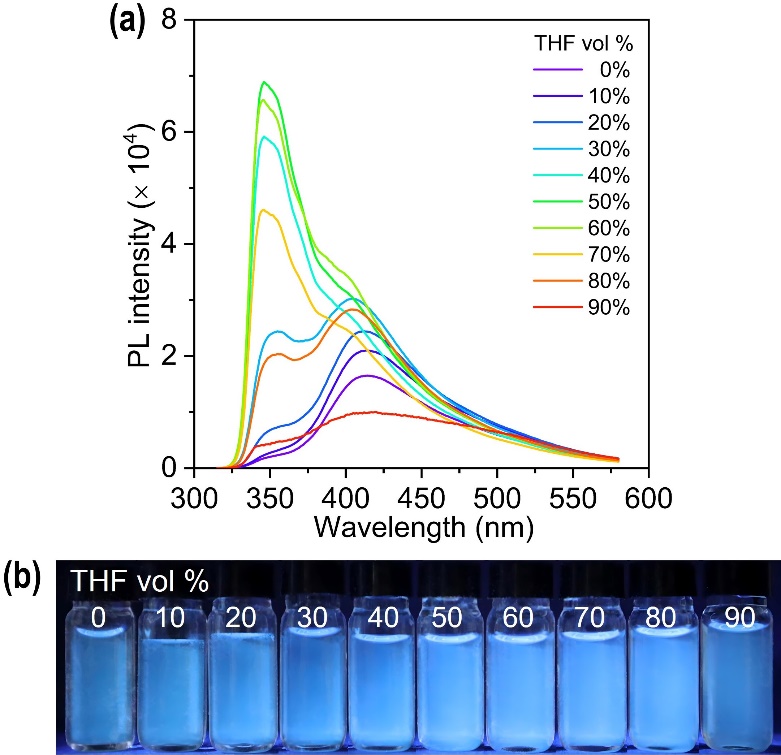


**Figure S30*.*** (a) Fluorescence spectra of **PPE-Ala-Na** in water/THF mixtures with different THF fractions. [**PPE-Ala-Na**] = 100 μM; excitation wavelength (*λ*_ex_): 295 nm. (b) Fluorescence photographs of **PPE-Ala-Na** in water/THF mixtures under 365 nm UV irradiation.

**Figure S30a** shows that **PPE-Ala-Na** exhibits an obvious emission peak at 415 nm, arising from an excimer-like excited state owing to the π-π stacking interaction between neighboring phenylene ethynylene units in the helical conformation.^[38]^ As the THF fraction is increased to 20 vol%, the emission intensity at 415 nm is enhanced, indicative of the weakened π-π stacking interaction in the foldamer main chain.^[39]^ Namely, **PPE-Ala-Na** forms an extended helical conformation. At 30 vol%, a new emission peak appears at 355 nm, which emanates from a relatively localized excited state due to the random-coil conformation.^[38]^ Upon the further addition of THF to 50 vol%, the emission intensity at 355 nm reaches a maximum value, suggesting that the foldamer experiences a partial helix-coil transition. However, when the THF fraction increases to 90 vol%, this emission peak gradually decreases and disappears at 90 vol% THF. **Figure S31** demonstrates that **PPE-Ala-Na** always exists at a single-molecular state in the presence of THF. In other words, the foldamer finally returns to the folded state. Unfortunately, the THF-triggered conformational transitions of **PPE-Ala-Na** cannot be visualized by its intrinsic blue emission (**Figure S30b**).


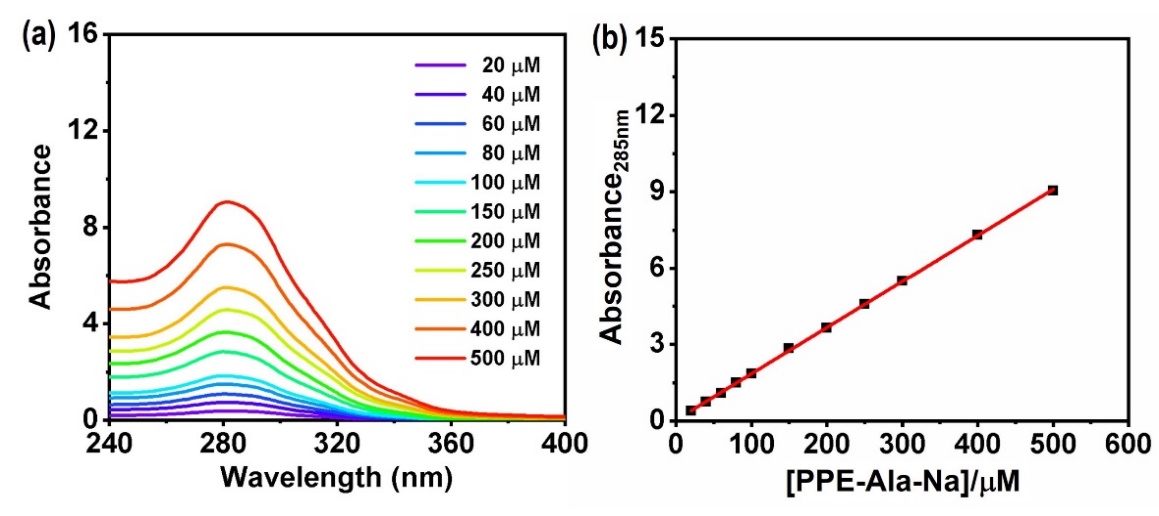


**Figure S31*.*** (a) Concentration-dependent UV−vis absorption spectra of **PPE-Ala-Na** in water/THF (10/90, v/v) at room temperature. (b) Plot of the absorption intensity at 285 nm of **PPE-Ala-Na** in water/THF (10/90, v/v) versus the concentration of **PPE-Ala-Na**.


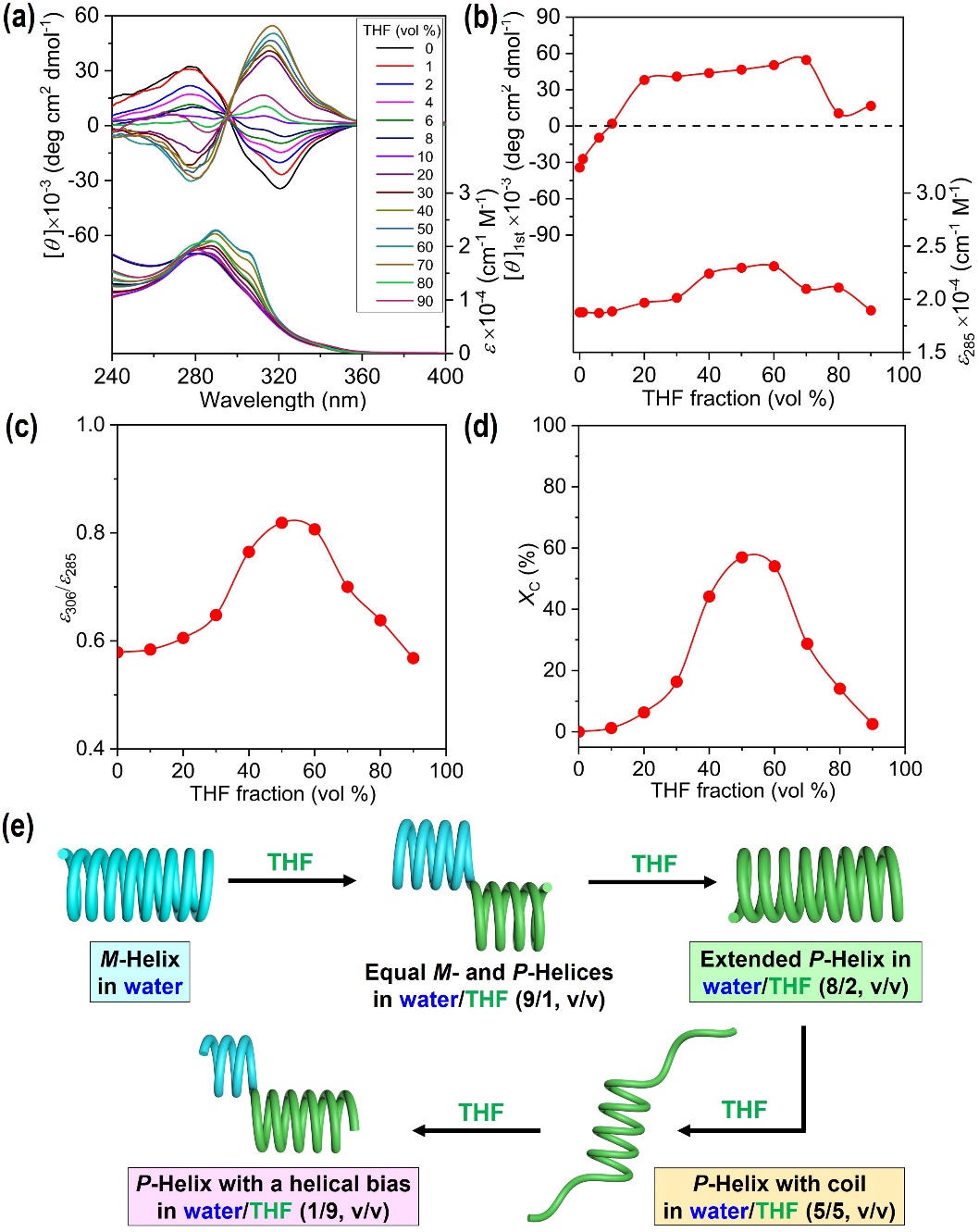


**Figure S32*.*** (a) CD (top) and UV−vis absorption (bottom) spectra of **PPE-Ala-Na** in water/THF mixtures at room temperature. [**PPE-Ala-Na**] = 100 μM. Plots of the first Cotton effect intensity (b, top), absorption intensity at 285 nm (b, bottom), absorption intensity ratio at 306 and 285 nm (c), and the content of random-coil conformation (d) versus the volume fraction of THF. (e) Illustrative conformational transitions of **PPE-Ala-Na** in water/THF mixtures.

As the THF fraction increases, the first Cotton effect decreases and almost disappears in the presence of 10 vol% THF, followed by the sign change from negative to positive when the THF fraction is 20 vol% (**Figure S32a**-**b**). Meanwhile, the absorption intensity at 285 nm increases due to the weakened π-π stacking interaction between neighboring phenylene ethynylene units.^[40]^ So, the foldamer undergoes a conformational change from (*M*)-handed helix to an equal content of (*M*)- and (*P*)-handed helices,^[41]^ and then to a slightly extended (*P*)-handed one (**Figure S32e**). When the THF fraction increases to 50 vol%, the absorption at 285 nm exhibits a significant hyperchromic effect, accompanied by a red-shift to 290 nm. At the same time, a new absorption at 306 nm corresponding to the *transoid* conformer of *m*-phenylene ethynylene units gradually increases, suggesting that a partial helix-coil transition occurs^[42]^ (**Figure S32e**). The *X*_C_ value is calculated to be 57% at 50 vol% THF (**Figure S32c**-**d**). By the further addition of THF to 90 vol%, the absorption at 290 nm is decreased and blue-shifted to 280 nm, while the absorption at 306 nm gradually disappears, indicative of a refolding behavior. However, the Cotton effect intensity of +16.6 × 10^3^ deg cm^2^ dmol^−1^ is less than the initial value in water (**Figure S32a**-**b**), implying that the refolded helical segments are (*M*)-handed. In other words, the foldamer features a (*P*)-handed helix with a helical sense bias (**Figure S32e**).


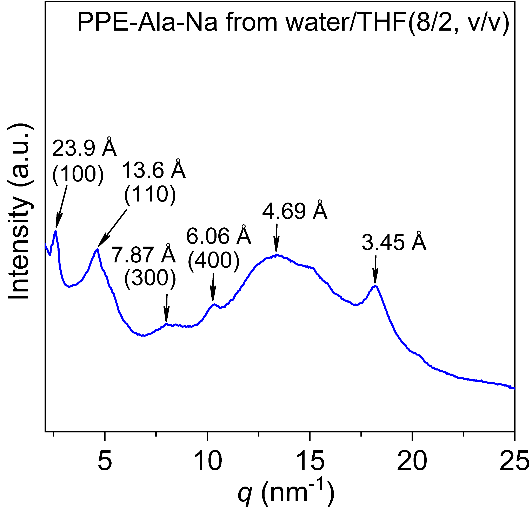


**Figure S33*.*** The WAXS pattern of **PPE-Ala-Na** obtained by precipitating its water/THF (80/20, v/v) solution into acetone.

**Figure S33** shows that **PPE-Ala-Na** obtained from water/THF (80/20, v/v) also forms a tetragonal columnar crystal. Moreover, a reflection peak at 3.45 Å corresponding to the helical pitch distance can be found. Obviously, such a small pitch value is not consistent with the extended helical conformation of **PPE-Ala-Na** in water/THF (80/20, v/v). Notably, the extended helix is stabilized by the solvent effect. Therefore, once the solvent is removed, it cannot be retained and memorized in the solid state,^[43]^ and finally reverts to the tightly folded helix.


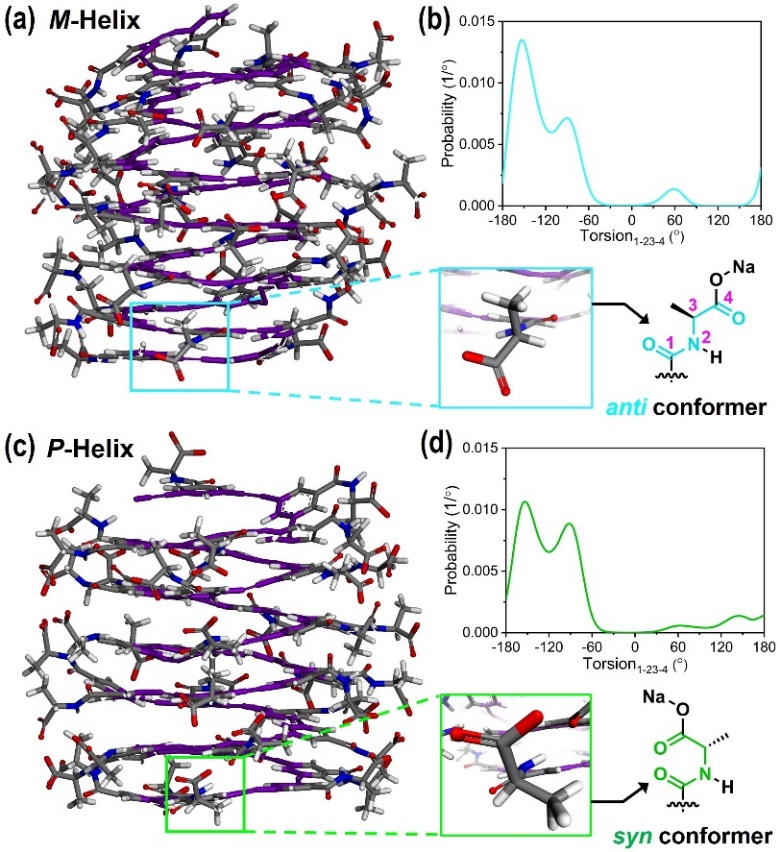


**Figure S34*.*** (a) Representative *anti* orientation between two carbonyl groups and (b) probability distribution function of the OC-N-C-CO (1-23-4) dihedral angle of L-alanine sodium pendants in the model structure of the (*M*)-handed helical **PPE-Ala-Na** in water. (c) Representative *syn* orientation between two carbonyl groups and (d) probability distribution function of the OC-N-C-CO (1-23-4) dihedral angle of L-alanine sodium in the model structure of the (*P*)-handed helical **PPE-Ala-Na** in water/THF (80/20, v/v).

The mechanism of the THF-triggered helix inversion in **PPE-Ala-Na** can be elucidated by the MD simulations. The probability distribution of the OC-N-C-CO (1-23-4) dihedral angle of L-alanine sodium pendants in (*M*)-handed helical **PPE-Ala-Na** exhibits a sharp peak at −150° in addition to a shoulder around −90° (**Figure S34a**-**b**), indicating that the carbonyls mainly adopt an antiperiplanar (*anti*) orientation in water.^[44]^ For the (*P*)-handed helical **PPE-Ala-Na** in water/THF (80/20, v/v), the peak of the dihedral angle distribution at −150° decreases, but the peak at −90° increases (**Figure S34c**-**d**). In other words, some *anti*-oriented carbonyls are transformed into synperiplanar (*syn*) ones. As a donor solvent with a medium polarity, THF has a good solvation effect toward both the main chain and the side group.^[45]^ Accordingly, THF tends to interact with the L-alanine sodium pendants, which increases the repulsion between them.^[39]^ Such a steric hindrance is released by changing the *anti*-oriented carbonyl groups into the *syn*-oriented ones.^[46]^ Subsequently, the foldamer regulates its folded conformation to compensate the repulsion effect by increasing the pitch distance and even inverting the screw sense,^[46]^ so an extended folded helix with an opposite handedness is formed. The resultant helical pitch and outer diameter are estimated to be ⁓ 3.64 and ⁓ 26.8 Å, respectively (**Figure S35**). The addition of 50 vol% THF further increases the steric effect, which destroys the π-π stacking interaction between some neighboring *m*-phenylene ethynylene units, and thus leads to a partial helix-coil transition. However, at a high organic solvent fraction of 90 vol% THF, the intrinsic chirality of L-alanine sodium pendants plays a key role in the helix induction for **PPE-Ala-Na**.^[47]^ So, the random-coil segments refold into (*M*)-handed helical ones, resulting in the formation of a (*P*)-handed helix with a helical sense bias.


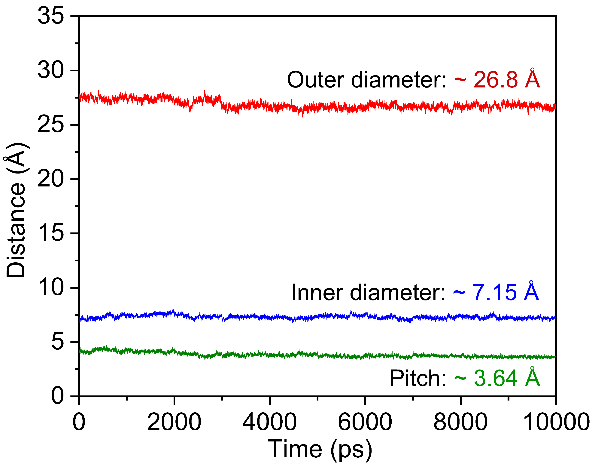


**Figure S35*.*** Plots of the outer diameter, inner diameter, and helical pitch of the (*P*)-handed helical **PPE-Ala-Na** in water/THF (80/20, v/v) versus the calculation time.

**14. Molecular Modeling and Calculations of PPE-Ala-Na/TPEBe-I Complex in Water/THF (80/20, v/v)**


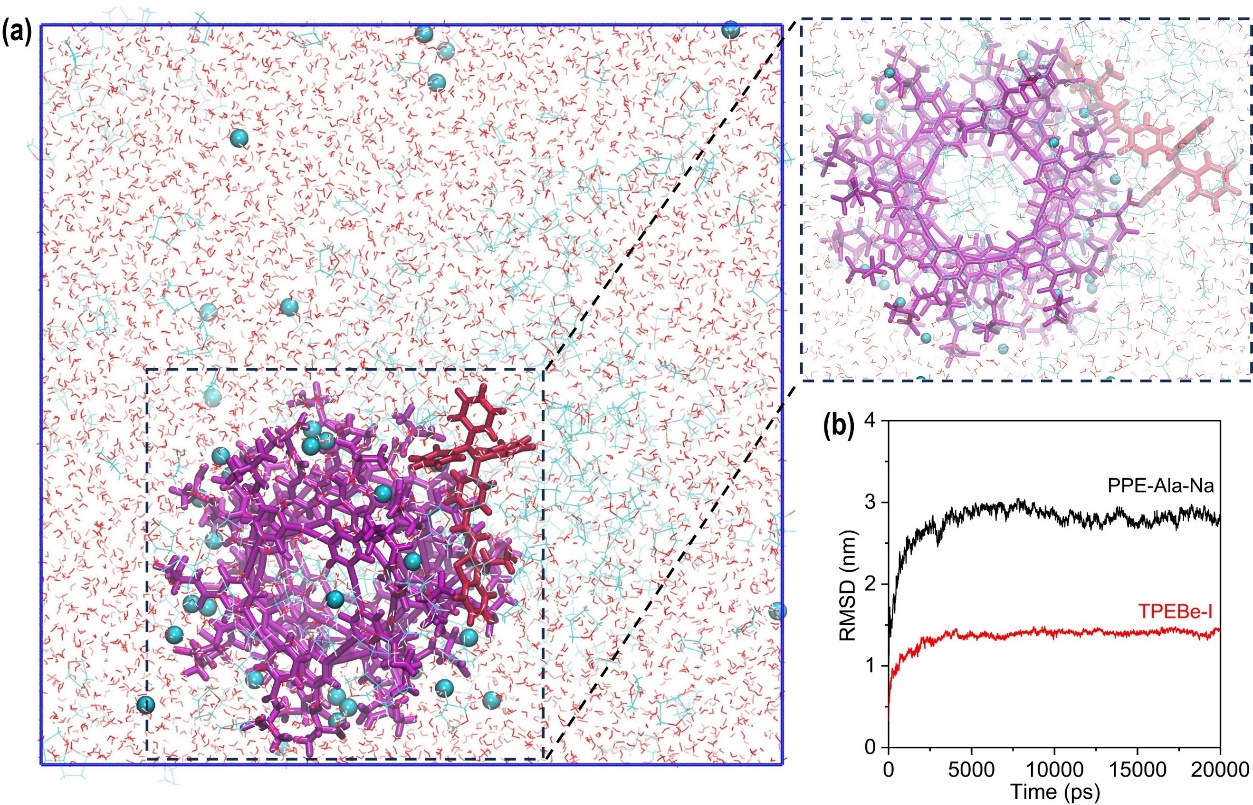


**Figure S36*.*** (a) The molecular model of the **PPE-Ala-Na**/**TPEBe-I** complex in water/THF (80/20, v/v) at 20,000 ps by MD simulations represented by the stick model. The structures of **PPE-Ala-Na** and **TPEBe-I** are highlighted in purple and dark red, respectively. The water and THF solvent molecules are represented by line models, which are highlighted in red and cyan, respectively. (b) RMSD plots of **PPE-Ala-Na** and **TPEBe-I** in water/THF (80/20, v/v) versus the calculation time.


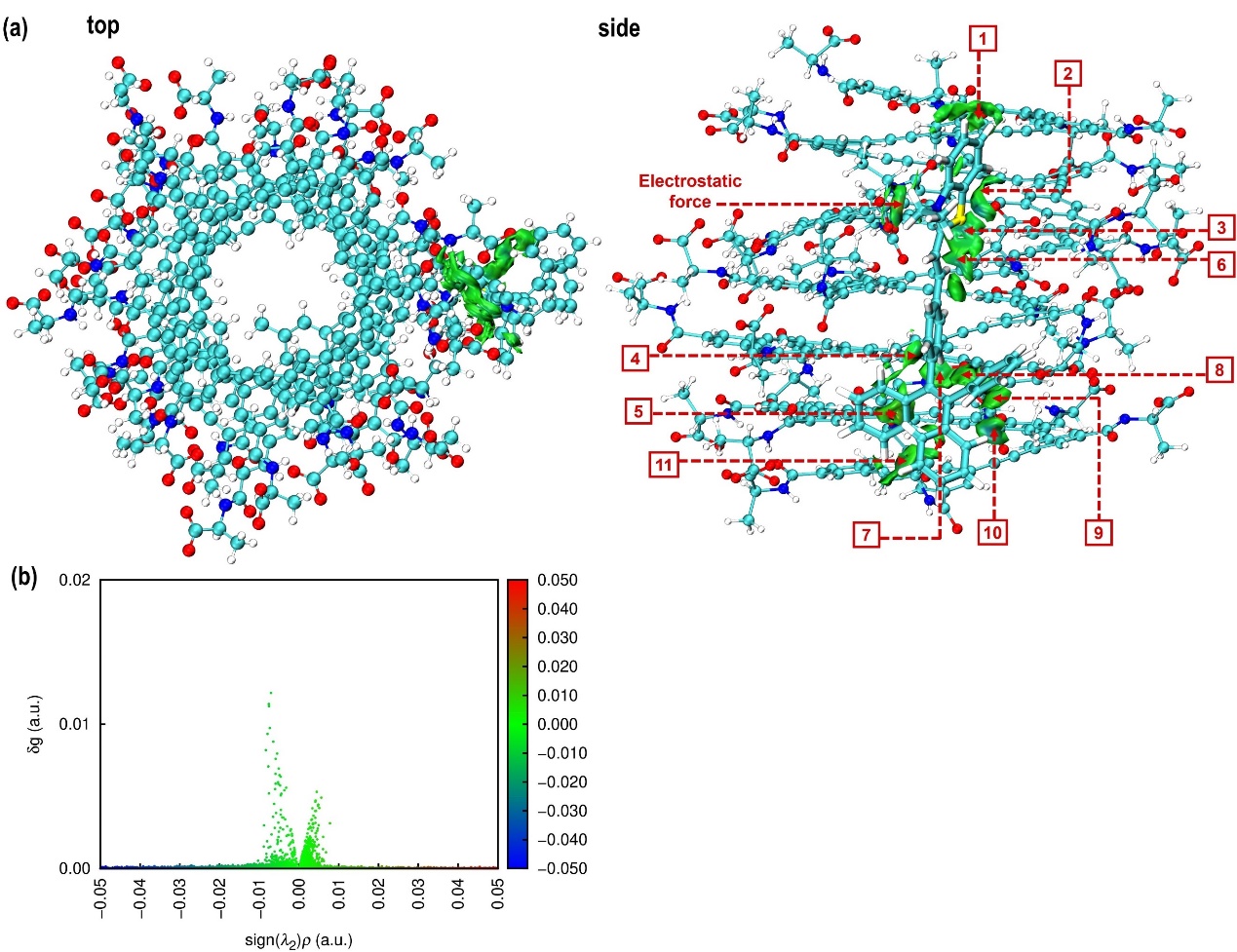


**Figure S37*.*** (a) IGMH analysis and (b) scatter plot of the non-covalent interactions between the (*P*)-handed helical **PPE-Ala-Na** and **TPEBe-I** in water/THF (80/20, v/v).

**Table S2**. Non-covalent interactions, the corresponding distances between donor and acceptor atoms (*d*_1_ *~ d*_18_, in Å), QTAIM parameters (in a.u.) including electron density (*ρ*_BCP_), Laplacian of electron density (∇^2^*ρ*_BCP_), kinetic energy density (*G*_BPC_), potential energy density (*V*_BPC_), and local energy density (*H*_BPC_) at the intermolecular bond critical points (BCPs), and interaction energy values (*E*, in kcal/mol) for the **PPE-Ala-Na**/**TPEBe-I** complex in **Figure S32**.

| entry | Noncovalent interaction | *d* | *ρ*_BCP_ | ∇^2^*ρ*_BCP_ | *G*_BPC_ | *V*_BPC_ | *H*_BPC_ | *E*^a^ |
| --- | --- | --- | --- | --- | --- | --- | --- | --- |
| 1 | C−H···O | 2.482 | 0.0060 | 0.0423 | 0.0074 | −0.0043 | 0.0031 | −1.3491 |
| 2 | C−H···O | 2.424 | 0.0067 | 0.0463 | 0.0083 | −0.0051 | 0.0033 | −1.6001 |
| 3 | C−H···S | 2.649 | 0.0089 | 0.0456 | 0.0087 | −0.0060 | 0.0027 | −1.8825 |
| 4 | C−H···π | 3.345 | 0.0059 | 0.0306 | 0.0055 | −0.0033 | 0.0022 | −1.0354 |
| 5 | C−H···π | 2.478 | 0.0067 | 0.0349 | 0.0062 | −0.0037 | 0.0025 | −1.1609 |
| 6 | C−H···π | 3.661 | 0.0055 | 0.0280 | 0.0049 | −0.0029 | 0.0021 | −0.9099 |
| 7 | C−H···N | 2.567 | 0.0078 | 0.0400 | 0.0073 | −0.0047 | 0.0027 | −1.4746 |
| 8 | C−H···O | 2.409 | 0.0072 | 0.0498 | 0.0090 | −0.0055 | 0.0035 | −1.7256 |
| 9 | C−H···O | 2.727 | 0.0049 | 0.0343 | 0.0060 | −0.0034 | 0.0026 | −1.0668 |
| 10 | C−H···O | 2.274 | 0.0083 | 0.0553 | 0.0102 | −0.0065 | 0.0037 | −2.0394 |
| 11 | C−H···O | 2.442 | 0.0067 | 0.0461 | 0.0082 | −0.0049 | 0.0033 | −1.5374 |

^a^ Calculated on the basis of the equation *E* = *V*_BPC_/2.^[34]^

**
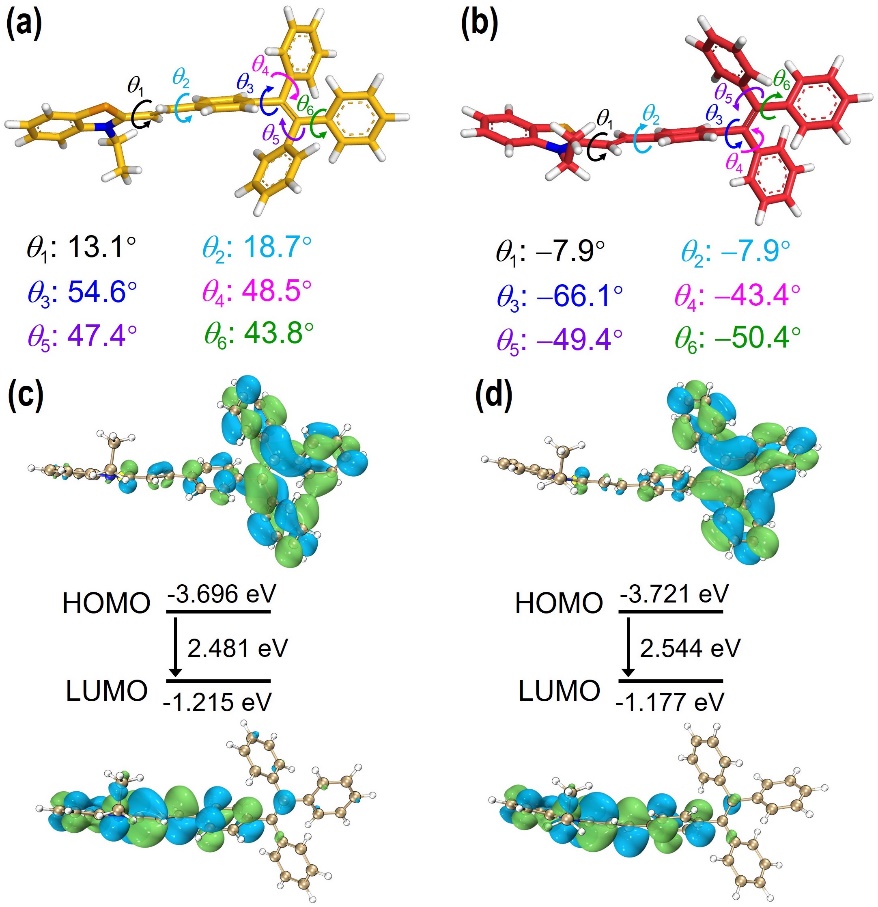
**

**Figure S38*.*** (a, b) Torsion angles (*θ*_1_ ~ *θ*_6_) and (c, d) molecular orbitals of **TPEBe-I** complexed with (a, c) (*M*)-handed and (b, d) (*P*)-handed helical **PPE-Ala-Na**.

When bound with the (*M*)-handed and (*P*)-handed helical **PPE-Ala-Na**, the single bonds of the dye twist in an opposite screw sense, which is probably directed by the handedness in the foldamer (**Figure S38a**-**b**). The energy gap between HOMO and LUMO of the dye only show a slight increase from 2.481 to 2.544 eV (**Figure S38c**-**d**), implying that the conformational difference almost has no effect on the intramolecular charge transfer (ICT).

**15. Conformational Transitions of PPE-Ala-Na in Water/ACN and Water/DMSO Mixtures**


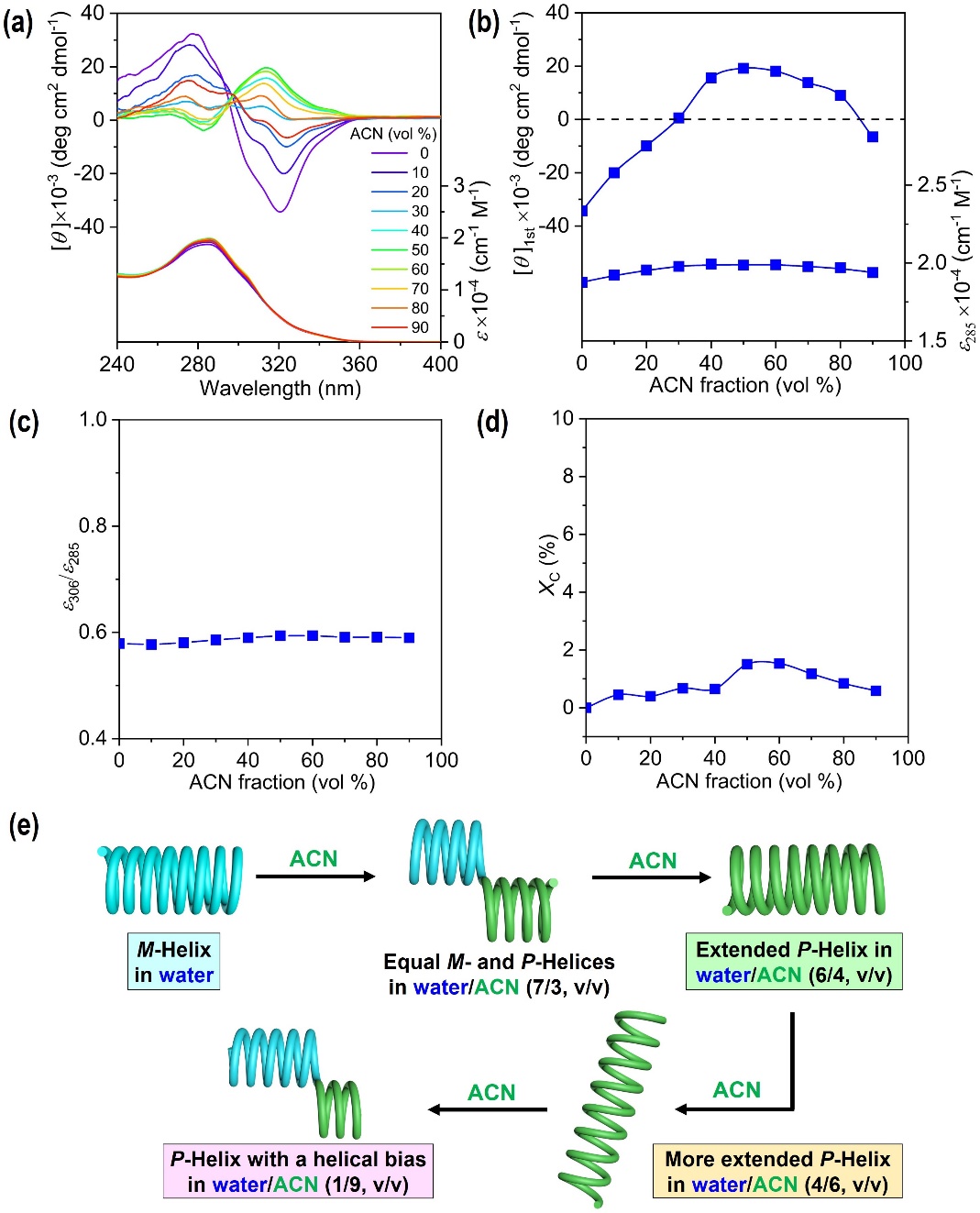


**Figure S39*.*** (a) CD (top) and UV−vis absorption (bottom) spectra of **PPE-Ala-Na** in water/ACN mixtures. [**PPE-Ala-Na**] = 100 μM. Plots of the first Cotton effect intensity (b, top), absorption intensity at 285 nm (b, bottom), absorption intensity ratio at 306 and 285 nm (c), and the content of random-coil conformation (d) versus the volume fraction of ACN. (e) Illustrative conformational transitions of **PPE-Ala-Na** in water/ACN mixtures.

As a folding-promoting solvent, ACN is good for the side group, but poor for the main chain.^[48]^ So, in the presence of ACN, we cannot observe the absorption peak at 306 nm (**Figure S39a**), because no helix-coil transition occurs in **PPE-Ala-Na**. This result is further confirmed by the emission spectral patterns (**Figure S40**). So, the *ε*_306_/*ε*_285_ and *X*_C_ values almost do not change (**Figure S39c**-**d**). The first Cotton effect gradually decreases until it disappears at 30 vol% (**Figure 39a**-**b**). However, its sign changes from negative to positive at higher ACN fractions from 40 to 60 vol%. At the same time, a hyperchromic effect at 285 nm can be observed in the absorption spectrum. These results indicate that the foldamer takes an extended (*P*)-handed helical conformation (**Figure 39e**). As the ACN fraction increases to 90 vol%, the absorption at 285 nm shows a slight hypochromic effect, accompanied by the change of the first Cotton effect sign from positive to negative. In this case, the foldamer is still at a single-molecular state (**Figure S41**). Most of the (*P*)-handed helical segments are transformed into the (*M*)-handed helical ones, which is driven by the solvophobic effect and meanwhile directed by the chiral L-alanine sodium pendants.^[6]^ Therefore, a (*M*)-handed helix with a helical sense bias is obtained (**Figure 39e**).


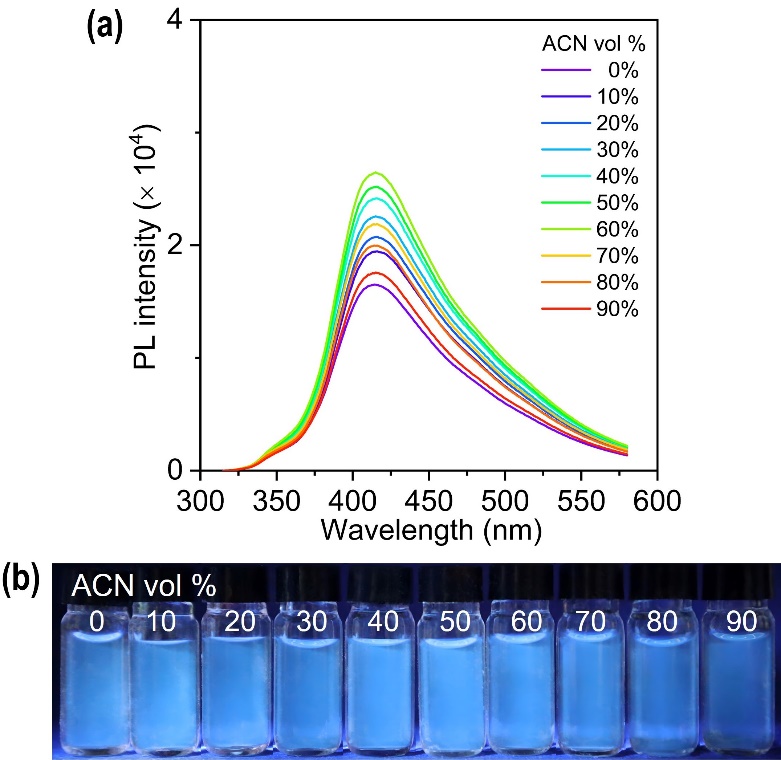


**Figure S40*.*** (a) Fluorescence spectra of **PPE-Ala-Na** in water/ACN mixtures with different ACN fractions. [**PPE-Ala-Na**] = 100 μM; excitation wavelength (*λ*_ex_): 295 nm. (b) Fluorescence photographs of **PPE-Ala-Na** in water/ACN mixtures under 365 nm UV irradiation.


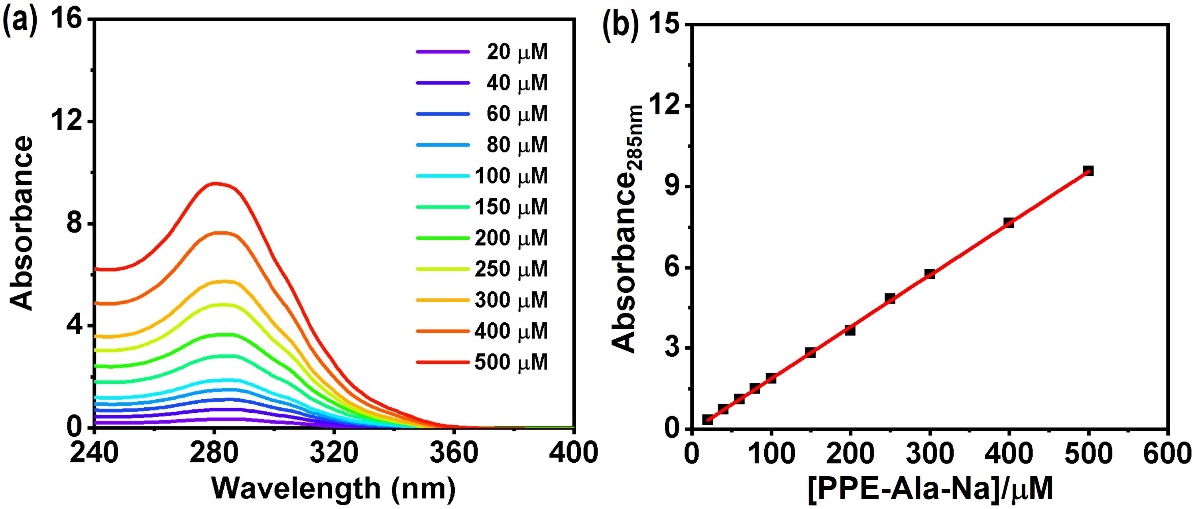


**Figure S41*.*** (a) Concentration-dependent UV−vis absorption spectra of **PPE-Ala-Na** in water/ACN (10/90, v/v) at room temperature. (b) Plot of the absorption maxima of **PPE-Ala-Na** in water/ACN (10/90, v/v) versus the concentration of **PPE-Ala-Na**.


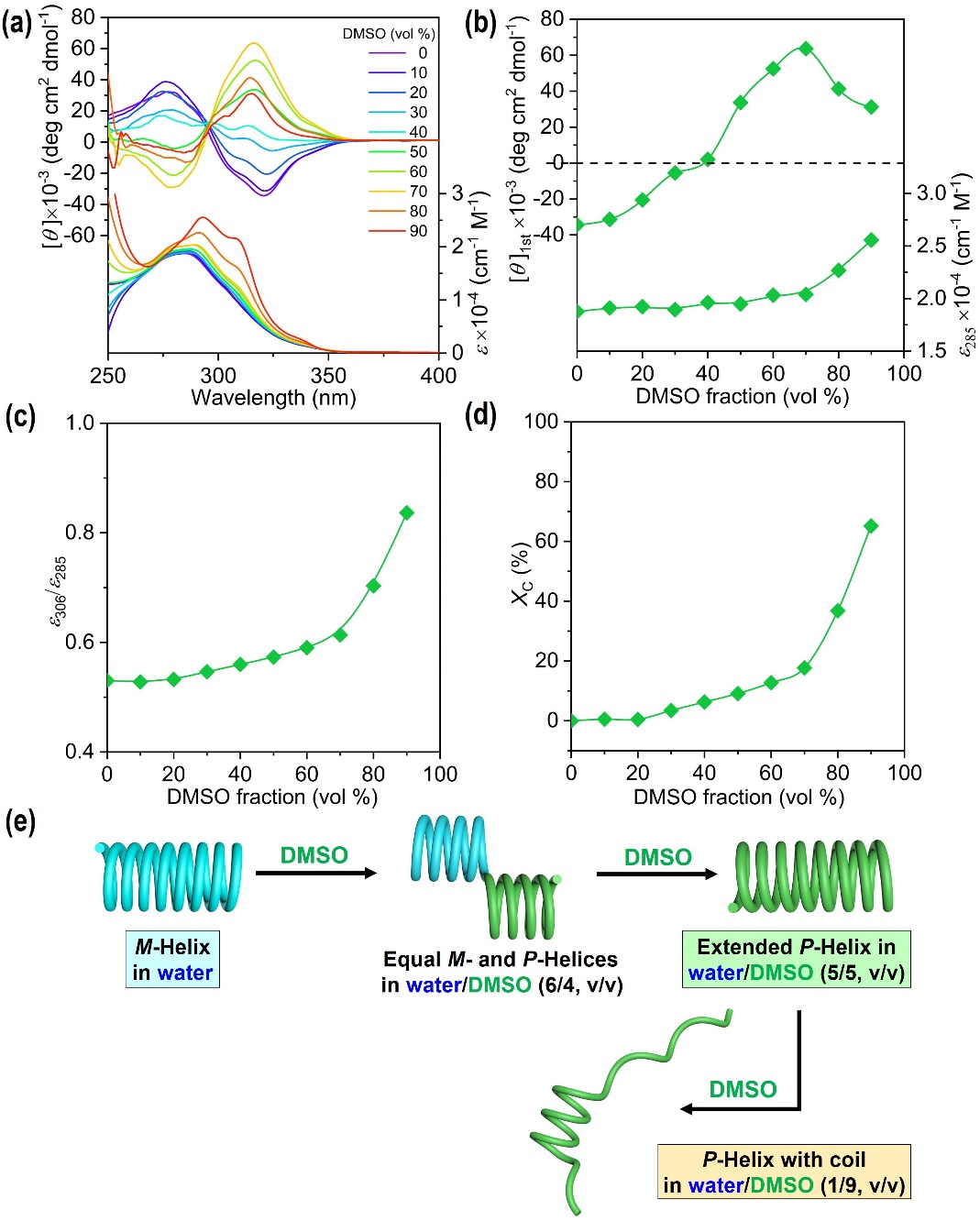


**Figure S42*.*** (a) CD (top) and UV−vis absorption (bottom) spectra of **PPE-Ala-Na** in water/DMSO mixtures. [**PPE-Ala-Na**] = 100 μM. Plots of the first Cotton effect intensity (b, top), absorption intensity at 285 nm (b, bottom), absorption intensity ratio at 306 and 285 nm (c), and the content of random-coil conformation (d) versus the volume fraction of DMSO. (e) Illustrative conformational transitions of **PPE-Ala-Na** in water/ACN mixtures.

**Figure S42a**-**b** shows that the negative first Cotton effect almost disappears at 40 vol% DMSO, but inverts into a positive one with a value of +33.6 × 10^3^ deg cm^2^ dmol^−1^ in the presence of 50 vol% DMSO, accompanied by a hyperchromic effect at 285 nm. The *ε*_306_/*ε*_285_ and *X*_C_ values slightly increase to 0.57 and 11%, respectively (**Figure S42c**-**d**). So, the foldamer experiences a helix inversion as well as a helix extension (**Figure S42e**). When the DMSO fraction increases to 90 vol%, the foldamer is still at a single-molecular state (**Figure S44**). The first Cotton effect still shows a positive sign. Meanwhile, a further hyperchromic effect at 285 nm with a red-shift to 293 nm can be found, along with a significant increase of the absorption peak at 306 nm. The *ε*_306_/*ε*_285_ and *X*_C_ values are sharply increased to 0.84 and 66%, respectively (**Figure S42c**-**d**), indicative of a partial helix-coil transition. This result can be further confirmed by the emission spectra of **PPE-Ala-Na** in water/DMSO mixtures (**Figure S43**). Unlike THF and ACN, as a strong hydrogen bond competitor,^[49]^ the DMSO molecules will destroy the intramolecular hydrogen bonds between the neighboring pendant amides in the foldamer once the induced helix is formed by L-alanine sodium pendants. Finally, the foldamer possesses a mixture of the random-coil segments and the extended (*P*)-handed helical segments at 90 vol% DMSO (**Figure S42e**).


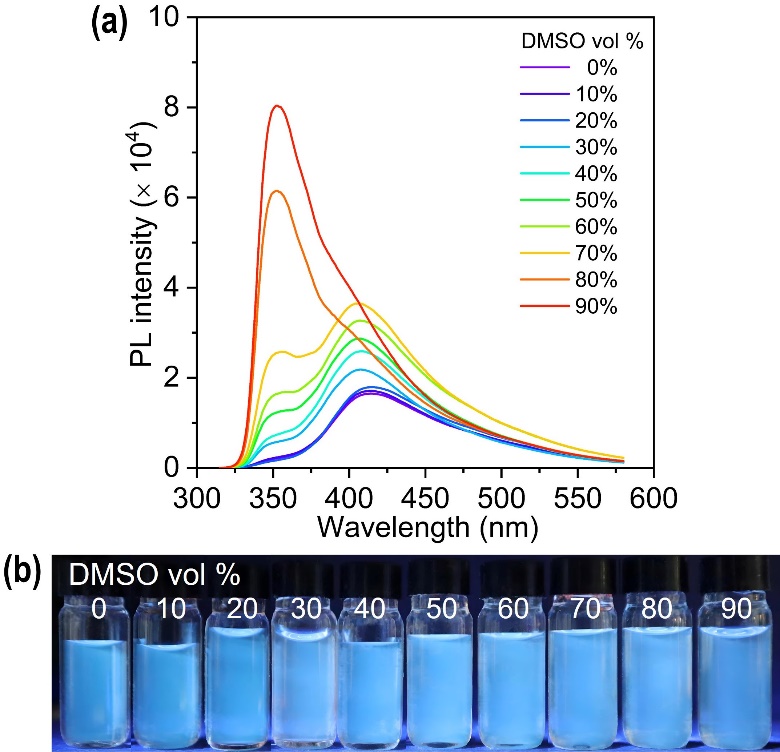


**Figure S43*.*** (a) Fluorescence spectra of **PPE-Ala-Na** in water/DMSO mixtures with different DMSO fractions. [**PPE-Ala-Na**] = 100 μM; excitation wavelength (*λ*_ex_): 295 nm. (b) Fluorescence photographs of **PPE-Ala-Na** in water/DMSO mixtures under 365 nm UV irradiation.


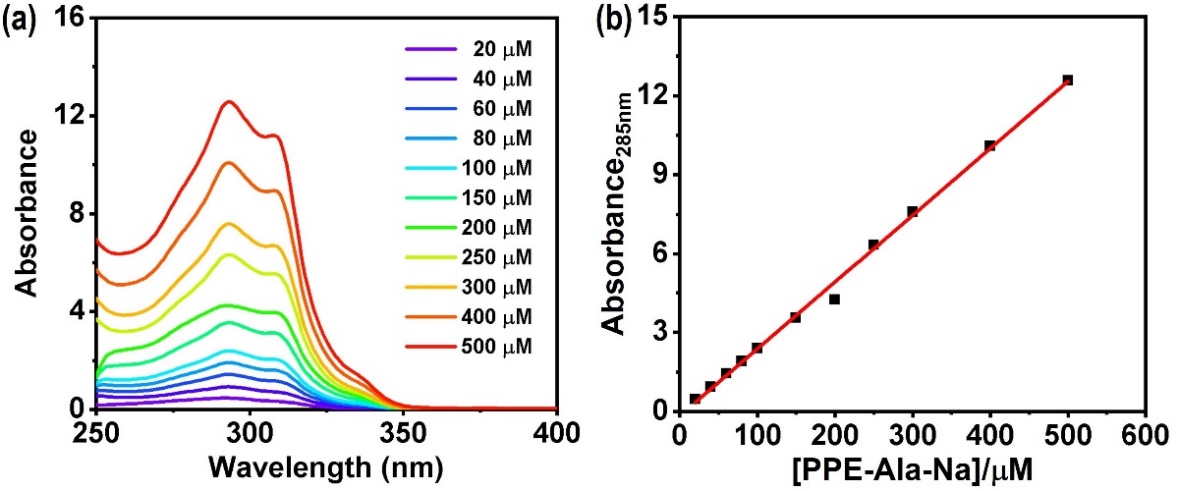


**Figure S44*.*** (a) Concentration-dependent UV−vis absorption spectra of **PPE-Ala-Na** in water/DMSO (10/90, v/v) at room temperature. (b) Plot of the absorption maxima of **PPE-Ala-Na** in water/DMSO (10/90, v/v) versus the concentration of **PPE-Ala-Na**.

**16.** **Binding Behavior of TPEBe-I with PPE-Ala-Na in Water/ACN (99/1, v/v) and Water/DMSO (99/1, v/v)**


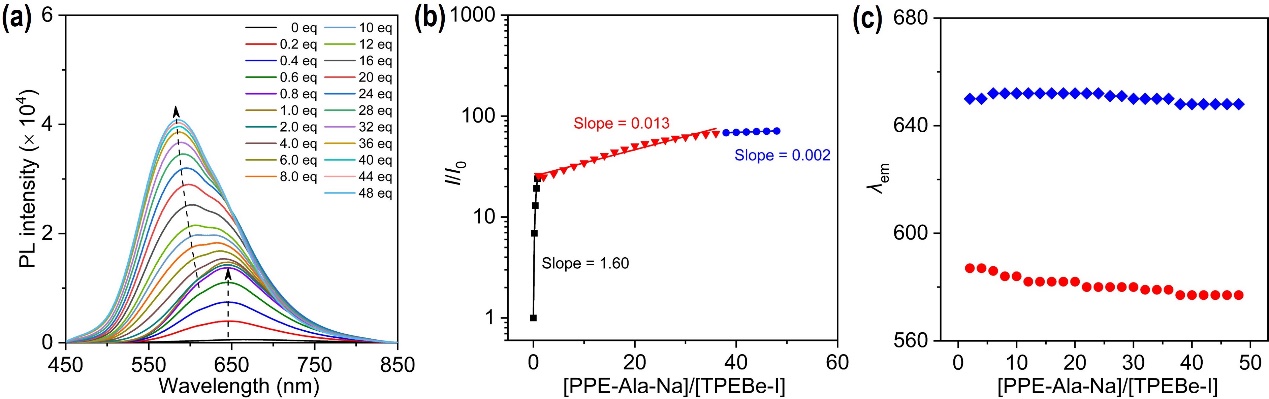


**Figure S45*.*** (a) Fluorescence spectra of **TPEBe-I** in the presence of **PPE-Ala-Na** in water/ACN (99/1, v/v) at room temperature. [**TPEBe-I**] = 1 μM; excitation wavelength (*λ*_ex_): 420 nm. Plots of (b) relative fluorescence intensity (*I*/*I*_0_) and (c) emission wavelength (*λ*_em_) of **TPEBe-I** versus the concentration ratio of **PPE-Ala-Na** to **TPEBe-I**, where *I*_0_ and *I* represent the fluorescence intensity of pure **TPEBe-I** and the fluorescence maximum of **TPEBe-I** in the presence of **PPE-Ala-Na**, respectively. The emission wavelength values were estimated through peak-differentiation-imitating analyses.


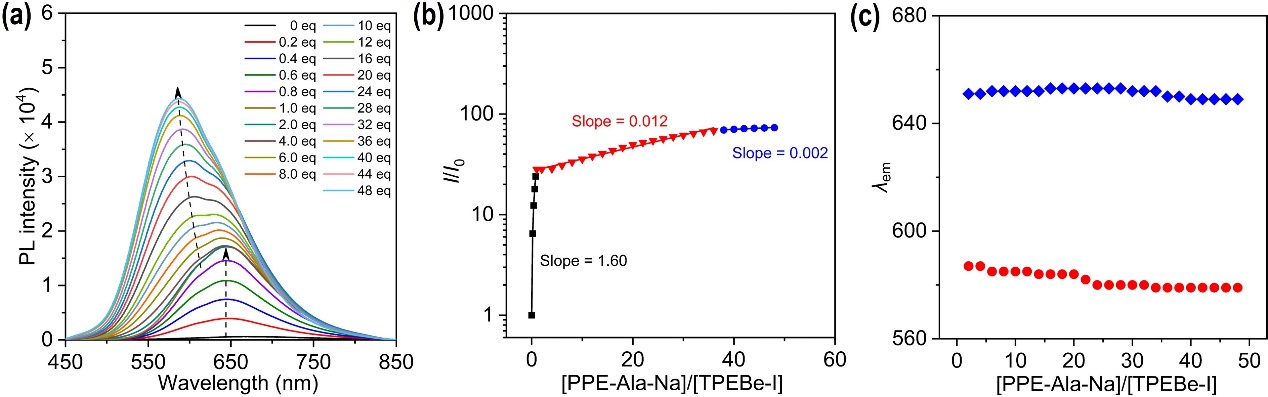


**Figure S46*.*** (a) Fluorescence spectra of **TPEBe-I** in the presence of **PPE-Ala-Na** in water/DMSO (99/1, v/v) at room temperature. [**TPEBe-I**] = 1 μM; excitation wavelength (*λ*_ex_): 420 nm. Plots of (b) relative fluorescence intensity (*I*/*I*_0_) and (c) emission wavelength (*λ*_em_) of **TPEBe-I** versus the concentration ratio of **PPE-Ala-Na** to **TPEBe-I**, where *I*_0_ and *I* represent the fluorescence intensity of pure **TPEBe-I** and the fluorescence maximum of **TPEBe-I** in the presence of **PPE-Ala-Na**, respectively. The emission wavelength values were estimated through peak-differentiation-imitating analyses.


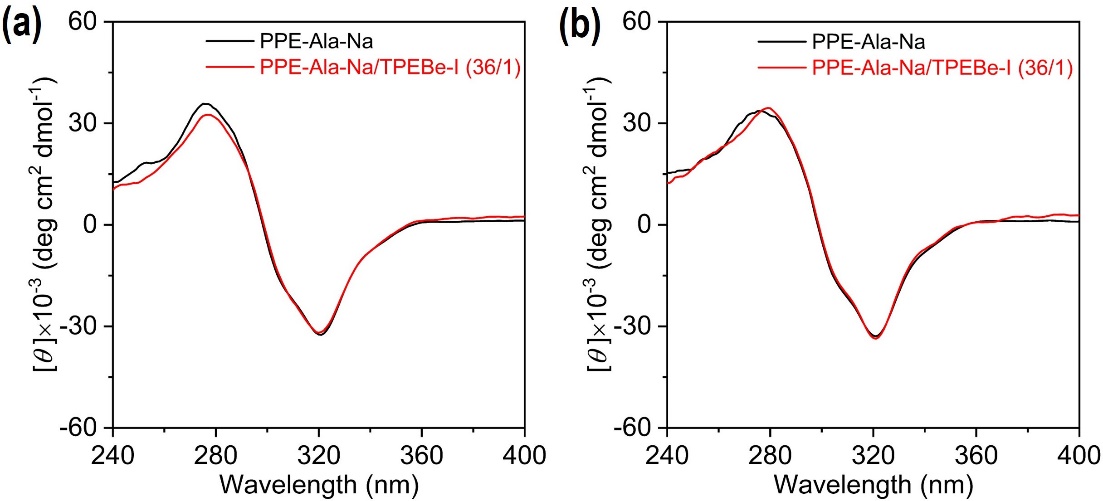


**Figure S47*.*** CD spectra of pure **PPE-Ala-Na** and the mixture between **TPEBe-I** and **PPE-Ala-Na** at a [**TPEBe-I**]/[**PPE-Ala-Na**] ratio of 1/36 in (a) water/ACN (99/1, v/v) and (b) water/DMSO (99/1, v/v) at room temperature. The concentration of pure **PPE-Ala-Na** was 36 μM. [**TPEBe-I**] = 1 μM.


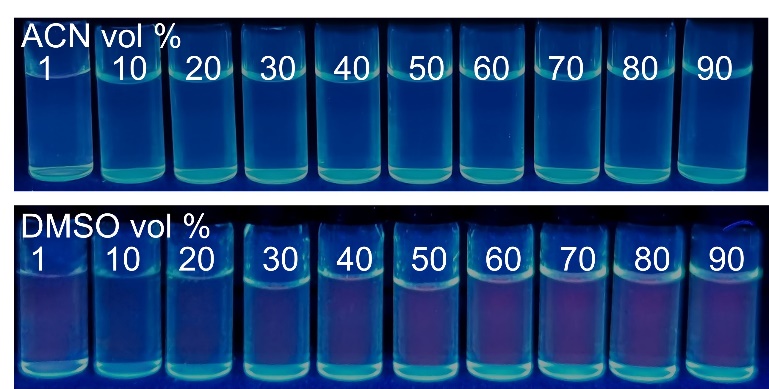


**Figure S48*.*** Fluorescence photographs of pure **TPEBe-I** in water/ACN and water/DMSO mixtures under 365 nm UV irradiation. [**TPEBe-I**] = 1 μM.

**17. Conformational Transitions of PPE-Ala-Na with TPEBe-I in Water/THF and Water/DMSO Mixtures**


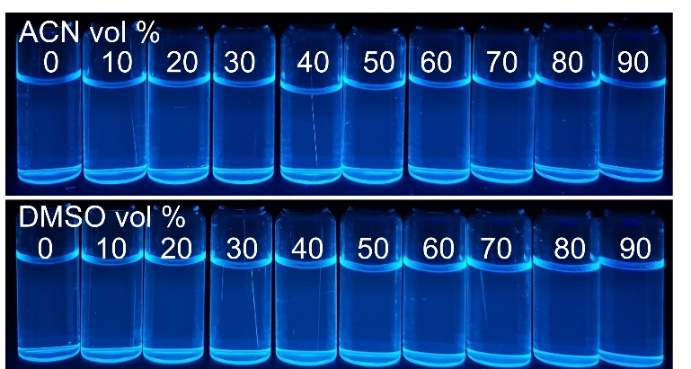


**Figure S49*.*** Fluorescence photographs of pure **PPE-Ala-Na** in water/ACN and water/DMSO mixtures under 365 nm UV irradiation. [**PPE-Ala-Na**] = 36 μM.


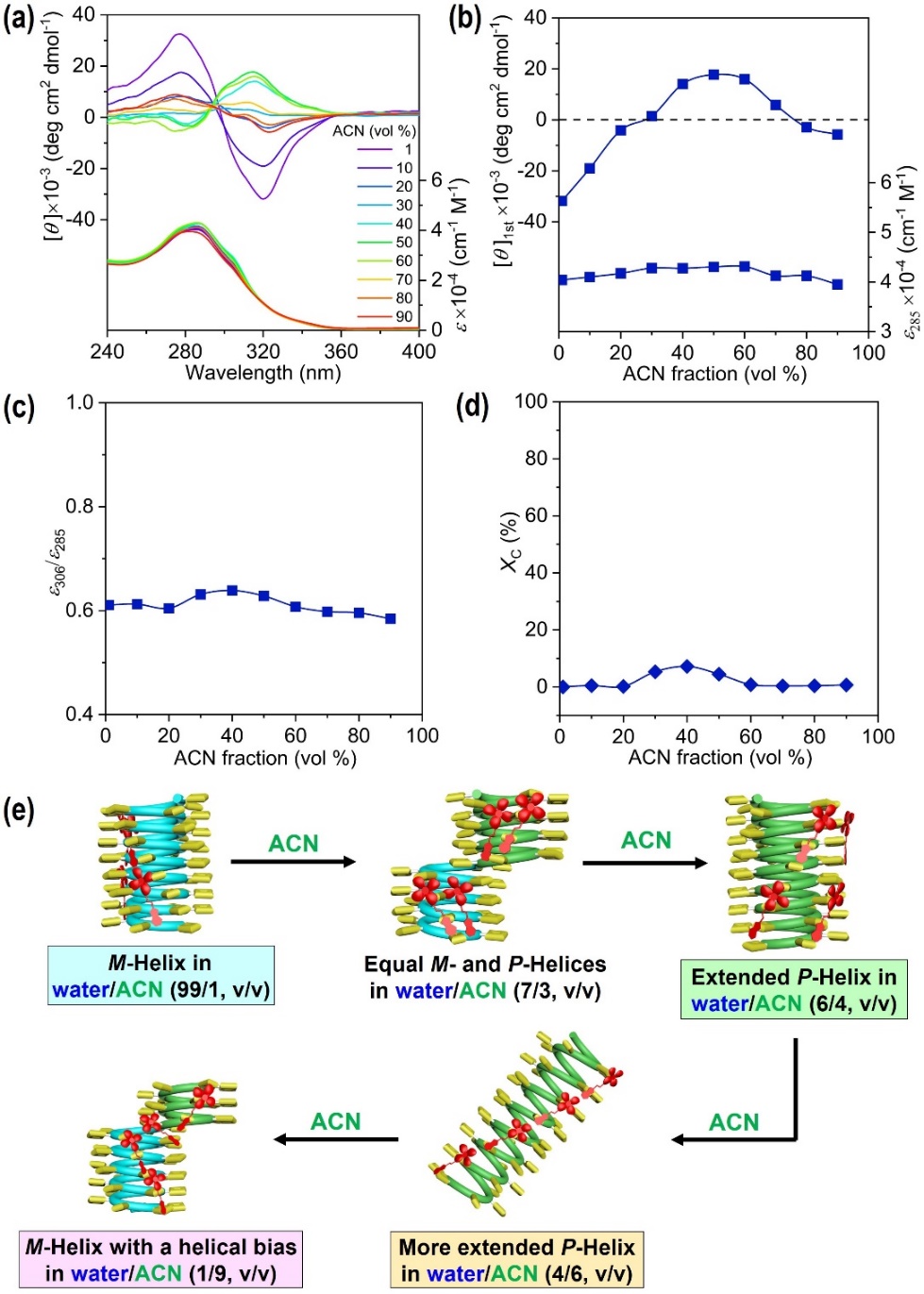


**Figure S50*.*** (a) CD (top) and UV−vis absorption (bottom) spectra of **TPEBe-I** with **PPE-Ala-Na** in water/ACN mixtures with different ACN fractions. Plots of the first Cotton effect intensity (b, top), absorption intensity at 285 nm (b, bottom), absorption intensity ratio at 306 and 285 nm (c), and the content of random-coil conformation (d) versus the volume fraction of ACN. (e) Illustrative conformational transitions of **PPE-Ala-Na** in the presence of **TPEBe-I** in water/ACN mixtures.


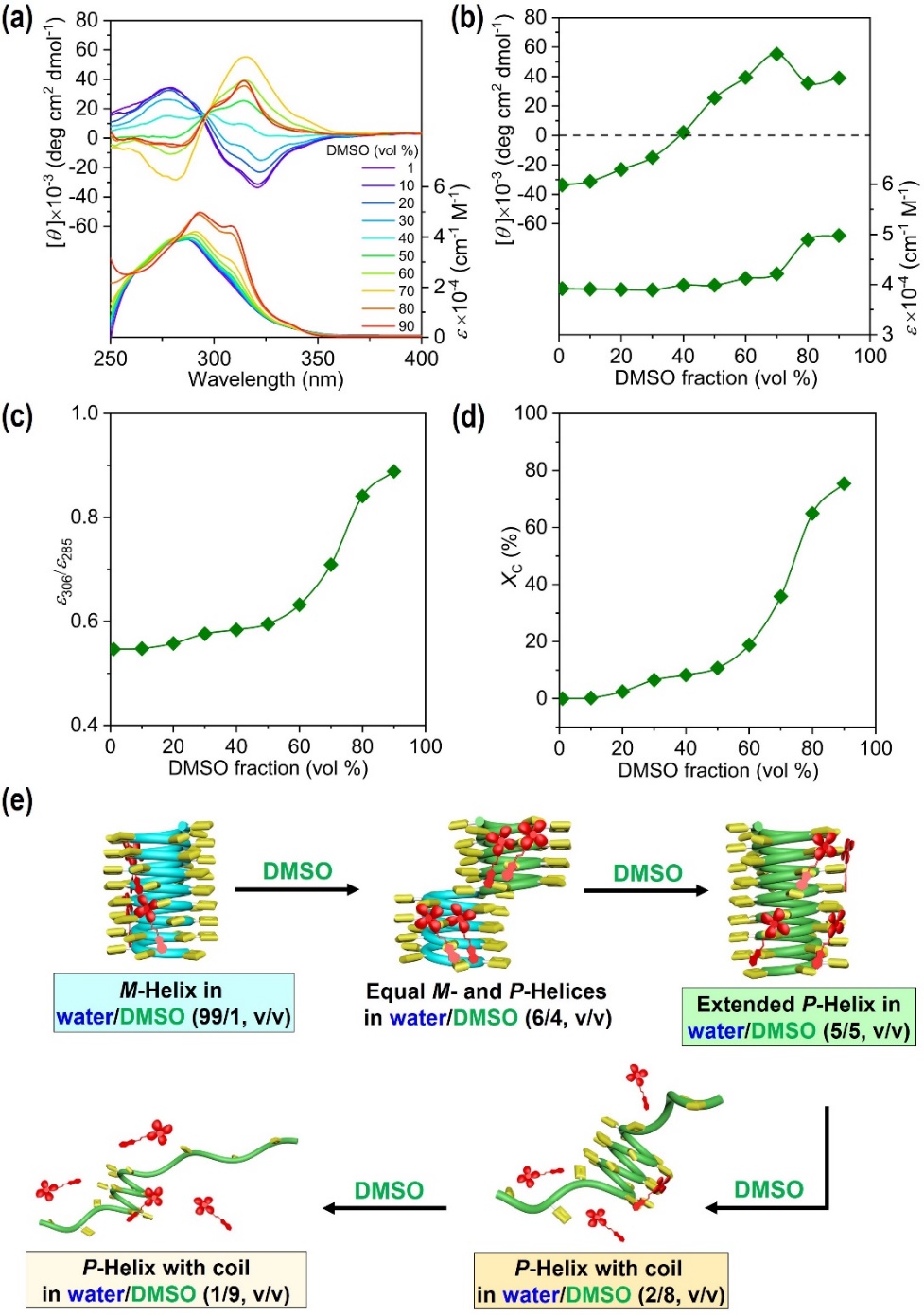


**Figure S51*.*** (a) CD (top) and UV−vis absorption (bottom) spectra of **TPEBe-I** with **PPE-Ala-Na** in water/DMSO mixtures with different DMSO fractions. Plots of the first Cotton effect intensity (b, top), absorption intensity at 285 nm (b, bottom), absorption intensity ratio at 306 and 285 nm (c), and the content of random-coil conformation (d) versus the volume fraction of DMSO. (e) Illustrative conformational transitions of **PPE-Ala-Na** in the presence of **TPEBe-I** in water/DMSO mixtures.

**18. Captions for Supporting Movies**

**Movie S1.** Animation of the MD simulation of the left-handed helically folded model (36-mer) of **PPE-Ala-Na** complexed with **TPEBe-I** (ball-and-stick model) in water/THF (99/1, v/v) at 0–20,000 ps as the production run. The solvent molecules are omitted to simplify the view.

**Movie S2.** Same simulation method as **Movie S1** except for the solvent system of water/THF (80/20, v/v).

**19. Supporting References**

[1] Y. Qiu, H. Hu, D. Zhao, J. Wang, H. Wang, Q. Wang, H. Peng, Y. Liao, X. Xie, *Polymer* **2019**, *170*, 7-15.

[2] N. Zhao, J. W. Y. Lam, H. H. Y. Sung, H. M. Su, I. D. Williams, K. S.Wong, B. Z. Tang, *Chem. Eur. J.* **2014**, *20*, 133-138.

[3] R. A. Smaldone, E.-C. Lin, J. S. Moore, *J. Polym. Sci., Part A: Polym. Chem.* **2010**, *48*, 927-935.

[4] K. Matsuda, M. T. Stone, J. S. Moore, *J. Am. Chem. Soc.* **2002**, *124*, 11836-11837.

[5] M. S. Gin, J. S. Moore, *Org. Lett.* **2000**, *2*, 135-138.

[6] D. J. Hill, J. S. Moore, *Proc. Natl. Acad. Sci. U.S.A.* **2002**, *99*, 5053-5057.

[7] M. Banno, T. Yamaguchi, K. Nagai, C. Kaiser, S. Hecht, E. Yashima, *J. Am. Chem. Soc.* **2012**, *134*, 8718-8728.

[8] A. Y. Toukmaji, J. A. B. Jr, *Comput. Phys. Commun.* **1996**, *95*, 73-92.

[9] J. P. Perdew, K. Burke, M. Ernzerhof, *Phys. Rev. Lett.* **1996**, *77*, 3865-3868.

[10] S. Grimme, J. Antony, S. Ehrlich, H. Krieg, *J. Chem. Phys.* **2010**, *132*, 154104.

[11] J. VandeVondele, M. Krack, F. Mohamed, M. Parrinello, T. Chassaing, J. Hutter, *Comput. Phys. Commun.* **2005**, *167*, 103-128.

[12] G. Lippert, J. Hutter, M. Parrinello, *Theor. Chem. Acc.* **1999**, *103*, 124-140.

[13] S. Goedecker, M. Teter, J. Hutter, *Phys. Rev. B: Condens. Matter Mater. Phys.* **1996**, *54*, 1703-1710.

[14] J. VandeVondele, J. Hutter, *J. Chem. Phys.* **2007**, *127*, 114105.

[15] M. J. Frisch, G. W. Trucks, H. B. Schlegel, G. E. Scuseria, M. A. Robb, J. R. Cheeseman, G. Scalmani, V. Barone, G. A. Petersson, H. Nakatsuji, X. Li, M. Caricato, A. V. Marenich, J. Bloino, B. G. Janesko, R. Gomperts, B. Mennucci, H. P. Hratchian, J. V. Ortiz, A. F. Izmaylov, J. L. Sonnenberg, Williams, F. Ding, F. Lipparini, F. Egidi, J. Goings, B. Peng, A. Petrone, T. Henderson, D. Ranasinghe, V. G. Zakrzewski, J. Gao, N. Rega, G. Zheng, W. Liang, M. Hada, M. Ehara, K. Toyota, R. Fukuda, J. Hasegawa, M. Ishida, T. Nakajima, Y. Honda, O. Kitao, H. Nakai, T. Vreven, K. Throssell, J. A. Montgomery Jr., J. E. Peralta, F. Ogliaro, M. J. Bearpark, J. J. Heyd, E. N. Brothers, K. N. Kudin, V. N. Staroverov, T. A. Keith, R. Kobayashi, J. Normand, K. Raghavachari, A. P. Rendell, J. C. Burant, S. S. Iyengar, J. Tomasi, M. Cossi, J. M. Millam, M. Klene, C. Adamo, R. Cammi, J. W. Ochterski, R. L. Martin, K. Morokuma, O. Farkas, J. B. Foresman, D. J. Fox, Wallingford, CT, **2016**.

[16] J.-D. Chai, M. Head-Gordon, *Phys. Chem. Chem. Phys.* **2008**, *10*, 6615-6620.

[17] S. Páll, M. J. Abraham, C. Kutzner, B. Hess, E. Lindahl, *In Solving Software Challenges for Exascale; Markidis, S., Laure, E., Eds. Lecture Notes in Computer Science; Springer International Publishing: Cham, 2015; pp 3-27* **2015**.

[18] H. J. C. Berendsen, D. v. d. Spoel, R. v. Drunen, *Comput. Phys. Commun.* **1995**, *91*, 43-56.

[19] Mark James Abrahama, Teemu Murtolad, R. Schulz, S. Pálla, J. C. Smith, B. Hess, E. Lindahl, *SoftwareX* **2015**, *1-2*, 19-25.

[20] D. V. D. Spoel, E. Lindahl, B. Hess, G. Groenhof, A. E. Mark, H. J. C. Berendsen, *J. Comput. Chem.* **2005**, *26*, 1701-1718.

[21] T. Lu, http://www.keinsci.com/research/molclus.html (accessed August 23, 2023).

[22] C. Bannwarth, S. Ehlert, S. Grimme, *J. Chem. Theory Comput.* **2019**, *15*, 1652-1671.

[23] J. G. Brandenburg, C. Bannwarth, A. Hansen, S. Grimme, *J. Chem. Phys.* **2018**, *148*, 064104.

[24] L. MartíNez, R. Andrade, E. G. Birgin, J. M. Martínez, *J. Comput. Chem.* **2009**, *30*, 2157-2164.

[25] J. Wang, R. M. Wolf, J. W. Caldwell, P. A. Kollman, D. A. Case, *J. Comput. Chem.* **2004**, *25*, 1157-1174.

[26] M. Schauperl, P. S. Nerenberg, H. Jang, L.-P. Wang, C. I. Bayly, D. L. Mobley, M. K. Gilson, *Commun. Chem.* **2020**, *3*.

[27] J. Tomasi, B. Mennucci, R. Cammi, *Chem. Rev.* **2005**, *105*, 2999-3093.

[28] T. Lu, F. Chen, *J. Comput. Chem.* **2012**, *33*, 580-592.

[29] Z. Liu, W. Xue, Z. Cai, G. Zhang, D. Zhang, *J. Mater. Chem.* **2011**, *21*, 14487-14491.

[30] H. Abe, N. Masuda, M. Waki, M. Inouye, *J. Am. Chem. Soc.* **2005**, *127*, 16189-16196.

[31] Y. Qiu, Y. Zhang, Q. Jiang, H. Wang, Y. Liao, H. Zhou, X. Xie, *Macromolecules* **2022**, *55*, 9057-9065.

[32] K. Maeda, L. Hong, T. Nishihara, Y. Nakanishi, Y. Miyauchi, R. Kitaura, N. Ousaka, E. Yashima, H. Ito, K. Itami, *J. Am. Chem. Soc.* **2016**, *138*, 11001-11008.

[33] K. Suda, K. Akagi, *Macromolecules* **2011**, *44*, 9473-9488.

[34] E. Espinosa, E. Molins, C. Lecomte, *Chem. Phys. Lett.* **1998**, *285*, 170-173.

[35] H. Goto, J. M. Heemstra, D. J. Hill, J. S. Moore, *Org. Lett.* **2004**, *6*, 889-892.

[36] M. T. Stone, J. M. Heemstra, J. S. Moore, *Acc. Chem. Res.* **2006**, *39*, 11-20.

[37] R. Sakai, S. Okade, E. B. Barasa, R. Kakuchi, M. Ziabka, S. Umeda, K. Tsuda, T. Satoh, T. Kakuchi, *Macromolecules* **2010**, *43*, 7406-7411.

[38] C. Tan, M. R.Pinto, M. E. Kose, I. Ghiviriga, K. S.Schanze, *Adv. Mater.* **2004**, *16*, 1208-1212.

[39] Y. Qiu, H. Yan, J. Wang, Q. Jiang, H. Wang, H. Peng, Y. Liao, X. Xie, B. E. Brycki, *Polymer* **2020**, *188*, 122135.

[40] M. Waki, H. Abe, M. Inouye, *Angew. Chem. Int. Ed.* **2007**, *46*, 3059-3061.

[41] Y. Qiu, S. Cao, C. Sun, Q. Jiang, C. Xie, H. Wang, Y. Liao, X. Xie, *Polym. Chem.* **2022**, *13*, 4569-4577.

[42] J. C. Nelson, J. G. Saven, J. S. Moore, P. G. Wolynes, *Science* **1997**, *277*, 1793-1796.

[43] Y. Gu, L. Liu, Y. Wang, C. Zhang, H. Dong, *Macromolecules* **2020**, *53*, 10734-10743.

[44] S. Arias, M. Núñez-Martínez, E. Quiñoá, R. Riguera, F. Freire, *Polym. Chem.* **2017**, *8*, 3740-3745.

[45] L. Liu, N. Ousaka, M. Horie, F. Mamiya, E. Yashima, *Chem. Commun.* **2016**, *52*, 11752-11755.

[46] S. Leiras, F. Freire, J. M. Seco, E. Quiñoá, R. Riguera, *Chem. Sci.* **2013**, *4*, 2735-2743.

[47] S. Kawabata, N. Ousaka, E. Yashima, *Chem. Commun.* **2018**, *54*, 2417-2420.

[48] S. Lahiri, J. L. Thompson, J. S. Moore, *J. Am. Chem. Soc.* **2000**, *122*, 11315-11319.

[49] J. Bai, H. Yin, Y. Zhang, C. Zhang, L. Liu, X. Xu, *Eur. Polym. J.* **2017**, *87*, 380-388.
